# Supplementary material for: A Phase I/II randomized trial of H56:IC31 vaccination and adjunctive cyclooxygenase-2-inhibitor treatment in tuberculosis patients
Source: Nat Commun. 2021 Nov 22;12:6774. doi: 10.1038/s41467-021-27029-6 (PMC8608791; doi:10.1038/s41467-021-27029-6)
Supplement: Supplementary file 1 — Supplementary Information [file 41467_2021_27029_MOESM1_ESM.pdf]

SUPPLEMENTARY INFORMATION

A Phase I/II randomized trial of H56:IC31 vaccination and adjunctive Cyclooxygenase-2-inhibitor treatment in Tuberculosis patients

ClinicalTrials.gov. No: NCT02503839

Original and revised inclusion and exclusion criteria

Protocol version 6.0 12-10-2016.

1.1 Selection of Study Population

Patients admitted to the Dep. of Infectious Diseases and Dep. of Pulmonary Medicine, OUS and with confirmed active pulmonary and/or extrapulmonary TB disease infected with drug-sensitive *Mtb* strains will be included. If there are difficulties in the recruitment of patients at OUS, TB patients will be recruited in collaboration with other hospitals in the Oslo region or our partners from Haukeland University Hospital.

1.2 Number of Patients

A total of 40 patients will be included, 10 in each of the study arms.

1.3 Inclusion Criteria

<u>Subjects may be included in the study if they meet all of the following criteria:</u>

- Age between 18 - 70 years at the time of randomization
- Microbiologically confirmed pulmonary and/or extrapulmonary TB as defined in Table A1 (culture and/or PCR + susceptibility testing).
- Drug sensitive *Mtb* strains (except single resistance where fully adequate anti-TB chemotherapy regimen could be provided).
- Is willing and likely to comply with the trial procedures and is prepared to grant authorized persons access to their medical record.
- Has completed the written informed consent process prior to the start of screening evaluations.
- Females at childbearing age must agree to avoid pregnancy from screening and during the trial.

Female study participants at fertile age (not sterilized and still menstruating or within 1 year of the last menses if menopausal) will be informed about the risk of using the IMPs if pregnant or breastfeeding. The study investigator will perform an individual evaluation of each participant to secure acceptable methods of avoiding pregnancy during the study.

Acceptable methods of avoiding pregnancy include a sterile sexual partner, sexual abstinence (not engaging in sexual intercourse), progestogen-only hormonal contraception associated with inhibition of ovulation (oral, injection, transdermal patch, or implant), intrauterine device (IUD), or a combination of male condom with either cap, diaphragm or sponge with spermicide ("double barrier methods").

**Supplementary Table 1. Forms of extrapulmonal TB that can be included:**
Bone/joint (including soft tissue affection/abscess)
Lymphadenitis
Pleuritis
Abdominal (peritoneal and intestinal)
Urogenital

1.4 Exclusion Criteria

Subjects must be excluded from participating in this study if they meet any of the following criteria:

1.4.1 Study-specific exclusion criteria

- Disseminated (miliary) TB.
- Evidence of a new acute illness that may compromise the safety of the subject in the trial on study day 0.
- History of autoimmune disease or immunosuppression.
- History or laboratory evidence of any possible immunodeficiency state.
- Anemia (< 9 g/100 mL)
- HIV sero-positivity.
- Chronic hepatitis B (HBs antigen positive) with increased liver transaminases (ASAT, ALAT).
- Chronic hepatitis C (HCV RNA positive).
- Concomitant or sporadic use of NSAID or corticosteroids (>2 times per week)
- Other immune modulating therapies including DMARDs.
- Total cholesterol >7 mmol/L.
- Hypertension >140/90 mm Hg (treated or untreated) or treated with >1 antihypertensive drug at any blood pressure.
- Cardiovascular events or stroke in parents, siblings or off-springs occurring <55 years of age
- Serum creatinine above reference levels (females >90 µmol/L; males >105 µmol/L), see reduced creatinine clearance in Section 4.4.2).
- Known diabetes mellitus type I or diabetes mellitus type II with HbA1c >7%.
- Pregnancy (documented S-hCG >5 IU/l for females at childbearing age).
- Breastfeeding.
- Known hypersensitivity for vaccines or vaccine adjuvants.

1.4.2 Exclusion criteria according to the SPC on Arcoxia from Norwegian Medicines Agency

- Known hypersensitivity for etoricoxib or etoricoxib tablet substances
- Known hypersensitivity for sulphonamides
- Active peptic ulcer or gastrointestinal haemorrhage
- Patients with history of asthma, acute rhinitis, nasal polyps, angioneurotic oedema, urticaria or other allergic reactions after taking acetyl salicylic acid or NSAID including COX-2i.
- Moderate/severe deranged liver function (Child-Pugh >7).
- Creatinine clearance <30 ml/min
- Inflammatory bowel disease
- Heart failure (NYHA II-IV)
- Established ischaemic heart disease, peripheral arteriosclerosis and/or cerebrovascular disease, including previous myocardial infarction, angina pectoris, unstable angina, PCI or coronary bypass, previous transitory ischemic attack or apoplexia/stroke.

1.5 Criteria for receiving H56:IC31 vaccine

<u>Subjects may receive H56:IC31 vaccination (arm #2 and #4) if they meet the following criteria:</u>

Pulmonary TB:

1. Sputum obtained prior to 1th immunization at day 84 must be *Mtb* negative evaluated by at least two consecutive AFS or PCR/GeneXpert at least 7 days apart.
2. Clinical improvement with normal vital signs (blood pressure, temperature and pulse) and reduced inflammatory blood parameters (CRP and ESR) compared to baseline.

Extrapulmonary TB:

1. Documented reduction in the extent of TB disease at the infectious site(s) within day 84 evaluated by physical and/or radiological examination (table A2).
2. Clinical improvement with normal vital signs (blood pressure, temperature and pulse), improvement of any TB related symptoms to Grade 1-3 (table A2), stable or increased body-weight and reduced inflammatory blood parameters (CRP, ESR and WBC counts) compared to baseline.

Toxicity Table –TBCOX2

Note: From final US FDA guidance: "Toxicity Grading Scale for Healthy Adult and Adolescent Subjects Enrolled in Preventive Vaccine Clinical Trials (September 2007) and for some values Division of AIDS (DAIDS) Table for Grading the Severity of Adult and Pediatric Adverse Events Version 2.0" (November 2014). The laboratory values provided in the tables serve as guidelines and are dependent upon institutional normal parameters laboratory values. Institutional normal reference ranges are provided to demonstrate that they are appropriate (OUH reference units).

Supplementary Table 2.

**Local Reaction to Injectable Product**	**Mild (Grade 1)**	**Moderate (Grade 2)**	**Severe (Grade 3)**	**Potentially Life Threatening (Grade 4)**
Pain	Does not interfere with activity	Repeated use of non-narcotic pain reliever > 24 hours or interferes with activity	Any use of narcotic pain reliever or prevents daily activity	Emergency room (ER) visit or hospitalization
Tenderness	Mild discomfort to touch	Discomfort with movement	Significant discomfort at rest	ER visit or hospitalization
Erythema/Redness*	2·5 – 5 cm	5·1 – 10 cm	> 10 cm	Necrosis or exfoliative dermatitis
Induration/Swelling**	2·5 – 5 cm and does not interfere with activity	5·1 – 10 cm or interferes with activity	> 10 cm or prevents daily activity	Necrosis

* In addition to grading the measured local reaction at the greatest single diameter, the measurement should be recorded as a continuous variable.
** Induration/Swelling should be evaluated and graded using the functional scale as well as the actual measurement

**Vital Signs ***	**Mild (Grade 1)**	**Moderate (Grade 2)**	**Severe (Grade 3)**	**Potentially Life Threatening (Grade 4)**
Fever (°C) **	38·0 – 38·4	38·5 – 38·9	39·0 – 40	> 40
Tachycardia - beats per minute	101 – 115	116 – 130	> 130	ER visit or hospitalization for arrhythmia
Bradycardia - beats per minute***	50 – 54	45 – 49	< 45	ER visit orhospitalization for arrhythmia
Hypertension (systolic) - mmHg	141 – 150	151 – 155	> 155	ER visit or hospitalization for malignant hypertension
Hypertension (diastolic) - mmHg	91 – 95	96 – 100	> 100	ER visit or hospitalization for malignant hypertension
Hypotension (systolic) – mmHg	85 – 89	80 – 84	< 80	ER visit or hospitalization for hypotensive hypotensive shock
Respiratory Rate – breaths per minute	17 – 20	21 – 25	> 25	Intubation

* Subject should be at rest for all vital sign measurements.
** Body temperature, measured by ear thermometer
*** When resting heart rate is between 60 – 100 beats per minute. Use clinical judgement when characterizing bradycardia among some healthy subject populations, for example, conditioned athletes.

Systemic (General)	Mild (Grade 1)	Moderate (Grade 2)	Severe (Grade 3)	Potentially Life Threatening (Grade 4)
Nausea/vomiting	No interference with activity or 1 – 2 episodes/24 hours	Some interference with activity or > 2 episodes/24 hours	Prevents daily activity, requires outpatient IV hydration	ER visit or hospitalization for hypotensive shock
Diarrhea	2 – 3 loose stools or < 400g/24 hours	4 – 5 stools or 400 – 800g/24 hours	6 or more watery stools or > 800g/24 hours or requires outpatient IV hydration	ER visit or hospitalization
Headache	No interference with activity	Repeated use of non-narcotic pain reliever > 24 hours or some interference with activity	Significant; any use of narcotic pain reliever or prevents daily activity	ER visit or hospitalization
Fatigue	No interference with activity	Some interference with activity	Significant; prevents daily activity	ER visit or hospitalization
Myalgia	No interference with activity	Some interference with activity	Significant; prevents daily activity	ER visit or hospitalization
Illness or clinical adverse event (as defined according to applicable regulations)	No interference with activity	Some interference with activity not requiring medical intervention	Prevents daily activity and requires medical intervention	ER visit or hospitalization

Serum	Reference ranges adults (OUH)	Mild (Grade 1)	Moderate (Grade 2)	Severe (Grade 3)	Potentially Life Threatening (Grade 4)
Sodium-hyponatremia mmol/L*	137-144	130-134	125-129	121-124	≤ 120
Sodium-hypernatremia mmol/L*	137-144	146-149	150-153	154-159	≥ 160
Potassium-hyperkalemia mmol/L*	3·6-4·6	5·6-5·9	6·0-6·4	6·5-6·9	≥7
Potassium-hypokalemia mmol/L*	3·6-4·6	3·0-3·4	2·5-2·9	2·0-2·4	<2·0
Glucose-hypoglycemia mmol/L (random) **	4·0-6·0	3·6-3·8	3·0-3·5	2·5-2·9	<2·5
Glucose-hyperglycemia mmol/L (random) **	4·0-6·0	6·1-6·8	6·9-11·0	>11·0	Insulin requirement or hyperosmolar coma
Creatinine (µmol/L)**	F: 45-95<br>M: 60-105	K: 96-145<br>M: 106-145	146-170	171-208	> 208 or requires dialysis
Calcium-hypocalcemia mmol/L**	2·15-2·51	2·00-2·10	1·87-1·99	1·75-1·86	<1·75
Calcium -hypercalcemia mmol/L**	2·15-2·51	2·63-2·76	2·77-2·88	2·89-3·00	>3·00
Creatinine Kinase (CK) U/L***	F: 35-210<br>M (18-49y): 50-400<br>M (>50y): 40-280	1·25 – 1·59 x ULN	1·6-3·0 x ULN	3·1-10 x ULN	>10 x ULN
ASAT (U/L) Increase by factor***	K: 15-35<br>M: 15-45	1·1-2·5 x ULN	2·6-5·0 x ULN	5·1-10 x ULN	>10 x ULN
ALAT (U/L) Increased by factor ***	K: 10-45<br>M: 10-70	1·1-2·5 x ULN	2·6-5·0 x ULN	5·1-10 x ULN	>10 x ULN
Lactate dehydrogenase (LD) U/L**	105-205	1·1-2·5 x ULN	2·6-5·0 x ULN	5·1-10 x ULN	>10 x ULN
Bilirubin µmol/L***	5-25	1·1-1·5 x ULN	1·6-2·0 x ULN	2·1-3·0 x ULN	>3·0 x ULN
Cholesterol mmol/L*	18-29y: 2·9-6·1<br>30-49y: 3·3-6·9<br>> 50y: 3·9-7·8	6·0-6·3	6·4-7·8	>7·8	NA
Pancreatic enzyme (amylase) U/L***	10-65	1·1-1·5 x ULN	1·6-2·0 x ULN	2·1-5·0 x ULN	>5·0 x ULN

2 HEMATOLOGY	Reference ranges adults (OUH)	Mild (grade 1)	Moderate (Grade2)	Severe (Grade 3)	Potentially Life Threatening (Grade 4)
Hemoglobin (g/100mL) - (Female)*	11·7-15·3	10·0-10·9	9·0-9·9	7·0-8·9	<7
Hemoglobin (g/100mL) - (Female) *change from baseline value****	11·7-15·3	Any decrease -1·5	1·6-2·0	2·1-5·0	>5·0
Hemoglobin (g/100mL) - (Male) **	13·4-17·0	12·0-13·0	10·5-11·9	8·5-10·4	<8·5
Hemoglobin (g/100mL) (Male) *change from baseline value****	13·4-17·0	Any decrease -1·5	1·6-2·0	2·1-5·0	>5·0
WBC (10^9/L) - increase***	3·5-10·0	10·8-15·0	15·1-20·0	20·1-25	>25
WBC (10^9/L) - decrease***	3·5-10·0	2·5-3·5	1·5-2·4	1·0-1·4	<1·0
Platelets (10^9/L) - decrease***	145-390	125-140	100-124	25-99	<25
Lymphocytes (10^9/L) - decrease***	1·1-3·3	0·8-1·0	0·5-0·7	0·3-0·4	<0·3

OUH= Oslo University Hospital
ULN= Upper Level Limit of Normal range
* Division of AIDS (DAIDS) Table for Grading the Severity of Adult and Pediatric Adverse Events. Version 2.0 - November 2014. Modified according to local laboratory reference ranges for Cholesterol and Uric acid.
** Modified FDA according to local laboratory reference ranges.
*** FDA; Guidance for Industry. Toxicity Grading Scale for Healthy Adult and Adolescent Volunteers enrolled in preventive vaccine clinical trials. US Department of health and Human Services. Food and Drug Administration. Center for biologics Evaluation and Research. September 2007.

Priority ranking of immunogenicity readouts according to statistical plan

Definition of outcomes described in Tables 1-3

- **Sin cytokine:** Cells expressing only one cytokine: sin_IFNγ or sin_IL2 or sin_TNFα*.
- **Duo cytokine:** Cells expressing two cytokines simultaneously: duo_IFNγ/IL2 or duo_IFNγ/TNFα*or duo_IL2/TNFα*.
- **Tri cytokines:** Cells expressing three cytokines simultaneously: tri_IFNγ/IL2/TNFα*.
- **Tot cytokines:** SUM of cytokine combinations expressing cytokine of interest (sin_cytokines PLUS duo_cytokines PLUS tri_cytokines*): tot_IFNγ or tot_IL2 or tot_TNFα*
- **All cytokines for Whole Blood ICS (Cytokine^{+}):** SUM of the following cytokine combinations: tot_IFNγ PLUS duo_IL2/TNFα PLUS sin_IL2 PLUS sin_TNFα.
- **All cytokines for Fluorospot (Cytokine^{+}):** SUM of the following cytokine combinations: tot_IFNγ PLUS tot_IL2 MINUS duo_IFNγ/IL2.:

Supplementary Table 3. Hierarchal ranking of primary and secondary priority outcomes and definition of hypotheses that will be tested comparing etoricoxib vs. controls. Formal testing will be performed on the primary priority outcomes only, tertiary priority outcomes are not listed here.

Method	Outcome/readout	Time interval (days)	Comparison
**Primary priority**			
FLUOROSPOT (FS)	All_cytokine responses to SUM (Ag85+ESAT6+CFP10)	0-84	Group (1+4) vs (2+3)
	Tot_IFNγ responses to SUM (Ag85+ESAT6+CFP10)	0-84	Group (1+4) vs (2+3)
Whole blood (WB) intracellular cytokine analyses	All_cytokine CD4 responses to PPD	0-84	Group (1+4) vs (2+3)
	All_cytokine CD4 responses to SUM (Ag85+ESAT6+Rv2660)	0-84	Group (1+4) vs (2+3)
**Secondary priority**			
FLUOROSPOT (FS)	All_cytokine responses to ESAT6	0-84	Group (1+4) vs (2+3)
	All_cytokine responses to Ag85	0-84	Group (1+4) vs (2+3)
	All_cytokine responses to CFP10	0-84	Group (1+4) vs (2+3)
	Duo_IL2/IFNγ responses to SUM (Ag85+ESAT6+CFP10)	0-84	Group (1+4) vs (2+3)
Whole blood (WB) intracellular cytokine analyses	All_cytokine CD4/CD8 responses to ESAT6	0-84	Group (1+4) vs (2+3)
	All_cytokine CD4/CD8 responses to Ag85	0-84	Group (1+4) vs (2+3)
	All_cytokine CD4/CD8 responses to Rv2660	0-84	Group (1+4) vs (2+3)
	Tri_IFNγ/IL2/TNFα CD4 responses to PPD	0-84	Group (1+4) vs (2+3)
	Tri_IFNγ/IL2/TNFα CD4 responses to SUM (Ag85+ESAT6+Rv2660)	0-84	Group (1+4) vs (2+3)
	Duo_IL2/IFNγ CD4 responses to SUM (Ag85+ESAT6+Rv2660)	0-84	Group (1+4) vs (2+3)
	Duo_IL2/IFNγ CD4 responses to PPD	0-84	Group (1+4) vs (2+3)
	All_cytokines CD8 responses to PPD	0-84	Group (1+4) vs (2+3)
	All_ cytokines CD8 responses to SUM (Ag85+ESAT6+Rv2660)	0-84	Group (1+4) vs (2+3)

Supplementary Table 4. Hierarchal ranking of primary and secondary priority outcomes and definition of hypotheses that will be tested comparing H56:IC31 vs. controls. Formal testing will be performed on the primary priority outcomes only, tertiary priority outcomes are not listed.

Method	Outcome/readout	Time interval (days)	Comparison
**Primary priority**			
FLUOROSPOT (FS)	All_cytokine responses to H56	84-154	Group 2 vs 3
	Tot_IFNγ responses to H56	84-154	Group 2 vs 3
	All_cytokine responses to SUM (Ag85+ESAT6)	84-154	Group 2 vs 3
Whole blood (WB) intracellular cytokine analyses	All_cytokine CD4 responses to SUM (Ag85+ESAT6+Rv2660)	84-154	Group 2 vs 3
Serology/antibody measurements	IgG antibody responses to H56: IC31	84-154	Group 2 vs 3
**Secondary priority**			
FLUOROSPOT (FS)	All_cytokine responses to ESAT6	84-154	Group 2 vs 3
	All_cytokine responses to Ag85	84-154	Group 2 vs 3
	All_cytokine responses to CFP10	84-154	Group 2 vs 3
	Duo_IL2/IFNγ responses to H56	84-154	Group 2 vs 3
	Tot_IFNγ responses to SUM (Ag85+ESAT6)	84-154	Group 2 vs 3
	Duo_IL2/IFNγ responses to SUM (Ag85+ESAT6)	84-154	Group 2 vs 3
Whole blood (WB) intracellular cytokine analyses	All_cytokine CD4/CD8 responses to ESAT6	84-154	Group 2 vs 3
	All_cytokine CD4/CD8 responses to Ag85	84-154	Group 2 vs 3
	All_cytokine CD4/CD8 responses to Rv2660	84-154	Group 2 vs 3
	All_cytokine CD4/CD8 responses to PPD	84-154	Group 2 vs 3
	Tri_IFNγ/IL2/TNFα CD4 responses to SUM (Ag85+ESAT6+Rv2660)	84-154	Group 2 vs 3
	Duo_IL2/IFNγ CD4 responses to SUM (Ag85+ESAT6+Rv2660)	84-154	Group 2 vs 3
	All_cytokine CD8 responses to SUM (Ag85+ESAT6+Rv2660)	84-154	Group 2 vs 3

Supplementary Table 5. Hierarchal ranking of primary and secondary priority outcomes and definition of hypotheses that will be tested comparing etoricoxib+ H56:IC31 vs. H56:IC31 alone. Formal testing will be performed on the primary priority outcomes only, tertiary priority outcomes are not listed.

Method	Outcome/readout	Time interval (days)	Comparison
**Primary priority**			
FLUOROSPOT (FS)	All_cytokine responses to H56	84-154	Group 4 vs 2
	Tot_IFNγ responses to H56	84-154	Group 4 vs 2
	All_cytokine responses to SUM (Ag85+ESAT6)	84-154	Group 4 vs 2
Whole blood (WB) intracellular cytokine analyses	All_cytokine CD4 responses to SUM (Ag85+ESAT6+Rv2660)	84-154	Group 4 vs 2
Serology/antibody measurements	IgG antibody responses to H56:IC31	84-154	Group 4 vs 2
**Secondary priority**			
FLUOROSPOT (FS)	All_cytokine responses to ESAT6	84-154	Group 4 vs 2
	All_cytokine responses to Ag85	84-154	Group 4 vs 2
	All_cytokine responses to CFP10	84-154	Group 4 vs 2
	Duo_IL2/IFNγ responses to H56	84-154	Group 4 vs 2
	Tot_IFNγ FS responses to SUM (Ag85+ESAT6)	84-154	Group 4 vs 2
	Duo_IL2/IFNγ FS responses to SUM (Ag85+ESAT6)	84-154	Group 4 vs 2
Whole blood (WB) intracellular cytokine analyses	All_cytokine CD4/CD8 responses to ESAT6	84-154	Group 4 vs 2
	All_cytokine CD4/CD8 responses to Ag85	84-154	Group 4 vs 2
	All_cytokine CD4/CD8 responses to Rv2660	84-154	Group 4 vs 2
	All_cytokine CD4/CD8 responses to PPD	84-154	Group 4 vs 2
	Tri_IFNγ/IL2/TNFα CD4 responses to SUM (Ag85+ESAT6+Rv2660)	84-154	Group 4 vs 2
	Duo_IL2/IFNγ CD4 responses to SUM (Ag85+ESAT6+Rv2660)	84-154	Group 4 vs 2
	All_cytokine CD8 responses to SUM (Ag85+ESAT6+Rv2660)	84-154	Group 4 vs 2

Methods – immunogenicity analyses

PBMC sample processing
Peripheral blood mononuclear cells (PBMCs) were isolated from 8 mL whole blood drawn in heparinized cell preparation tubes (BD Vacutainer® CPT™) solution by standard venipuncture. Samples were processed within 2 hours of drawing by centrifugation according to the manufacturer`s recommendations, washed with RPMI (Lonza / BioWhittaker) and cryopreserved in equal amounts of RPMI supplemented with 50% heat-inactivated fetal bovine serum (FBS) (Gibco by Life Technologies) and FBS with 20% dimethyl sulfoxide (DMSO) (Sigma Aldrich). Cells were immediately frozen at -80°C in CoolCell™ containers (Sigma Aldrich) and transferred to -150°C for storage. PBMCs were thawed in 37°C by placing cryovials directly from freezer into a waterbath at 37°C for 1-2 minutes until the cell suspension detached from the wall of the cryovial and transferred to 15mL tubes containing 100µL pre-warmed RPMI 1640 enriched with 10% FBS and 1% PenStepGlutamine (100X). Cryovials were washed out with 100µL medium followed by drop-wise resuspension of cell suspension in 1mL medium and addition of medium to total 10mL for washing. Supernatants were discarded after centrifugation and cell pellet resuspended in total 1mL medium before incubation for 4 hours (37°C, 5% CO_2). Cell count and viability were assessed on stained cells using Muse™ Count & Viability kit and analyzed by the Muse™ Cell Analyzer (Merck, Germany).

Fluorescence immuno-spot (Fluorospot) assay (IFNγ/IL-2)
Peripheral blood mononuclear cells (PBMCs) were isolated from fresh blood (BD Vacutainer® CPT™) and $3x10^5$ cryopreserved PBMCs per well (Human IFNγ/IL-2 pre-coated FluoroSpot plates, 96-wells, Mabtech AB, Sweden), were set up in triplicates for 17 hour stimulation with co-stimulant (anti-CD28 0.1mg/ml, Mabtech AB, Sweden) and the following individual stimulants; ESAT-6, (2 µg/ml), Ag85, (2 µg/ml) (both 15mer overlapping peptide, Genscript, HK limited; >85% purity), culture filtrate protein-10 (CFP-10) (2µg/mL, 15mer overlapping peptide pools, >80% purity; Schäfer, Hadsund, Denmark), and the H56 fusion protein at 5µg/mL (Statens Serum institute, SSI). Samples were processed on a CTL Analyzer (LCC Cellular Technology Limited) according to the manufacturer's instructions and spot-forming units (SFU) were automatically counted applying the ImmunoSpot Software v.5.1 (2012) prior to manual quality control. A valid result required, >12 SFU for IFNγ in positive control (anti-CD3 0·1mg/ml, Mabtech AB, Sweden) and ≥ 2 times the SFU in the negative control (RPMI 1640 L-Glutamine, Lonza/Bio Whittaker) (Essone et al., 2014). The immunological readouts were denoted as described in appendix (pp 7-8).

Whole blood intracellular cytokine staining (WB-ICS) assay
Within 75 min of sampling, 1 ml of WB were incubated in the presence of co-stimulants (anti-CD28, 0,25µg/mL and anti-CD49d 0·25µg/mL) with separate peptide pools (ESAT-6, Ag85b and Rv2660c (2µg/mL, 15mer overlapping peptide, Genscript, HK limited; >85% purity) or Purified Protein Derivate (PPD, 10µg/mL, SSI) for 12h (Brefeldin A added after 7h) prior to fixation and cryopreservation (Kagina et al., 2015). ICS was done in one-step after a 10 min incubation (dark at room temperature) with 200µL Perm/Wash buffer (cat.no 554723, BD) followed by a 5 min centrifugation at 450-500 rcf and decantation: The 50 µL color mix per sample well included titrated volumes of CD3 AmCyan (clone SK7/Leu-4/cat.no 339186, BD, at 0.3125 µL), CD4 APC-H7 (SK3/Leu-3a/cat.no 641398, BD, at 0.625 µL), CD8 AlexaFluor488 (clone SK1/cat.no 344716, Biolegend, at 0.625 µL), IFNγ BV421 (clone B27/cat.no 562988, BD Horizon, at 0.625 µL), TNFα APC (clone Mab11/cat.no 551384, BD Pharmingen, at 5.0 µL), IL2 PE (MQ1-17H12/cat.no 559334, BD Pharmingen, at 5.0 µL). The BD FACS Canto II flow cytometry with BD FACS Diva Software was applied, whereas data analysis was performed with the FlowJo Software version 10.4.1 (TreeStarInc, Ashland, OR, USA). The immunological readout $Cytokine^+$ CD4 or CD8 T cells denoted producers of any of the measured cytokines. Other subpopulations are specified according to their cytokine production. All samples with an invalid positive control were excluded from analyses. Invalid positive control was defined as: Invalid if SEB induced tot IFNγ response by CD4 is < 4 Medians of the tot IFNγ response by CD4 T cells of the unstimulated controls of all participants in the study at the respective measurement time point

Quantification of anti-H56 IgG in serum
To measure the anti-H56 IgG in human serum an indirect ELISA was developed at SSI. An in-house established human reference serum pool, assigned 200 ELISA units/ml (EU/ml) was used for assay optimization and calibration of unknown sera. NUNC MaxiSorb plates was coated with 50 µl of H56 (Batch DS-026-17, SSI), diluted to 0.2 µg/ml in Carbonate buffer pH 9.6 and incubated 2 hours at room temperature or overnight at 2-8°C. The Reference serum and test samples was in 2-fold serial dilution in parallel. For detection the plate was added HRP labelled HRP labelled Rabbit anti-human IgG (DAKO; P0214 / Cat. No. P021402-02), OPD substrate was added and the reaction was stopped with 1M H_2SO_4. OD (492 nm) was read using an ELISA reader. A reference line approach on log-log transformed data was used to calculate the concentration of anti-H56 IgG antibodies, with the reference serum pool as calibrator.

Statistical methods – detailed description

The primary outcomes on safety were performed on the safety analysis set (SAS) (described in section statistical methods in article). The secondary outcomes of immunogenicity were assessed on the full analysis set (FAS). To acknowledge the possible impact of poor compliance to allocated treatment on immunogenicity, we calculated compliance for each group based on a priori definitions. For all arms, a participant was considered compliant if no corticosteroids/additional NSAIDs during the last seven days prior to sampling were taken. In addition, if allocated to etoricoxib, ≥80% of prescribed daily doses had to be taken during 1) the day 0-140 interval, and 2) the last 7 days prior to sampling. If allocated to H56:IC31-vaccine, ≥1 H56:IC31-vaccine were administered. The number (and percentage) of patients that were compliant/with major treatment are presented but no per protocol analyses were performed.

Primay endpoint: safety

The primary outcome is the occurrence of adverse events (AEs), serious adverse events (SAEs), and suspected unexpected serious adverse reactions (SUSARs) for the following time intervals:

a. Day 0 to day 84 (or date of first vaccination if applicable).

b. Day 85 (or date of first vaccination if applicable) to day 154 (or 14 days after last vaccination/last Arcoxia treatment, if applicable).

c. Day 155 (or 15 days after last vaccination/last Arcoxia treatment, if applicable) to day 238.

If the second vaccination is missing, day 154 is defined as 70 days after the date of day 84.

AEs are defined as any unintended sign, symptom or disease in a participant receiving the treatment and which does not necessarily have to be causally related with the treatment. The AEs will be coded using the Medical Dictionary for Regulatory Activities (MedDRA 22).

SAEs are defined as a medical occurrence that results in death, is life-threatening, requires in-patient hospitalisation or prolongation of the existing hospitalisation, results in disabilities, is a congenital abnormality of birth defect, or requires medical intervention to prevent the outcomes mentioned above. If there is a doubt about an AE being serious or not, it is considered serious.

SUSARs are defined as SAEs that are unexpected as defined in the study protocol (V6_121016), Section 8.1 and 8.2, pages 32-34 and possibly related to the study treatment.

We calculated the numbers and proportions of participants in the SAS that experienced an AE, SAE, and SUSAR by study arm and above defined three time intervals. We also calculated the number and proportions of participants with 1, 2, or ≥3 AEs, SAEs, and SUSARs; the number and proportion of participants with any treatment related AE and SAE; and, the number and proportion of participants with an AE/SAE leading to study drug discontinuation in each study arm and above defined three time intervals. No formal statistical testing of differences between the study arms was performed. Further, we described the total number of AEs, SAEs, SUSARs, treatment related AEs and SAEs, and AEs/SAEs leading to study drug discontinuation independent of number of participants. In addition, we described the absolute, number of participants with and proportion of participants with AE, SAE, SUSAR, severe AEs, and AEs leading to study drug discontinuation by system organ class (SOC) and preferred term (PT), without calculating any p-values.

Secondary endpoints: immunogenicity read-outs

We used descriptive statistics (medians and IQR) to describe all pre-defined immunogenicity readouts at each study time point. In the IgG assay, we also described the number and proportion of vaccine seroconverters in each group defined as an increase in H56 IgG-responses of >2-fold from day 84 to any time point after.

Immunogenicity read-outs of primary priority for Hypothesis 1 were responses to treatment defined as the change from day 0 to day 84 in the defined outcomes (**Table 3**). The outcomes were analyzed using median regression with treatment as only independent explanatory factor. The treatment difference of the combination of both groups allocated to etoricoxib (etoricoxib-group and etoricoxib+H56:IC31-group) and the combination of controls and the H56-IC31-group (vaccine not administered until day 84) were calculated as estimated difference in median response with 95% confidence interval based on 1000 bootstrap replications and corresponding two-sided p-value. For Hypothesis 2 and Hypothesis 3, immunogenicity read-outs of primary priority were responses to vaccine treatment defined as the change from day 84 to day 154 (**Table 4 and 5**). The seed for the bootstrap replications was set to 20200316 (pre-defined as the date of the final statistical analysis plan (Version 1.0).

Exploratory post-hoc analyses were performed for immunogenicity readouts predefined as secondary and tertiary priority from day 84 to day 154 using the same statistical analyses as for the primary priority read-outs described above.

Data were collected in the eCRF Viedoc™ version 4.66.7823.20827 and Microsoft Excel 2016. Statistical analyses were performed in STATA version 16 (StataCorp) and figures drawn in GraphPad Prism version 7.02.

Etoricoxib concentration

The analysis of plasma concentrations of Etoricoxib in human plasma has been performed using LC-MS/MS and samples were analyzed by Vitas AS, Oslo. 300 µL of human plasma was precipitated with 900 µL isopropanol containing deuterium labeled etoricoxib internal standard. After thorough mixing (10 min) and centrifugation (10 min, 4000 g at 10°C), an aliquot of 5 µL was injected from the supernatant into the High Performance Liquid Chromatography (HPLC) system. HPLC was performed with an Agilent 1260 liquid chromatograph (Agilent Technologies, Palo Alta, CA, USA) interfaced by atmospheric pressure chemical ionization (APCI) to a 6460 Agilent Technologies mass spectrometric detector. Etoricoxib was separated on a Kinetex C18, 100A, 2·6 µm column (Phenomenex, Torrance CA, USA). The column temperature was 50 °C. An eight-point calibration curve prepared in plasma covering concentrations from 0·17 to 4·0 µg/ml was used. The LOD for the method is 0·01 µg/ml and LOQ is 0·05 µg/ml.

Concentration etoricoxib (µg/ml)

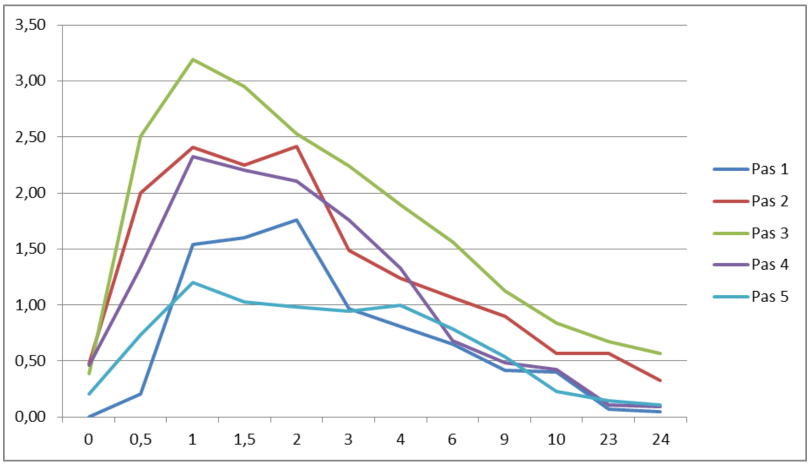

Hours after intake

Supplementary Figure 1: 24 hour concentration profile of etoricoxib after daily intake etoricoxib 120 mg x1. Plasma taken from patients (N=5) randomized to study group with etoricoxib alone. Measurements taken approximately 7 days after start of etoricoxib treatment to ensure steady state.

Supplementary Table 6: etoricoxib concentrations

sample-ID	Day 14	Day 84
1	1·12	1·91
2	0·72	0·38
3	2·42	3·05
4	1·74	0·44
5	0·81	1·85
6	0·83	3·69
7	1·95	1·05
8	0·12	0·19
9	0·41	0·90
10	0·84	0·44
11	1·02	1·58
12	0·82	0·29
13	2·50	1·60
14	0·60	0·56
15	1·08	1·12
16	0·48	0·46
17	0·81	3·10
18	2·58	2·55

Supplementary Table 6 showing concentration of etoricoxib at day 14 (d14) and day 84 (d84) in 18 patients from the study groups etoricoxib alone and combined etoricoxib + H56IC31 before first vaccination.

Supplementary Table 7. **Baseline characteristics by study arm for the Full analysis Set (FAS), N=40**

	Etoricoxib (N =10)	H56:IC31(N = 12)	Controls(N = 10)	Etoricoxib + H56:IC31 (N = 8)
**Demography**				
Sex (Male)	5 (50%)	8 (67%)	4 (40%)	4 (50%)
Age (years), Median (Q1, Q3)	31·0 (23·0, 35·0)	24·0 (20·5, 26·5)	28·0 (23·0, 34·0)	28·0 (24·5, 36·0)
Ethnicity				
Asian	2 (20%)	2 (17%)	4 (40%)	3 (38%)
Black	5 (50%)	7 (58%)	6 (60%)	3 (38%)
Caucasian	1 (10%)	3 (25%)	0 (0%)	2 (25%)
Other	2 (20%)	0 (0%)	0 (0%)	0 (0%)
**TB related medical history**				
BCG scar				
Yes	2 (20%)	5 (42%)	4 (40%)	6 (75%)
No	7 (70%)	6 (50%)	6 (60%)	1 (13%)
Unknown	1 (10%)	1 (8%)	0 (0%)	1 (13%)
Previous TB treatment	0 (0%)	1 (8%)	2 (20%)	1 (13%)
**Co-morbidity and Risk factors**				
Allergy	2 (20%)	1 (8%)	3 (30%)	2 (25%)
Previous gastric ulcer/gastroenteric bleeding	0 (0%)	1 (8%)	1 (10%)	0 (0%)
Liver disease/failure	1 (10%)	0 (0%)	1 (10%)	0 (0%)
Pulmonary disease	0 (0%)	0 (0%)	0 (0%)	0 (0%)
Other medical condition*	2 (20%)	3 (25%)	3 (30%)	4 (50%)
Smoking status	2 (20%)	0 (0%)	0 (0%)	1 (13%)
Alcohol use >3 units/week	2 (20%)	1 (8%)	1 (10%)	3 (38%)
Drug abuse	0 (0%)	1 (8%)	0 (0%)	0 (0%)
**Clinical Classification and Radiology**				
TB diagnosis	8 (80%)	10 (83%)	7 (70%)	4 (50%)
Pulmonary	1 (10%)	2 (17%)	0 (0%)	1 (13%)
Pulmonary/extrapulmonary	1 (10%)	0 (0%)	3 (30%)	3 (38%)
Extrapulmonary				
Lymph nodes / lymphadenitis	1	1	3	4
Bones, joint and soft tissue	0	0	0	1
Other	1	1	0	0
Radiology				
Pulmonary cases				
Infiltrate	7	11	3	3
Cavity	5	3	1	0
Pleural fluid	1	1	0	0
With hilary/mediastinal changes	0	2	0	0
**Clinical Features**				
Symptoms (cough, chest pain, night sweat)				
No symptoms	2 (20%)	2 (17%)	7 (70%)	5 (63%)
1 symptom	1 (10%)	1 (8%)	1 (10%)	2 (25%)
≥2 symptoms	7 (70%)	9 (75%)	2 (20%)	1 (13%)
BMI** (kg/m2) Median (Q1, Q3)	20·4 (18·9, 21·6)	20·8 (19·1, 21·7)	21·7 (18·9, 24·9)	21·6 (18·1, 23·8)
Body temperature (°C) Median (Q1, Q3)	36·6 (36·5, 37·1)	36·3 (36·1, 36·8)	36·6 (36·4, 37·0)	36·5 (36·2, 36·6)
**Laboratory data**				
Haemoglobin (g/dl)				
Median (Q1, Q3)	13·3 (12·6, 14·9)	13·3 (12·3, 14·9)	13·9 (13·4, 15·1)	14·0 (13·3, 15·0)
N (% Non-missing)	9 (90%)	10 (83%)	8 (80%)	8 (100%)
Sedimentation rate (mm)				
Median (Q1, Q3)	34·0 (13·0, 76·0)	20·0 (11·0, 52·0)	13·0 (5·0, 35·0)	26·0 (15·0, 41·0)
N (% Non-missing)	9 (90%)	10 (83%)	9 (90%)	7 (88%)
C-reactive protein (mg/L)				
Median (Q1, Q3)	19·6 (0·6, 52·2)	7·2 (0·7, 36·5)	0·7 (0·0, 6·4)	2·8 (0·9, 7·8)
N (% Non-missing)	10 (100%)	11 (92%)	9 (90%)	7 (88%)
Quantiferon result				
Negative	0 (0%)	0 (0%)	1 (10%)	0 (0%)
Positive	9 (90%)	10 (91%)	8 (80%)	8 (100%)
Indeterminate	1 (10%)	1 (9%)	1 (10%)	0 (0%)
Median IU/ml (Q1, Q3)	4·0 (3·7, 7·2)	4·8 (2·0, 8·0)	6·4 (3·9, 11·3)	12·6 (3·6, 15·4)
N (% Non-missing)	9 (90%)	10 (83%)	8 (80%)	8 (100%)

Unless indicated in table, no values were missing. *No participants had diabetes. **BMI: Body Mass index

Supplementary Table 8. Follow-up time, study discontinuation and adherence

Follow-up time and study discontinuation all arms - Safety Analysis Set* (N=47)

	Etoricoxib (N = 13)	H56:IC31 (N = 12)	Controls (N = 12)	Etoricoxib + H56:IC31 (N = 10)
**Follow-up time in days**				
Mean (SD)	200·4 (83·0)	227·3 (44·4)	214·0 (84·1)	199·8 (93·6)
Min, Max	11·0, 265·0	88·0, 250·0	50·0, 330·0	20·0, 285·0
**Did the subject complete the study**				
Yes	9 (69%)	10 (83%)	9 (75%)	6 (60%)
No	4 (31%)	2 (17%)	3 (25%)	4 (40%)
**Reasons for study discontinuation**				
Voluntary discontinuation by the subject	0 (0%)	1 (50%)	0 (0%)	0 (0%)
Safety reason as judged by the Principal Investigator	1 (25%)	0 (0%)	0 (0%)	2 (50%)
Major protocol deviation	1 (25%)	0 (0%)	0 (0%)	0 (0%)
Pregnancy during follow-up	0 (0%)	0 (0%)	2 (67%)	1 (25%)
TB disease progression	1 (25%)	0 (0%)	0 (0%)	0 (0%)
Autoimmune disease or immunosuppressive therapy during follow-up	0 (0%)	0 (0%)	1 (33%)	0 (0%)
Moved abroad	1 (25%)	1 (50%)	0 (0%)	1 (25%)

*Safety analysis set defined as patients that received at least one dose of etoricoxib and/or one dose of H56:IC31

Follow-up time and study discontinuation all arms - Full Analysis set* (N=40)

	Etoricoxib (N = 10)	H56:IC31 (N = 12)	Controls (N = 10)	Etoricoxib + H56:IC31 (N = 8)
**Follow-up time in days**				
Mean (SD)	242·7 (12·7)	227·3 (44·4)	246·7 (39·0)	241·4 (36·6)
Min, Max	220·0, 265·0	88·0, 250·0	168·0, 330·0	180·0, 285·0
**Did the subject complete the study**				
Yes	9 (90%)	10 (83%)	9 (90%)	6 (75%)
No	1 (10%)	2 (17%)	1 (10%)	2 (25%)
**Reasons for study discontinuation**				
Voluntary discontinuation by the subject	0 (0%)	1 (50%)	0 (0%)	0 (0%)
Pregnancy during follow-up	0 (0%)	0 (0%)	1 (100%)	1 (50%)
Moved abroad	1 (100%)	1 (50%)	0 (0%)	1 (50%)

*Full analysis set (FAS) defined as randomized patients with at least one valid immunogenicity measurement

Adherence to Etoricoxib - Safety Analysis Set* (N=13).

	Total<br>(N = 13)
**Proportion of tablets taken from the number expected**	
Mean (SD)	96·0 (8·8)
Median (Q1, Q3)	99·3 (98·7, 100·0)
Min, Max	73·5, 102·9
**Proportion of tablets taken from the number expected in two categories**	
<80% of expected tablets	2 (15%)
≥80% of expected tablets	11 (85%)

Adherence to H56:IC31 - Safety Analysis Set* (N=12)

	Total<br>(N = 12)
**First vaccination given**	
No	1 (8%)
Yes	11 (92%)
**Second vaccination given**	
No	2 (17%)
Yes	10 (83%)

Adherence to Etoricoxib + H56:IC31 - Safety Analysis Set* (N=10)

	Total<br>(N = 10)
**Proportion of tablets taken from the number expected**	
Mean (SD)	98·3 (2·5)
Median (Q1, Q3)	100·0 (96·4, 100·0)
Min, Max	92·8, 100·0
**First vaccination given**	
No	2 (20%)
Yes	8 (80%)
**Second vaccination given**	
No	2 (20%)
Yes	8 (80%)
**Proportion of tablets taken from the number expected in two categories**	
≥80% of expected tablets	10 (100%)

*Safety analysis set defined as patients that received at least one dose of etoricoxib and/or one dose of H56:IC31

Adherence to Etoricoxib - Full Analysis Set* (N=10)

	Total<br>(N = 10)
**Proportion of tablets taken from the number expected**	
Mean (SD)	96·9 (8·4)
Median (Q1, Q3)	99·6 (99·3, 100·0)
Min, Max	73·5, 102·9
**Proportion of tablets taken from the number expected in two categories**	
<80% of expected tablets	1 (10%)
≥80% of expected tablets	9 (90%)

Adherence to H56:IC31 - Full Analysis Set* (N=12)

	Total<br>(N = 12)
**First vaccination given**	
No	1 (8%)
Yes	11 (92%)
**Second vaccination given**	
No	2 (17%)
Yes	10 (83%)

Adherence to Etoricoxib + H56:IC31 - Full Analysis Set* (N=8)

	Total (N = 8)
**Proportion of tablets taken from the number expected**	
Mean (SD)	97·9 (2·7)
Median (Q1, Q3)	98·9 (96·1, 100·0)
Min, Max	92·8, 100·0
**First vaccination given**	
Yes	8 (100%)
**Second vaccination given**	
Yes	8 (100%)
**Proportion of tablets taken from the number expected in two categories**	
≥80% of expected tablets	8 (100%)

*Full analysis set (FAS) defined as randomized patients with at least one valid immunogenicity measurement. To acknowledge the possible impact of poor compliance to allocated treatment on immunogenicity, we defined compliance for each group based on a priori definitions. A participant was considered compliant if no corticosteroids/additional NSAIDs during the last seven days prior to sampling were taken. If allocated to etoricoxib, ≥80% of prescribed daily doses had to be taken during 1) the day 0-140 interval, and 2) the last 7 days prior to sampling. If allocated to H56:IC31-vaccine, ≥1 H56:IC31-vaccine were administered.

Supplementary Table 9. Adverse events (AE) tables Day 0 to day 84. Safety Analysis Set* (N=47)

Summary of AEs

	etoricoxib N=13	H56:IC31 TB vaccine N=12	Control N=12	etoricoxib and H56:IC31 TB vaccine N=10
Any AE	11 (84·6%)	11 (91·7%)	11 (91·7%)	9 (90·0%)
Number of AEs	58	45	54	52
Number of SAEs	2	0	2	1
Number of SUSARs	1			
One AE	1 (7·7%)	4 (33·3%)	1 (8·3%)	1 (10·0%)
Two AEs	1 (7·7%)	2 (16·7%)		
Three or more AEs	9 (69·2%)	5 (41·7%)	10 (83·3%)	8 (80·0%)
Any SAE	2 (15·4%)		1 (8·3%)	1 (10·0%)
Any SUSAR	1 (7·7%)			
Any treatment related AE	10 (76·9%)	0	0	9 (90·0%)
Any treatment related SAE	2 (15·4%)			1 (10·0%)
Any AE leading to study drug discontinuation	2 (15·4%)		1 (8·3%)	4 (40·0%)
Number of AE leading to study drug discontinuation	2		1	7

AE by System Organ Class (SOC) and Preferred term (PT) ([no. of events] no of patients (% of patients)

System Organ Class	Preferred term	etoricoxib N=13	H56:IC31 TB vaccine N=12	Control N=12	etoricoxib and H56:IC31 TB vaccine N=10
Blood and lymphatic system disorders	Anaemia			[1] 1 (8·3%)	
	Leukopenia				[1] 1 (10·0%)
	Lymphadenopathy			[1] 1 (8·3%)	
	Thrombocytopenia				[1] 1 (10·0%)
Gastrointestinal disorders	Abdominal discomfort	[1] 1 (7·7%)			[1] 1 (10·0%)
	Abdominal pain	[1] 1 (7·7%)			[2] 2 (20·0%)
	Abdominal pain upper	[1] 1 (7·7%)	[1] 1 (8·3%)	[2] 2 (16·7%)	
	Diarrhoea	[1] 1 (7·7%)			
	Dry mouth	[1] 1 (7·7%)	[1] 1 (8·3%)		
	Dyspepsia	[1] 1 (7·7%)	[3] 2 (16·7%)	[2] 2 (16·7%)	
	Gastritis	[1] 1 (7·7%)	[1] 1 (8·3%)		
	Haemorrhoids	[1] 1 (7·7%)			
	Nausea	[7] 6 (46·2%)	[4] 4 (33·3%)	[3] 3 (25·0%)	[6] 5 (50·0%)
	Vomiting		[1] 1 (8·3%)		[1] 1 (10·0%)
General disorders and administration site conditions	Chest pain	[1] 1 (7·7%)	[3] 3 (25·0%)		
	Disease progression	[1] 1 (7·7%)			
	Fatigue	[9] 8 (61·5%)	[4] 4 (33·3%)	[6] 6 (50·0%)	[3] 3 (30·0%)
	Pyrexia				[1] 1 (10·0%)
	Vaccination site pain				[2] 2 (20·0%)
Immune system disorders	Immune reconstitution inflammatory syndrome				[1] 1 (10·0%)
Infections and infestations	Lymph node abscess				[1] 1 (10·0%)
	Nasopharyngitis	[1] 1 (7·7%)			
	Otitis externa			[1] 1 (8·3%)	
	Respiratory tract infection	[1] 1 (7·7%)			
	Tooth infection		[1] 1 (8·3%)		
Injury, poisoning and procedural complications	Contusion				[1] 1 (10·0%)
Investigations	Blood creatine phosphokinase increased		[2] 1 (8·3%)		
	Cardiac murmur			[1] 1 (8·3%)	
	Hepatic enzyme increased	[8] 5 (38·5%)	[3] 3 (25·0%)	[3] 2 (16·7%)	[4] 3 (30·0%)
	Weight decreased			[2] 2 (16·7%)	
Metabolism and nutrition disorders	Decreased appetite			[4] 4 (33·3%)	[1] 1 (10·0%)
Musculoskeletal connective tissue disorders	Arthralgia	[3] 3 (23·1%)	[3] 3 (25·0%)	[6] 6 (50·0%)	[2] 2 (20·0%)
	Myalgia	[3] 3 (23·1%)	[4] 2 (16·7%)	[2] 2 (16·7%)	[2] 2 (20·0%)

	Pain in extremity				[1] 1 (10·0%)
Nervous system disorders	Cerebral ischaemia			[1] 1 (8·3%)	
	Dizziness	[3] 3 (23·1%)		[1] 1 (8·3%)	[2] 2 (20·0%)
	Dysgeusia	[1] 1 (7·7%)		[1] 1 (8·3%)	[1] 1 (10·0%)
	Headache	[6] 6 (46·2%)	[5] 5 (41·7%)	[2] 2 (16·7%)	[2] 1 (10·0%)
	Muscle contractions involuntary			[1] 1 (8·3%)	
	Paraesthesia		[1] 1 (8·3%)		
	Presyncope	[1] 1 (7·7%)			
Pregnancy, puerperium and perinatal conditions	Pregnancy			[1] 1 (8·3%)	
Psychiatric disorders	Anxiety				[1] 1 (10·0%)
Renal and urinary disorders	Pollakiuria				[1] 1 (10·0%)
Reproductive system and breast disorders	Testicular pain				[1] 1 (10·0%)
Respiratory, thoracic and mediastinal disorders	Cough		[1] 1 (8·3%)	[2] 1 (8·3%)	[2] 2 (20·0%)
	Dyspnoea		[1] 1 (8·3%)		[1] 1 (10·0%)
	Haemoptysis			[1] 1 (8·3%)	
Skin and subcutaneous tissue disorders	Dermatitis acneiform		[1] 1 (8·3%)	[1] 1 (8·3%)	
	Eczema			[2] 2 (16·7%)	
	Hand dermatitis				[1] 1 (10·0%)
	Hyperhidrosis		[1] 1 (8·3%)		[1] 1 (10·0%)
	Night sweats	[1] 1 (7·7%)		[2] 2 (16·7%)	[1] 1 (10·0%)
	Pruritus	[2] 2 (15·4%)	[1] 1 (8·3%)	[2] 2 (16·7%)	[4] 3 (30·0%)
	Pruritus generalised		[1] 1 (8·3%)		
	Rash	[1] 1 (7·7%)	[1] 1 (8·3%)	[3] 3 (25·0%)	[1] 1 (10·0%)
	Rash generalised				[1] 1 (10·0%)
	Urticaria	[1] 1 (7·7%)			
Vascular disorders	Diastolic hypertension		[1] 1 (8·3%)		
	Haematoma				[1] 1 (10·0%)

Severe adverse events by System Organ Class (SOC) and Preferred term (PT ([no. of events] no. of patients (pct of patients))

System Organ Class	Preferred term	etoricoxib N=13	H56:IC31 TB vaccine N=12	Control N=12	etoricoxib and H56:IC31 TB vaccine N=10
Gastrointestinal disorders	Abdominal pain				[1] 1 (10·0%)
	Abdominal pain upper	[1] 1 (7·7%)			
General disorders and administration site conditions	Fatigue	[1] 1 (7·7%)		[1] 1 (8·3%)	
Nervous system disorders	Cerebral ischaemia			[1] 1 (8·3%)	
Skin and subcutaneous tissue disorders	Rash generalised				[1] 1 (10·0%)

Serious adverse events by SOC and PT ([no. of events] no. of patients (pct of patients))

System Organ Class	Preferred term	etoricoxib N=13	H56:IC31 TB vaccine N=12	Control N=12	etoricoxib and H56:IC31 TB vaccine N=10
General disorders and administration site conditions	Disease progression	[1] 1 (7·7%)			
Nervous system disorders	Cerebral ischaemia			[1] 1 (8·3%)	
Respiratory, thoracic and mediastinal disorders	Haemoptysis			[1] 1 (8·3%)	
Skin and subcutaneous tissue disorders	Rash	[1] 1 (7·7%)			
	Rash generalised				[1] 1 (10·0%)

Suspected unexpected adverse reactions by SOC and PT ([no. of events] no. of patients (pct of patients))

System Organ Class	Preferred term	etoricoxib N=13	H56:IC31 TB vaccine N=12	Control N=12	etoricoxib and H56:IC31 TB vaccine N=10
Skin and subcutaneous tissue disorders	Rash	[1] 1 (7·7%)			

Adverse events leading to study drug discontinuation by SOC and PT ([no. of events] no. of patients (pct of patients))

System Organ Class	Preferred term	etoricoxib N=13	H56:IC31 TB vaccine N=12	Control N=12	etoricoxib and H56:IC31 TB vaccine N=10
Gastrointestinal disorders	Abdominal pain upper	[1] 1 (7·7%)			
	Gastritis	[1] 1 (7·7%)			
	Nausea	[1] 1 (7·7%)			
	Vomiting				[1] 1 (10·0%)
General disorders and administration site conditions	Fatigue	[1] 1 (7·7%)			
Investigations	Hepatic enzyme increased	[3] 2 (15·4%)			
Nervous system disorders	Dizziness				[1] 1 (10·0%)
Skin and subcutaneous tissue disorders	Hyperhidrosis				[1] 1 (10·0%)

Most frequent AEs (4 or more occurences) by PT ([no. of events] no. of patients (pct of patients))

Preferred term	etoricoxib N=13	H56:IC31 TB vaccine N=12	Control N=12	etoricoxib and H56:IC31 TB vaccine N=10
Fatigue	[9] 8 (61·5%)	[4] 4 (33·3%)	[6] 6 (50·0%)	[3] 3 (30·0%)
Nausea	[7] 6 (46·2%)	[4] 4 (33·3%)	[3] 3 (25·0%)	[6] 5 (50·0%)
Arthralgia	[3] 3 (23·1%)	[3] 3 (25·0%)	[6] 6 (50·0%)	[2] 2 (20·0%)
Headache	[6] 6 (46·2%)	[5] 5 (41·7%)	[2] 2 (16·7%)	[2] 1 (10·0%)
Hepatic enzyme increased	[8] 5 (38·5%)	[3] 3 (25·0%)	[3] 2 (16·7%)	[4] 3 (30·0%)
Myalgia	[3] 3 (23·1%)	[4] 2 (16·7%)	[2] 2 (16·7%)	[2] 2 (20·0%)
Pruritus	[2] 2 (15·4%)	[1] 1 (8·3%)	[2] 2 (16·7%)	[4] 3 (30·0%)
Rash	[1] 1 (7·7%)	[1] 1 (8·3%)	[3] 3 (25·0%)	[1] 1 (10·0%)
Dizziness	[3] 3 (23·1%)		[1] 1 (8·3%)	[2] 2 (20·0%)
Dyspepsia	[1] 1 (7·7%)	[3] 2 (16·7%)	[2] 2 (16·7%)	
Decreased appetite			[4] 4 (33·3%)	[1] 1 (10·0%)
Cough		[1] 1 (8·3%)	[2] 1 (8·3%)	[2] 2 (20·0%)
Night sweats	[1] 1 (7·7%)		[2] 2 (16·7%)	[1] 1 (10·0%)
Chest pain	[1] 1 (7·7%)	[3] 3 (25·0%)		
Abdominal pain upper	[1] 1 (7·7%)	[1] 1 (8·3%)	[2] 2 (16·7%)	

Supplementary Table 10. Adverse events tables Day 85 to day 154 Safety Analysis Set* (N=40)

Summary of AEs

	etoricoxib N=11	H56:IC31 TB vaccine N=11	Control N=10	etoricoxib and H56:IC31 TB vaccine N=8
Any AE	7 (63·6%)	9 (81·8%)	5 (50·0%)	5 (62·5%)
Number of AEs	20	24	15	15
Number of SAEs	0	0	0	0
Number of SUSARs	0	0	0	0
One AE	1 (9·1%)	1 (9·1%)	2 (20·0%)	3 (37·5%)
Two AEs	2 (18·2%)	4 (36·4%)		
Three or more AEs	4 (36·4%)	4 (36·4%)	3 (30·0%)	2 (25·0%)
Any treatment related AE	4 (36·4%)	6 (54·5%)		3 (37·5%)
Number of AE leading to study drug discontinuation	0	1 (9·1%)*		0

* 1 patient registered with study drug interruption, but due to not receiving vaccine dose nr 2, the AE was re-categorized to "AE leading to study drug discontinuation".

Adverse events by SOC and PT([no. of events] no of patients (% of patients))

System Organ Class	Preferred term	etoricoxib N=11	H56:IC31 TB vaccine N=11	Control N=10	etoricoxib and H56:IC31 TB vaccine N=8
Blood and lymphatic system disorders	Anaemia		[1] 1 (9·1%)		
	Lymphadenopathy		[1] 1 (9·1%)		
Gastrointestinal disorders	Abdominal pain	[1] 1 (9·1%)	[1] 1 (9·1%)	[1] 1 (10·0%)	[1] 1 (12·5%)
	Diarrhoea			[1] 1 (10·0%)	
	Lip ulceration				[1] 1 (12·5%)
	Nausea	[1] 1 (9·1%)	[1] 1 (9·1%)	[2] 1 (10·0%)	[1] 1 (12·5%)
	Toothache		[1] 1 (9·1%)		
	Vomiting			[1] 1 (10·0%)	
General disorders and administration site conditions	Chest pain	[1] 1 (9·1%)			
	Fatigue	[2] 2 (18·2%)	[3] 3 (27·3%)		[1] 1 (12·5%)
	Influenza like illness				[1] 1 (12·5%)
	Local reaction		[1] 1 (9·1%)		[1] 1 (12·5%)
	Vaccination site induration				[1] 1 (12·5%)
	Vaccination site pain		[1] 1 (9·1%)		[2] 2 (25·0%)
Infections and infestations	Chlamydial infection			[1] 1 (10·0%)	
	Cystitis	[1] 1 (9·1%)			
	Nasopharyngitis				[1] 1 (12·5%)
	Sinusitis	[1] 1 (9·1%)			
	Tinea pedis			[1] 1 (10·0%)	
Investigations	Blood creatinine increased			[1] 1 (10·0%)	
	Hepatic enzyme increased	[1] 1 (9·1%)	[2] 2 (18·2%)	[1] 1 (10·0%)	
	Transaminases increased			[1] 1 (10·0%)	
	Weight decreased	[2] 2 (18·2%)			
Musculoskeletal and connective tissue disorders	Arthralgia	[1] 1 (9·1%)	[4] 4 (36·4%)		[1] 1 (12·5%)
	Muscle haemorrhage		[1] 1 (9·1%)		
	Musculoskeletal pain	[1] 1 (9·1%)			
	Myalgia			[1] 1 (10·0%)	[1] 1 (12·5%)
	Tendonitis			[1] 1 (10·0%)	
Nervous system disorders	Dizziness				[1] 1 (12·5%)
	Headache	[1] 1 (9·1%)	[2] 2 (18·2%)		
	Paraesthesia		[1] 1 (9·1%)	[1] 1 (10·0%)	
Psychiatric disorders	Depression	[1] 1 (9·1%)			
Respiratory, thoracic and mediastinal disorders	Asthma	[1] 1 (9·1%)			
	Cough	[2] 2 (18·2%)	[1] 1 (9·1%)		
	Dyspnoea	[1] 1 (9·1%)			
	Rhinorrhoea	[1] 1 (9·1%)			
	Wheezing	[1] 1 (9·1%)			[2] 1 (12·5%)

Skin and subcutaneous tissue disorders	Dermatitis acneiform		[1] 1 (9·1%)		
	Night sweats			[1] 1 (10·0%)	
	Pruritus		[1] 1 (9·1%)		
	Rash		[1] 1 (9·1%)		
	Urticaria			[1] 1 (10·0%)	

Severe adverse events by SOC and PT ([no. of events] no. of patients (pct of patients))

System Organ Class	Preferred term	etoricoxib N=11	H56:IC31 TB vaccine N=11	Control N=10	etoricoxib and H56:IC31 TB vaccine N=8
Gastrointestinal disorders	Abdominal pain	[1] 1 (9·1%)			

Serious adverse events by SOC and PT ([no. of events] no. of patients (pct of patients))

System Organ Class	Preferred term	etoricoxib N=11	H56:IC31 TB vaccine N=11	Control N=10	etoricoxib and H56:IC31 TB vaccine N=8
No observations					

Suspected unexpected adverse reactions by SOC and PT ([no. of events] no. of patients (pct of patients))

System Organ Class	Preferred term	etoricoxib N=11	H56:IC31 TB vaccine N=11	Control N=10	etoricoxib and H56:IC31 TB vaccine N=8
No observations					

Adverse events leading to study drug discontinuation by SOC and PT ([no. of events] no. of patients (pct of patients))

System Organ Class	Preferred term	etoricoxib N=11	H56:IC31 TB vaccine N=11	Control N=10	etoricoxib and H56:IC31 TB vaccine N=8
Blood and lymphatic system disorders	Lymphadenopathy		[1] 1 (9·1%)		
Gastrointestinal disorders	Abdominal pain		[1] 1 (9·1%)		

Most frequent AEs (4 or more occurences) by PT ([no. of events] no. of patients (pct of patients))

Preferred term	etoricoxib N=11	H56:IC31 TB vaccine N=11	Control N=10	etoricoxib and H56:IC31 TB vaccine N=8
Arthralgia	[1] 1 (9·1%)	[4] 4 (36·4%)		[1] 1 (12·5%)
Fatigue	[2] 2 (18·2%)	[3] 3 (27·3%)		[1] 1 (12·5%)
Hepatic enzyme increased	[1] 1 (9·1%)	[2] 2 (18·2%)	[1] 1 (10·0%)	
Nausea	[1] 1 (9·1%)	[1] 1 (9·1%)	[2] 1 (10·0%)	[1] 1 (12·5%)
Abdominal pain	[1] 1 (9·1%)	[1] 1 (9·1%)	[1] 1 (10·0%)	[1] 1 (12·5%)

Supplementary Table 11. Adverse events tables Day 155 to day 238 Safety Analysis Set* (N=38)

Summary of AEs

	etoricoxib N=10	H56:IC31 TB vaccine N=11	Control N=9	etoricoxib and H56:IC31 TB vaccine N=8
Any AE	7 (70·0%)	8 (72·7%)	4 (44·4%)	3 (37·5%)
Number of AEs	12	14	7	8
Number of SAEs	1	0	0	0
Number of SUSARs	0	0	0	0
One AE	5 (50·0%)	5 (45·5%)	2 (22·2%)	1 (12·5%)
Two AEs		1 (9·1%)	1 (11·1%)	1 (12·5%)
Three or more AEs	2 (20·0%)	2 (18·2%)	1 (11·1%)	1 (12·5%)
Any SAE	1 (10·0%)			
Any treatment related AE	3 (30·0%)	3 (27·3%)		2 (25·0%)
Any treatment related SAE	1 (10·0%)	0	0	0
Number of AE leading to study drug discontinuation	0			0

Adverse events by SOC and PT([no. of events] no of patients (% of patients))

System Organ Class	Preferred term	etoricoxib N=10	H56:IC31 TB vaccine N=11	Control N=9	etoricoxib and H56:IC31 TB vaccine N=8
Blood and lymphatic system disorders	Lymphadenopathy				[3] 2 (25·0%)
	Neutropenia			[1] 1 (11·1%)	
	Thrombocytopenia		[1] 1 (9·1%)		
Eye disorders	Visual acuity reduced			[1] 1 (11·1%)	
Gastrointestinal disorders	Aphthous ulcer				[1] 1 (12·5%)
	Duodenitis	[1] 1 (10·0%)			
	Dyspepsia	[1] 1 (10·0%)			
	Nausea	[2] 2 (20·0%)			
	Vomiting		[1] 1 (9·1%)		
General disorders and administration site conditions	Chest pain		[2] 2 (18·2%)		
	Fatigue				[1] 1 (12·5%)
	Pyrexia	[1] 1 (10·0%)			
Infections and infestations	Nasopharyngitis		[1] 1 (9·1%)	[1] 1 (11·1%)	
	Viral infection		[1] 1 (9·1%)		
	Viral upper respiratory tract infection		[1] 1 (9·1%)		
Investigations	Blood creatine phosphokinase increased		[1] 1 (9·1%)		
	Hepatic enzyme increased		[1] 1 (9·1%)	[1] 1 (11·1%)	
	Oestradiol decreased			[1] 1 (11·1%)	
	Red blood cell sedimentation rate increased	[2] 2 (20·0%)	[1] 1 (9·1%)		
	Weight decreased				[1] 1 (12·5%)
Musculoskeletal and connective tissue disorders	Arthralgia		[1] 1 (9·1%)	[1] 1 (11·1%)	
	Rhabdomyolysis		[1] 1 (9·1%)		
Nervous system disorders	Headache	[1] 1 (10·0%)		[1] 1 (11·1%)	[1] 1 (12·5%)
	Paraesthesia	[1] 1 (10·0%)			
Reproductive system and breast disorders	Testicular pain		[1] 1 (9·1%)		
Respiratory, thoracic and mediastinal disorders	Lung infiltration	[1] 1 (10·0%)			
Skin and subcutaneous tissue disorders	Acne				[1] 1 (12·5%)
	Erythema nodosum		[1] 1 (9·1%)		
	Night sweats	[2] 1 (10·0%)			

Severe adverse events by SOC and PT ([no. of events] no. of patients (pct of patients))

System Organ Class	Preferred term	etoricoxib N=10	H56:IC31 TB vaccine N=11	Control N=9	etoricoxib and H56:IC31 TB vaccine N=8
Gastrointestinal disorders	Duodenitis	[1] 1 (10·0%)			
Investigations	Hepatic enzyme increased			[1] 1 (11·1%)	

Serious adverse events by SOC and PT ([no. of events] no. of patients (pct of patients))

System Organ Class	Preferred term	etoricoxib N=10	H56:IC31 TB vaccine N=11	Control N=9	etoricoxib and H56:IC31 TB vaccine N=8
Gastrointestinal disorders	Duodenitis	[1] 1 (10·0%)			

Suspected unexpected adverse reactions by SOC and PT ([no. of events] no. of patients (pct of patients))

System Organ Class	Preferred term	etoricoxib N=10	H56:IC31 TB vaccine N=11	Control N=9	etoricoxib and H56:IC31 TB vaccine N=8
No observations					

Adverse events leading to study drug discontinuation by SOC and PT ([no. of events] no. of patients (pct of patients))

System Organ Class	Preferred term	etoricoxib N=10	H56:IC31 TB vaccine N=11	Control N=9	etoricoxib and H56:IC31 TB vaccine N=8
No observations					

Most frequent AEs (4 or more occurences) by PT ([no. of events] no. of patients (pct of patients))

Preferred term	etoricoxib N=10	H56:IC31 TB vaccine N=11	Control N=9	etoricoxib and H56:IC31 TB vaccine N=8

Supplementary Figure 2

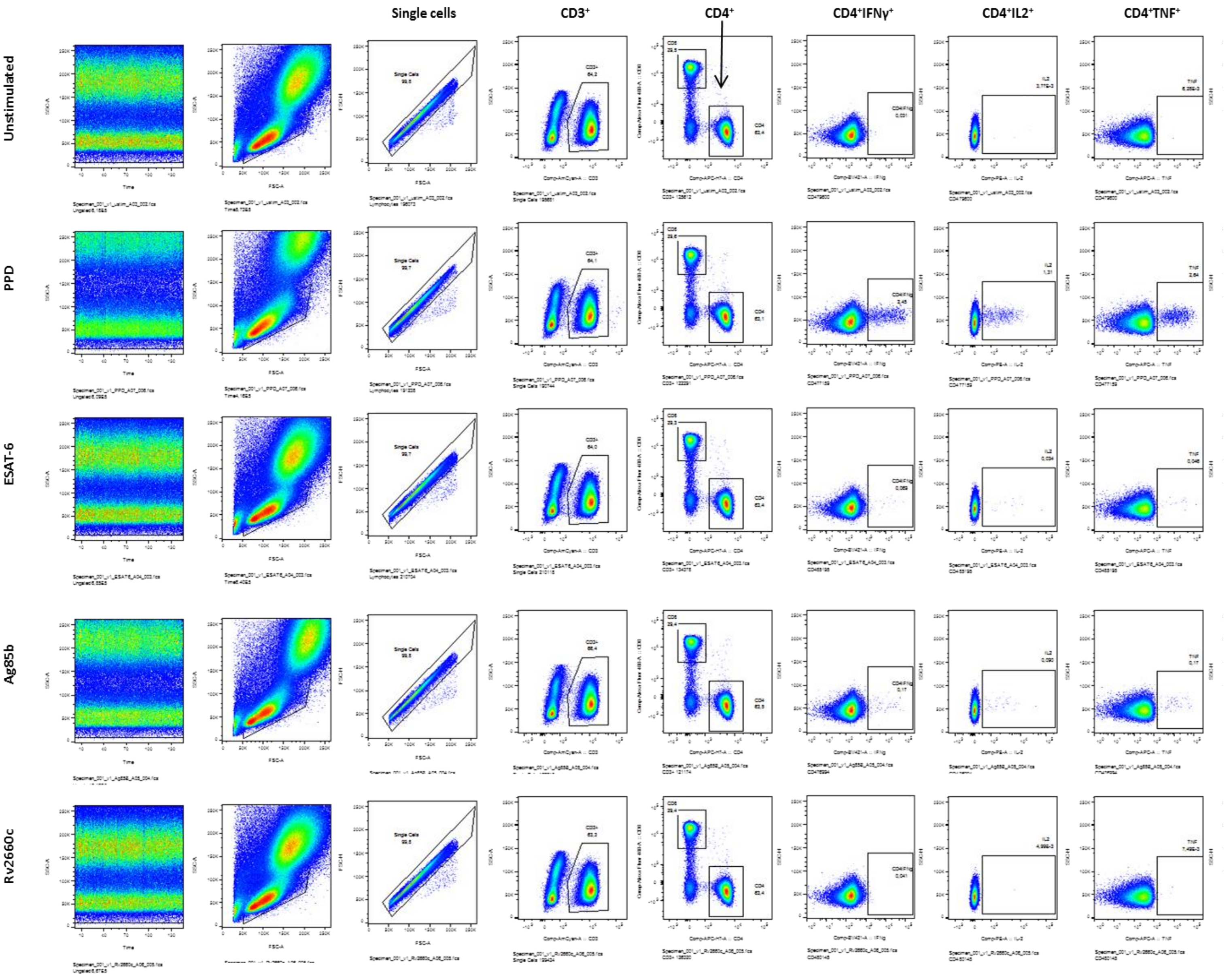

Supplementary Figure 2. Flow cytometry gating strategy for the identification of CD4$^+$ T cell subsets corresponding to Fig. 2 b and c, Fig. 3 e and f and Fig. 4 e and f in the manuscript. Representative dot plots from a single participant acquired following whole blood intracellular cytokine staining assay are shown for unstimulated samples and samples stimulated with Purified Protein Derivate (PPD), Early Secretory Antigenic Target 6 (ESAT-6), Antigen 85b (Ag85b) and Latency associated protein Rv2660c. We used a time gate to exclude events that showed inconsistent fluorescence during acquisition, followed by a wide lymphocyte gate in forward-scatter (FSC)/side-scatter (SSC) plots and singlet cells in FSC-area (FSC-A) and FSC-height (FSC-H). We then selected T-cells by CD3$^+$ surface expression (AmCyam) followed by separation of CD4$^+$ (APC-H7) and CD8$^+$ (Alexa Fluor 488) subsets by a quadrant gate. Included cytokines IFNγ (Brilliant Violet 421), IL2 (PE) and TNFα (APC) were analysed by Boolean gating. Individual single cytokine gates were set applying unstimulated controls.

Supplementary Figure 3

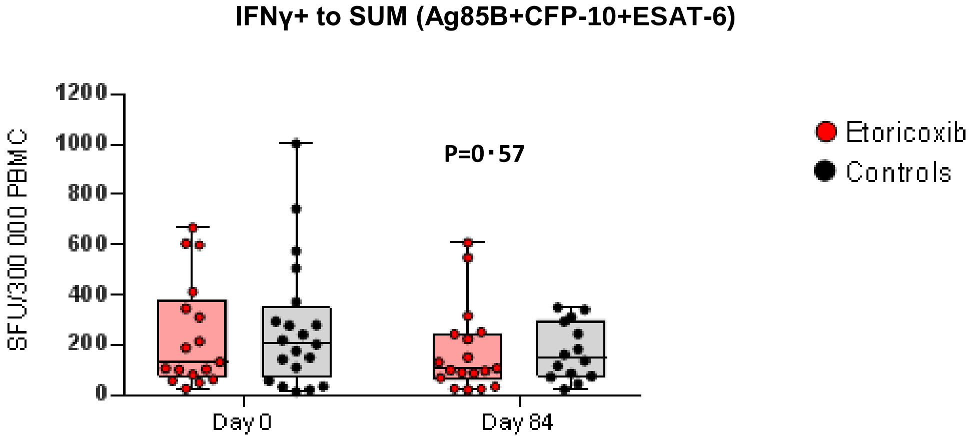

Supplementary Figure 3. Immunoregulatory effects of etoricoxib. Effect of etoricoxib administered during the first 84 days of standard TB treatment on Mtb-specific IFNγ^{+} T-cells (Hypothesis 1, table 3) in response to Ag85, ESAT-6 and CFP10 summed, in the etoricoxib group (red) and controls (grey) measured by Fluorospot at day 0 (n: 17/20) and day 84 (n: 18/14). Plots are shown with median, interquartile ranges (IQR) and minimum/maximum values. As the study was planned as an exploratory hypothesis generating phase I/II trial, all group comparisons, including p-values were regarded hypothesis supporting and not interpreted as confirmatory. Adjustments for multiple testing were not performed but a hierarchical order of immunogenicity outcomes defined *a priori* (Supplementary Data pp. 6-7) was judged adequate to assign importance/interpret the results of the respective statistical tests. Immunogenicity readouts of primary priority were analyzed using median regression with treatment as only independent explanatory factor.

.

Supplementary Figure 4

Cytokine+ responses to ESAT-6

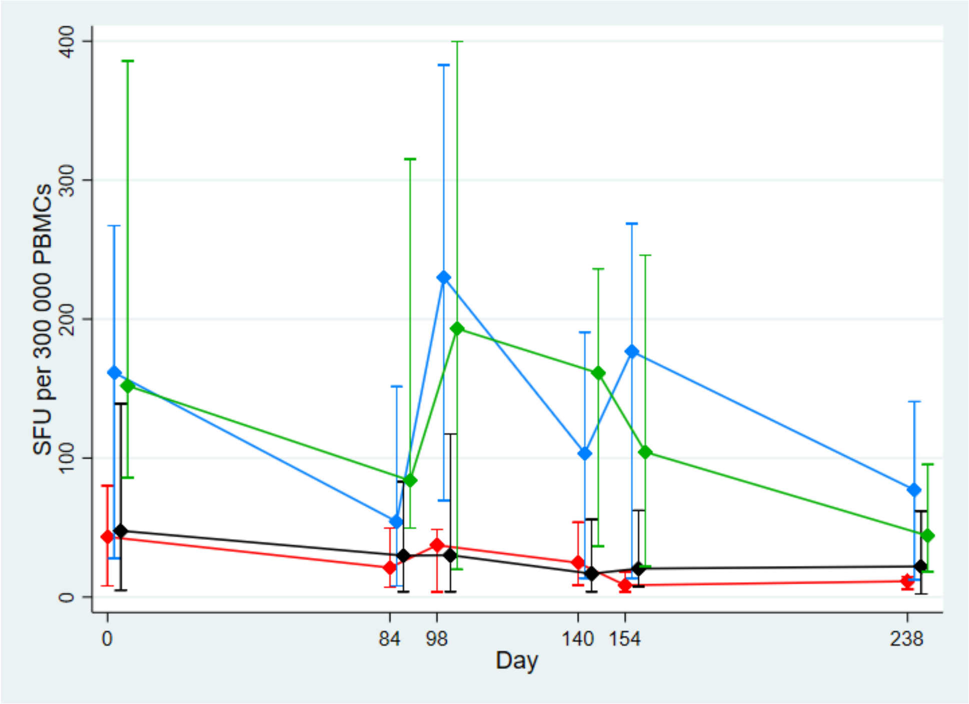

Cytokine+ responses to Ag85B

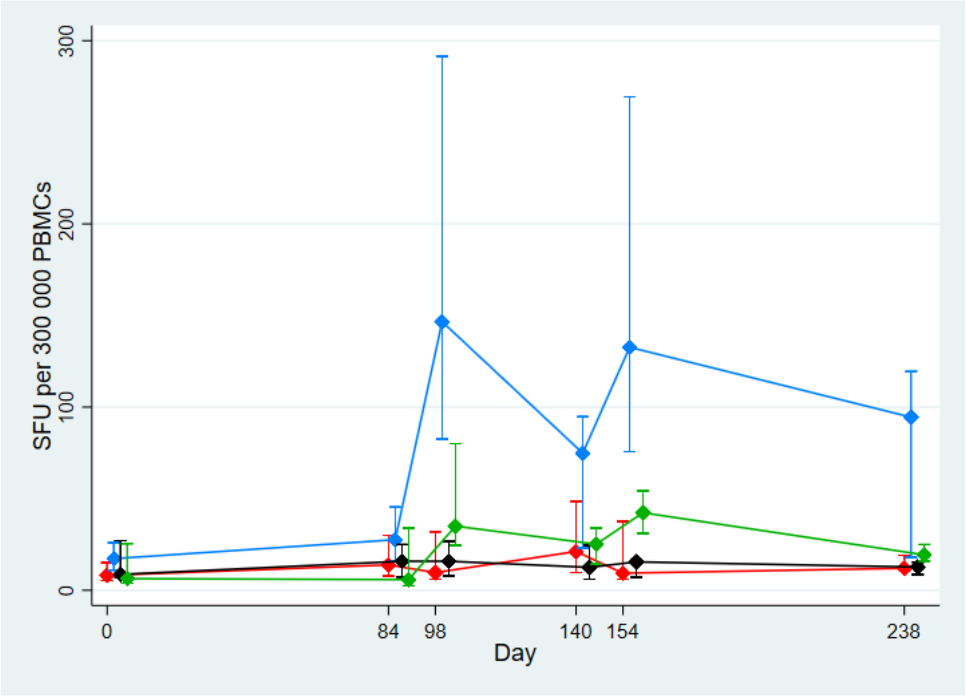

red=Etoricoxib, blue=H56:IC31, black=Controls, green=Etoricoxib+H56:IC31

Cytokine+ responses to CFP-10

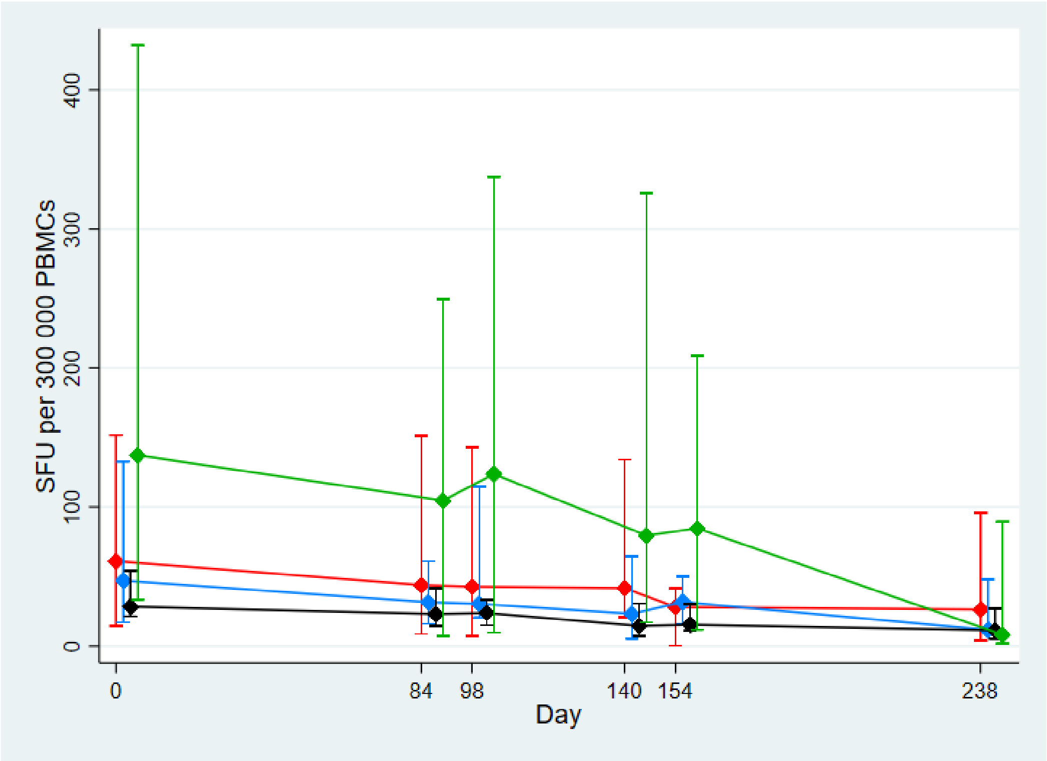

IFNγ+IL2+ responses to Ag85B, CFP-10 and ESAT-6

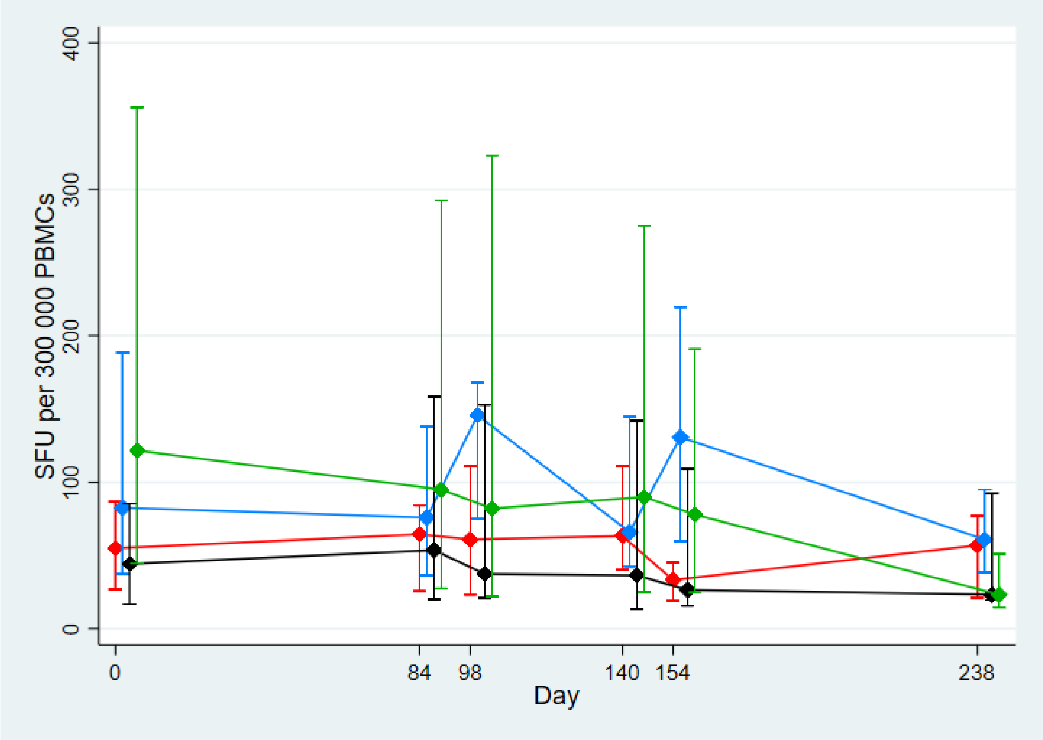

red=Etoricoxib, blue=H56:IC31, black=Controls, green=Etoricoxib+H56:IC31

IFNγ responses to Ag85B and ESAT-6

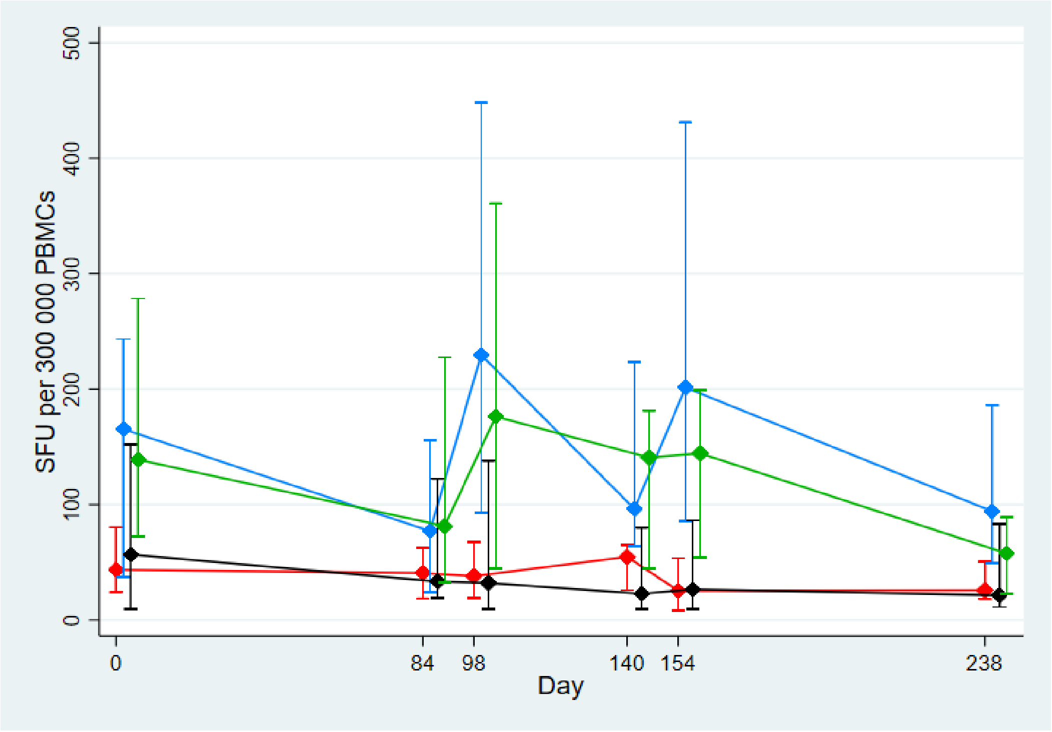

IFNγ+IL2+ responses to Ag85B and ESAT-6

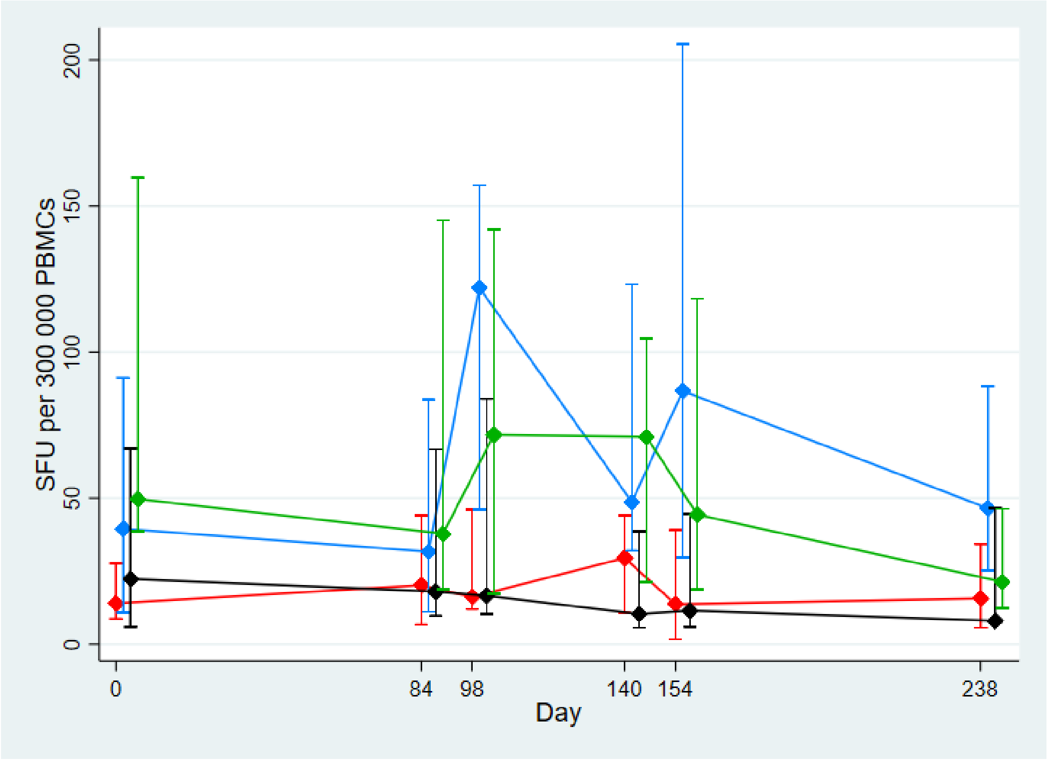

red=Etoricoxib, blue=H56:IC31, black=Controls, green=Etoricoxib+H56:IC31

Supplementary Figure 4. Cytokine responses for all study groups at all time points. Descriptive line plots of different cytokine responses measured by Fluorospot at day 0 to day 238 with median and IQR for all study groups; etoricoxib-group (red; n=10), H56:IC31-group (blue, n=12), controls (black, n=10) and etoricoxib+H56:IC31 group (green, n=8) are depicted for Cytokine+ T-cells to individual *Mtb*

peptides (ESAT-6, Ag85B and CFP-10) (first 3 panels), IFNγ+IL2+ T-cells in response to ESAT-6+Ag85B+CFP-10 (fourth panel) and ESAT-6+Ag85B (sixth panel) and finally IFNγ+ T-cells in response ESAT-6+Ag85B.

Supplementary Figure 5

a

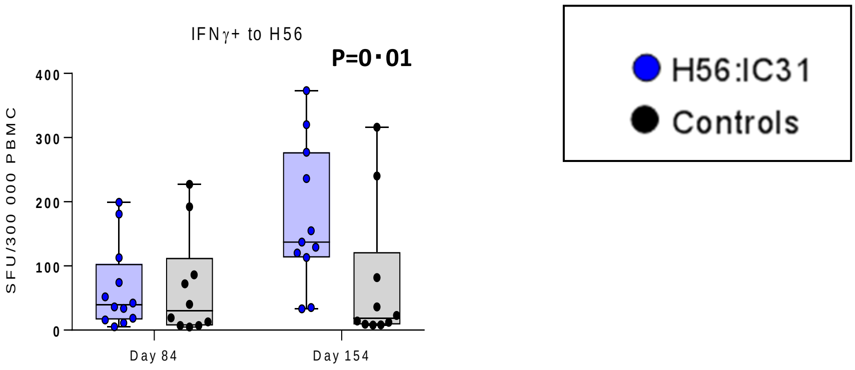

b

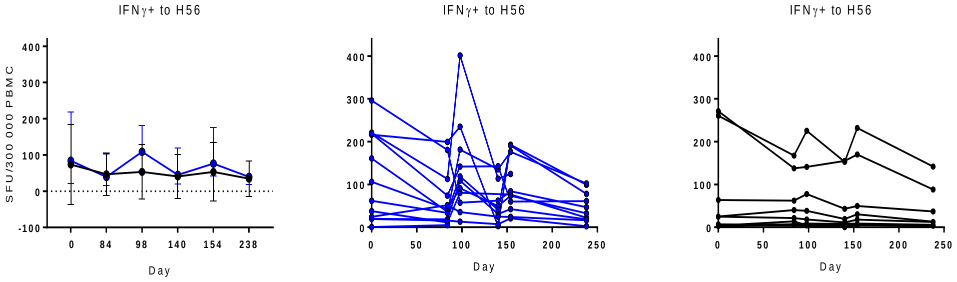

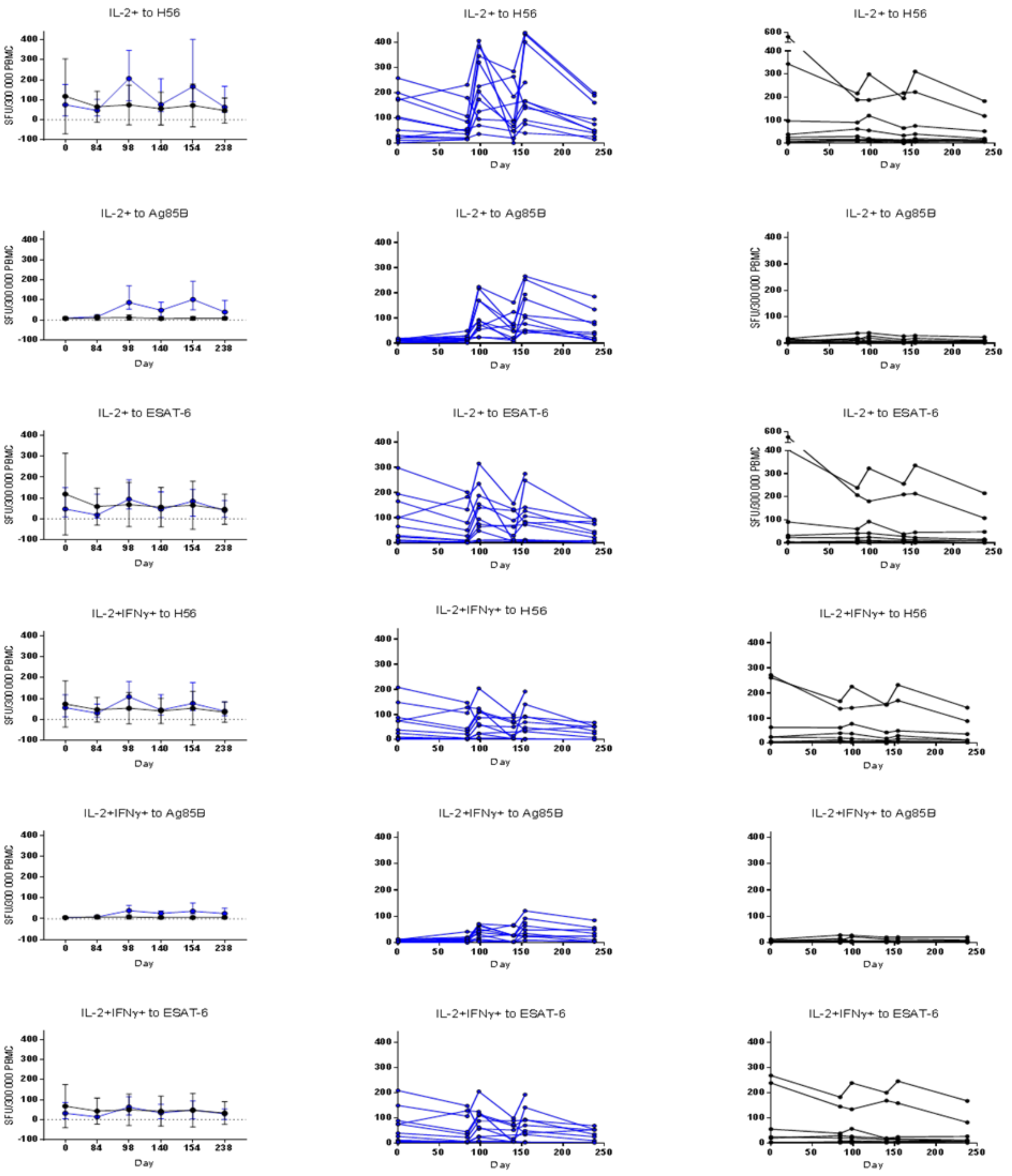

Supplementary Figure 5. Immunogenicity of the H56:IC31 vaccine. Effect of the H56:IC31-vaccine administered at two doses (day 84 and day 140) on vaccine-induced immunity in the H56:IC31 group (blue, n=12) and controls (grey, n=10) (Hypothesis 2, table 3) at day 84 and day 154. Cytokine responses (IFNγ and IL-2) to H56 fusion protein and the individual peptides (ESAT-6 and Ag85B) are measured by Fluorospot assay. **a** IFNγ T-cell responses to the H56 fusion protein in the H56:IC31 group and controls at day 84 (n: 12/10) and day 154 (n: 11/10). Boxplots are shown with median, interquartile ranges (IQR) and minimum/maxium values. Median regression with treatment as only independent explanatory factor was applied for immunogenicity read-outs pre-assigned a hierarchical order to compensate for lack of multiple testing adjustments. Effect estimates of primary priority and 95% confidence intervals based on 1000 bootstrap replications and corresponding two-sided p-values depicted in Table 3 (main paper) and denoted in **a.** **b** Descriptive line plots with median and IQR, and individual trajectories at different time points during follow-up for both H56:IC31 and control group.

Supplementary Figure 6

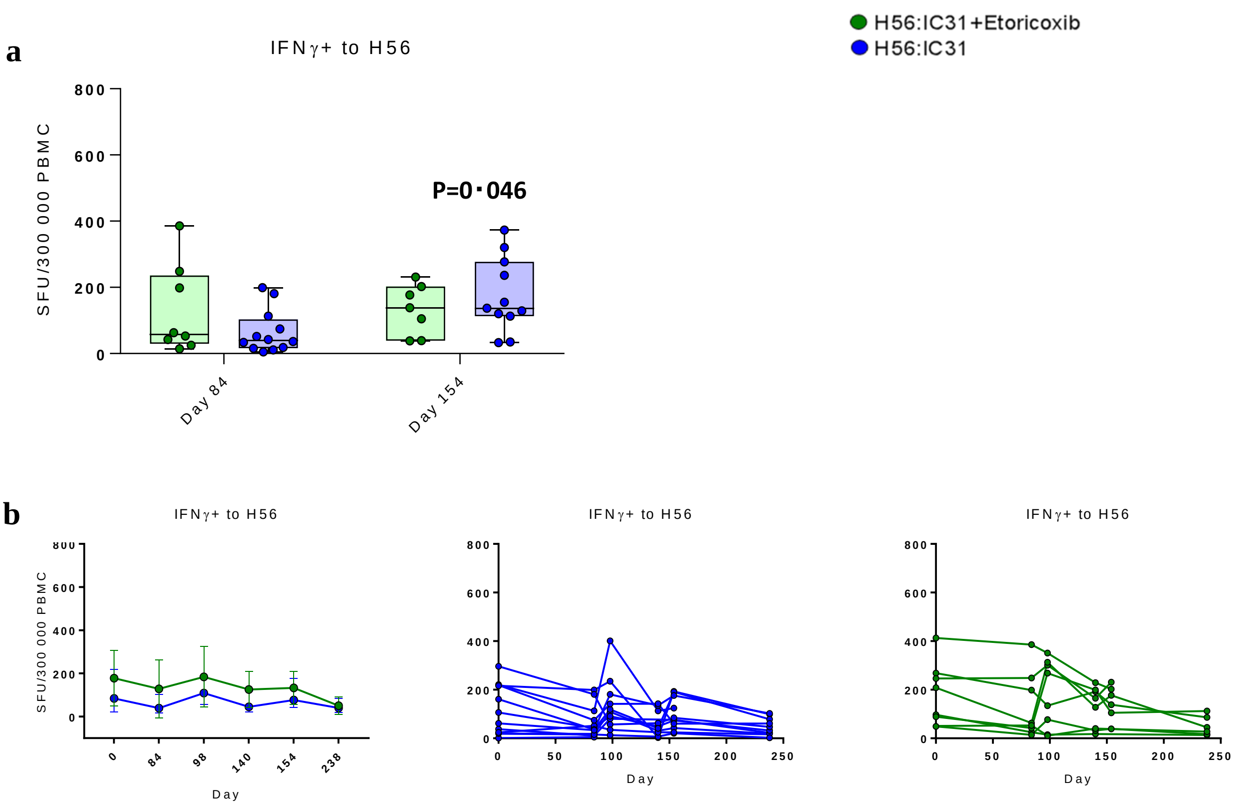

Supplementary Figure 6. Adjunctive effects of etoricoxib on H56:IC31 immunogenicity. H56:IC31-vaccine administered at two doses (day 84 and day 140) in the etoricoxib+H56:IC31-group (green, n=8) and the H56:IC31 group (blue, n=12) and (Hypothesis 3, table 3) at day 84 and day 154. IFNγ T-cell responses to H56 fusion protein are measured by Fluorospot assay. **a** IFNγ T-cell responses to the H56 fusion protein in the etoricoxib+H56:IC31-group and the H56:IC31 group at day 84 (n: 8/12) and day 154 (n: 7/11). Boxplots are shown with median, interquartile ranges (IQR) and minimum/maxium values. Median regression with treatment as only independent explanatory factor was applied for immunogenicity read-outs pre-assigned a hierarchical order to compensate for lack of multiple testing adjustments. Effect estimates of primary priority and 95% confidence intervals based on 1000 bootstrap replications and corresponding two-sided p-values depicted in Table 3 (main paper) and denoted in **a. b** Descriptive line plots with median and IQR, and individual trajectories at different time points during follow-up for both H56:IC31 and control group.

Supplementary Figure 7

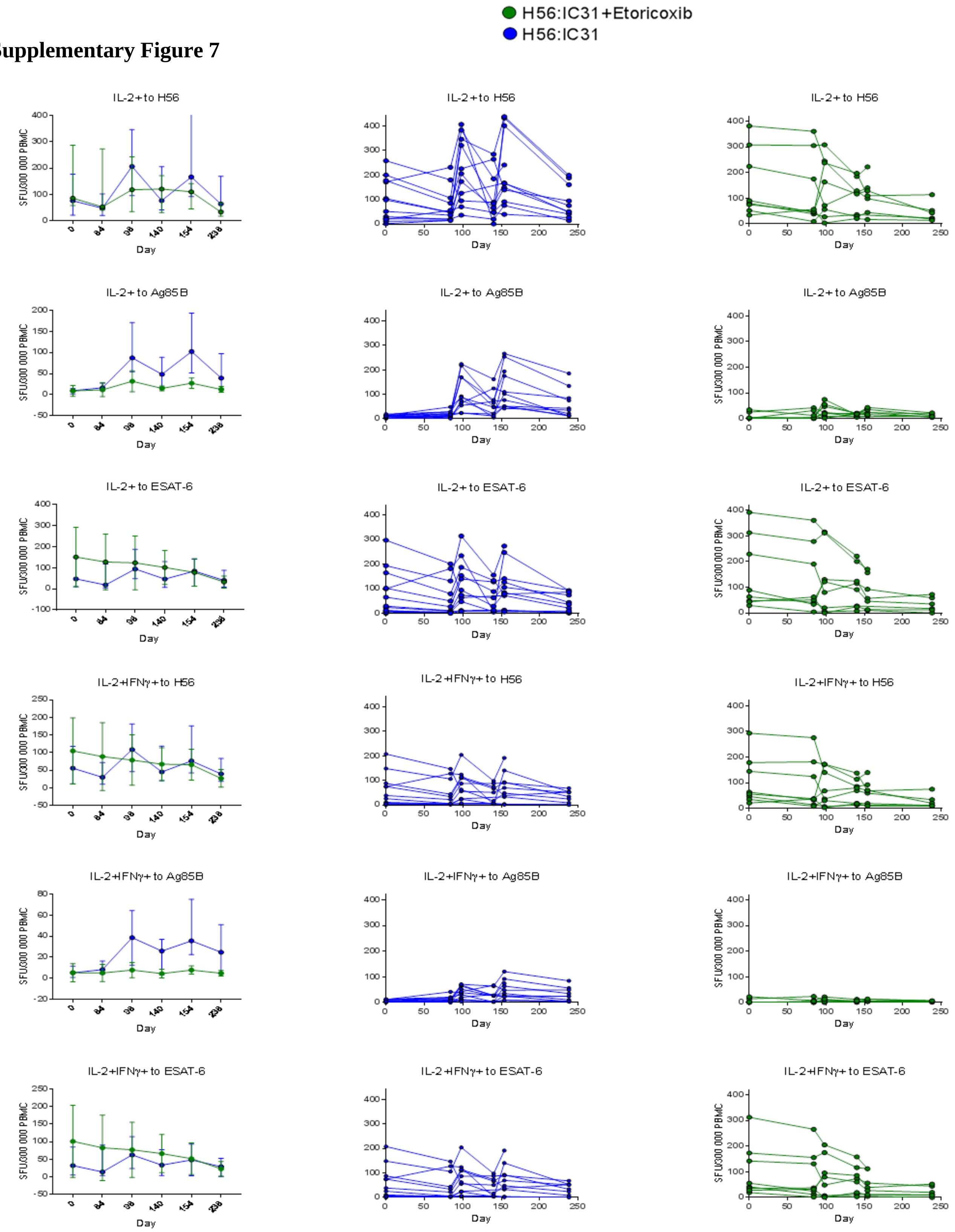

Supplementary Figure 7. Adjunctive effects of etoricoxib on H56:IC31 immunogenicity. H56:IC31-vaccine administered at two doses (day 84 and day 140) in the etoricoxib+H56:IC31-group (green, n=8) and the H56:IC31 group (blue, n=12) and (Hypothesis 3, table 3) at day 84 (n: 8/12) and day 154 (n: 7/11). Cytokine responses (IL-2 and IL-2+ IFNγ) to H56 fusion protein and the individual peptides (ESAT-6 and Ag85B) are measured by Fluorospot assay. Descriptive line plots with median and IQR, and individual trajectories at different time points during follow-up for the etoricoxib+H56:IC31-group and the H56:IC31 group.

Supplementary Figure 8

Whole blood intracellular cytokine staining (WB-ICS) assay

red=Etoricoxib, blue=H56:IC31, black=Controls, green=Etoricoxib+H56:IC31

Cytokine+ CD4 T cell responses to PPD

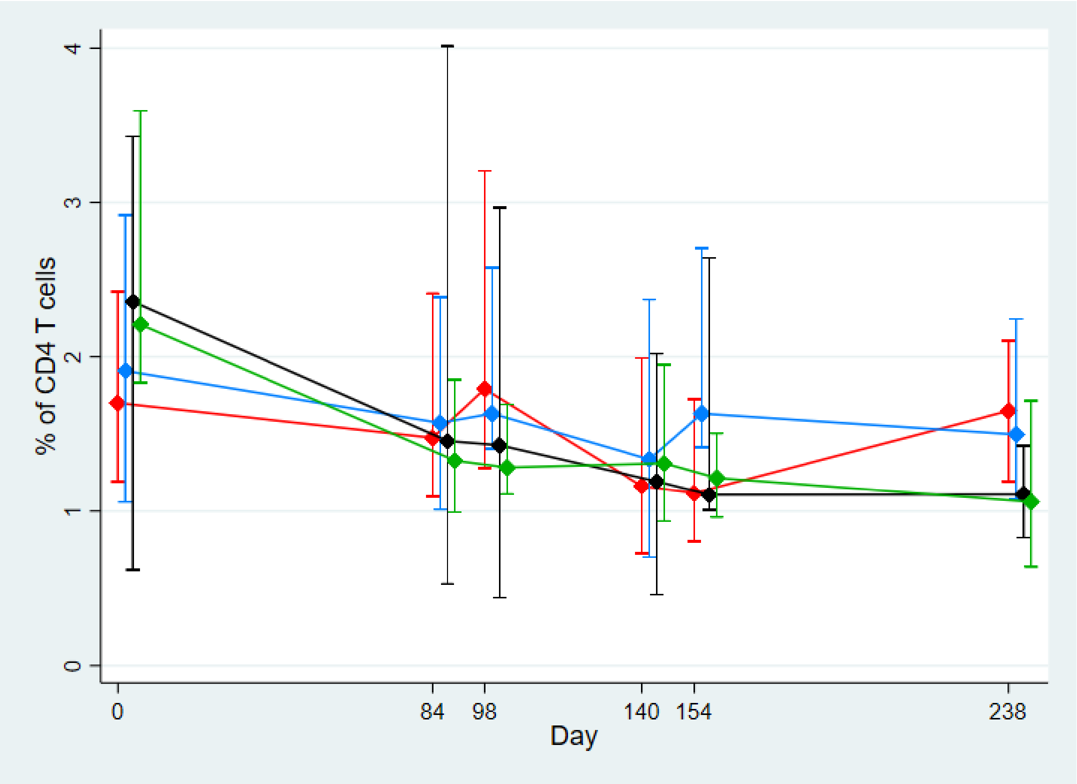

Cytokine+ CD4 T cell responses to Ag85B, ESAT-6 and Rv2660c

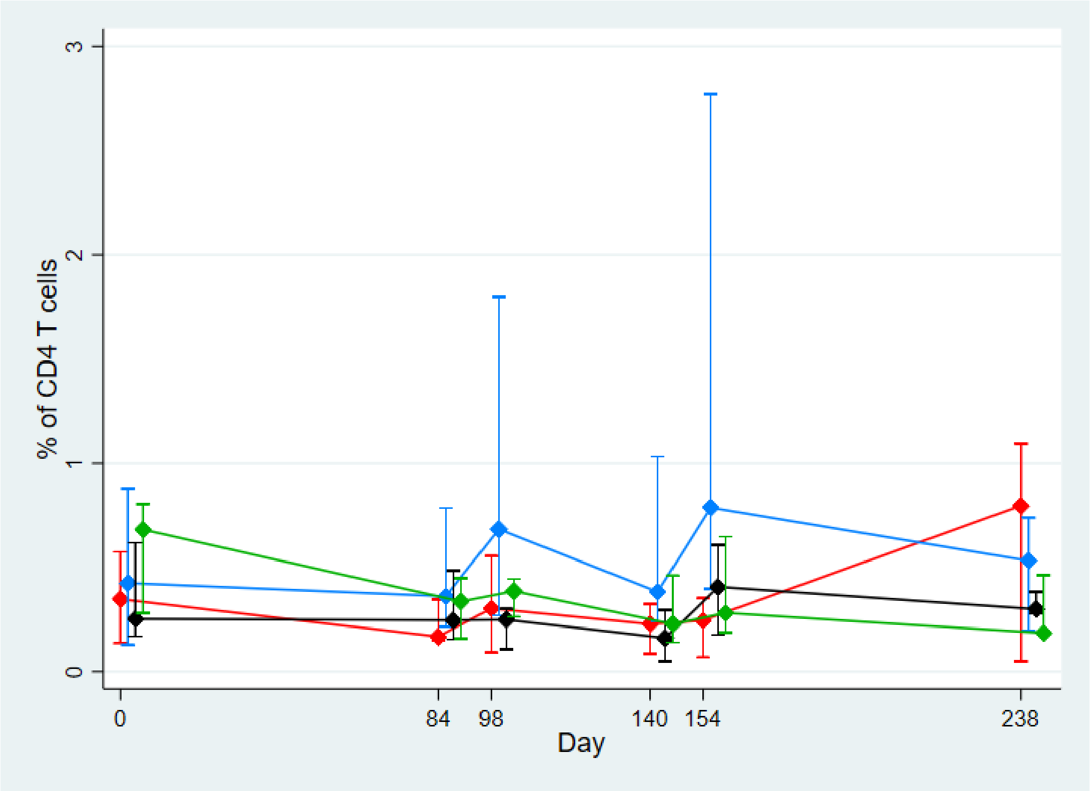

Cytokine+ CD4 T cell responses to ESAT-6

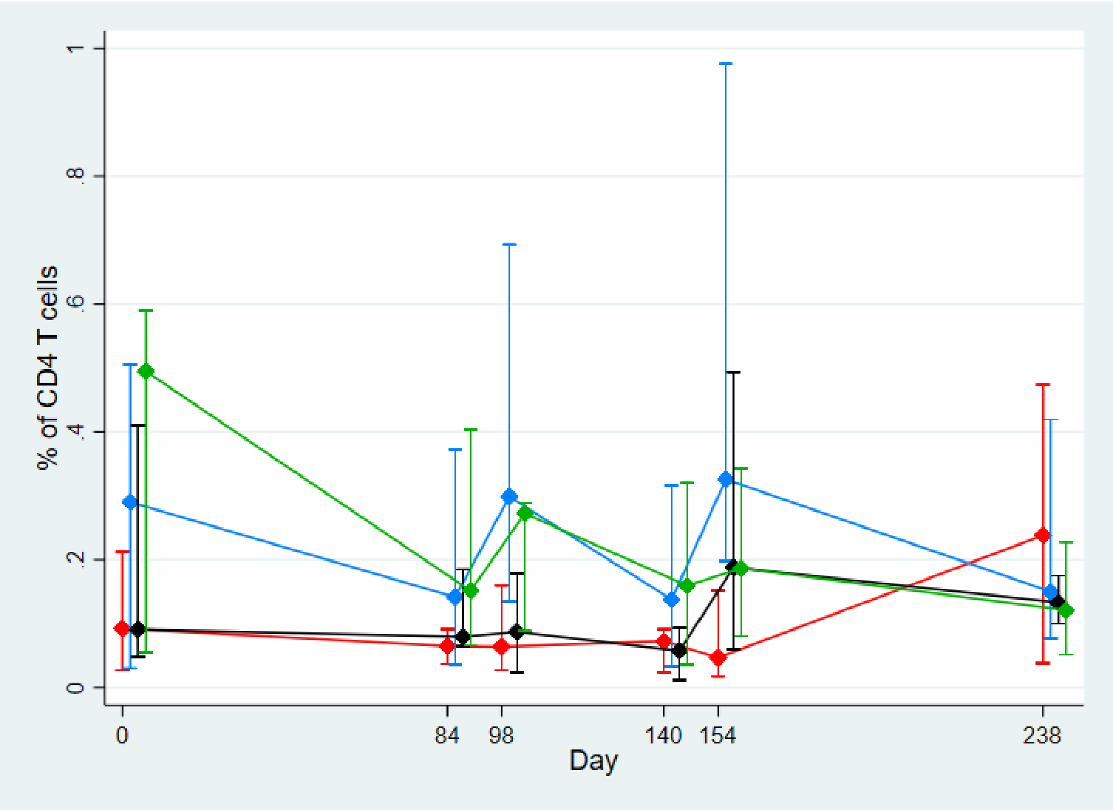

Cytokine+ CD4 T cell responses to Ag85B

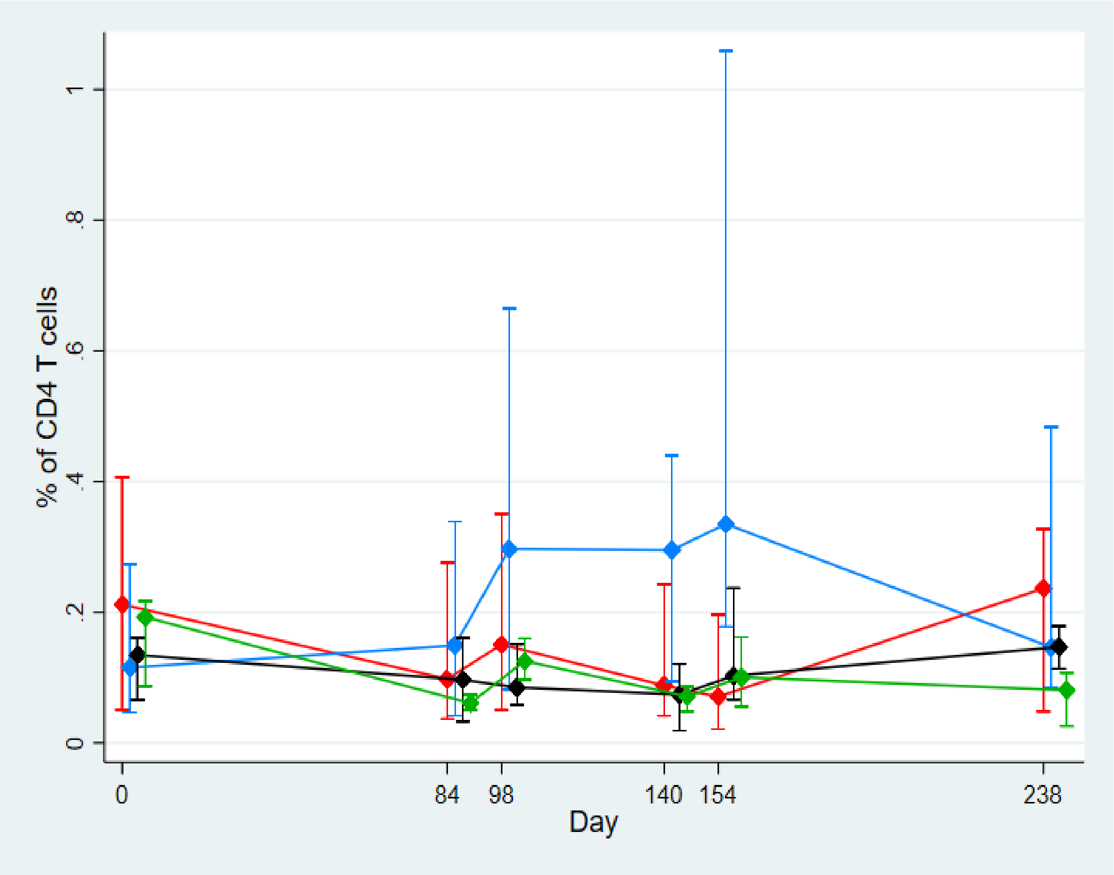

Cytokine+ CD4 T cell responses to Rv2660c

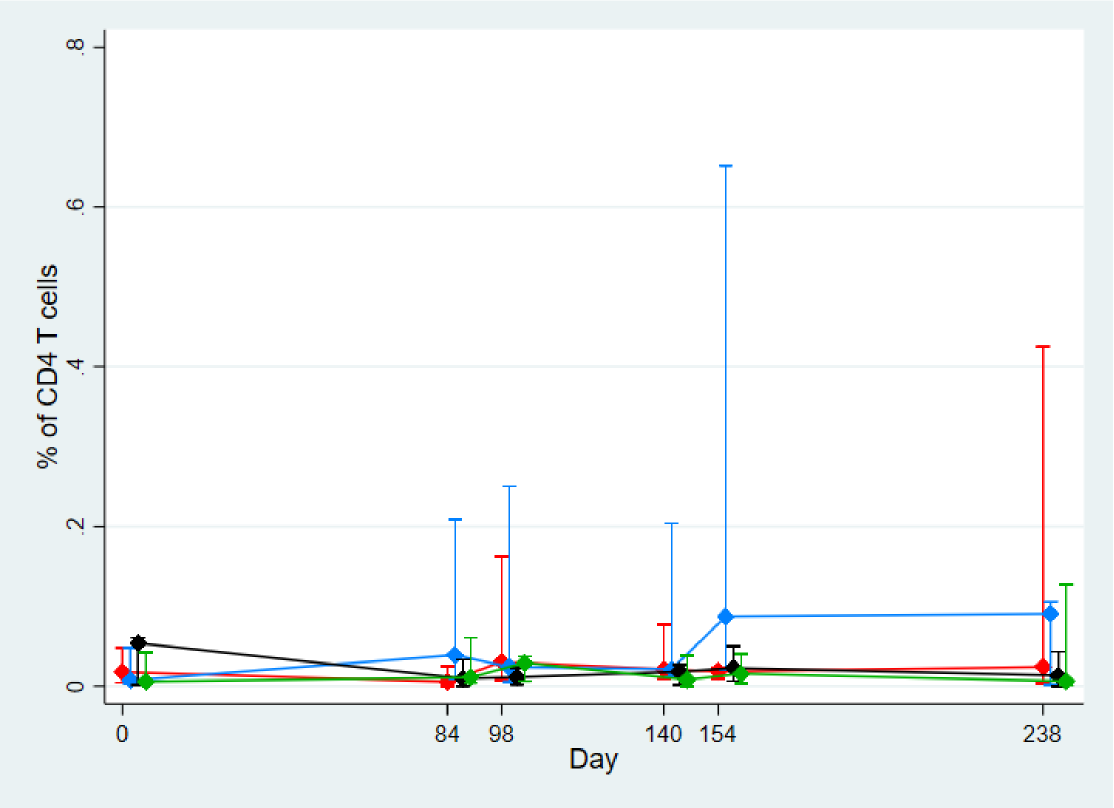

IFNγ+IL2+TNFα+ CD4 T cell responses to Ag85B, ESAT-6 and Rv2660c

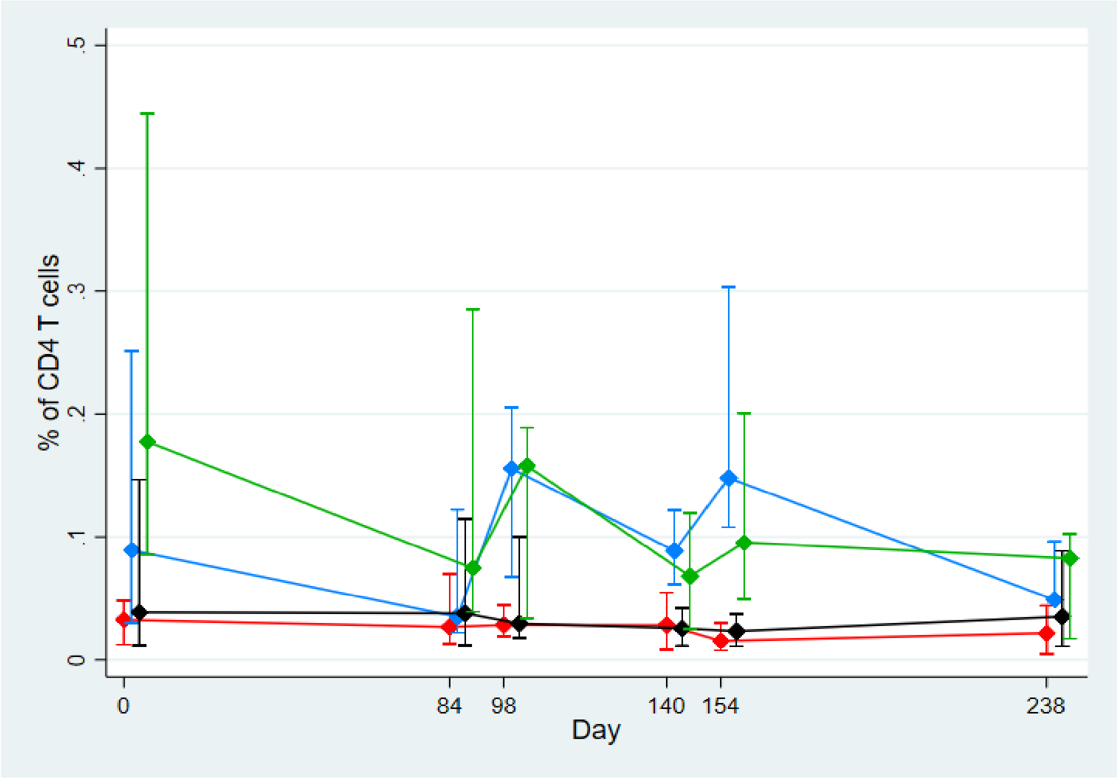

IFNγ+IL2+TNFα+ CD4 T cell responses to ESAT-6

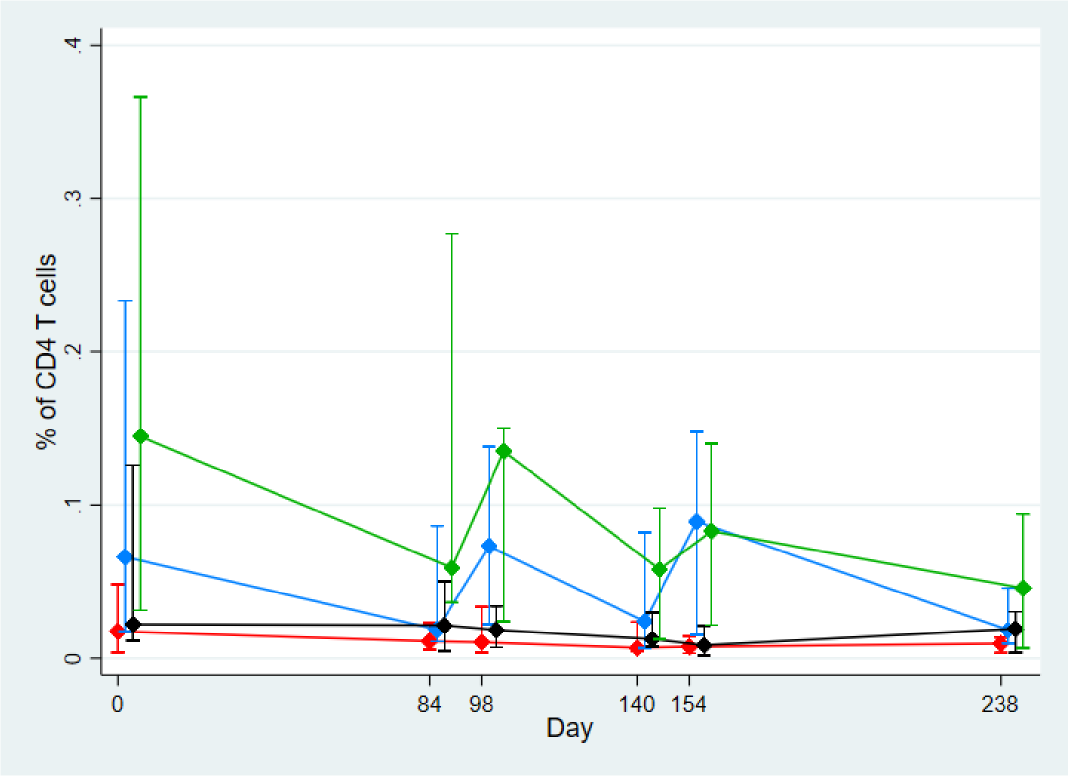

IFNγ+IL2+TNFα+ CD4 T cell responses to Rv2660c

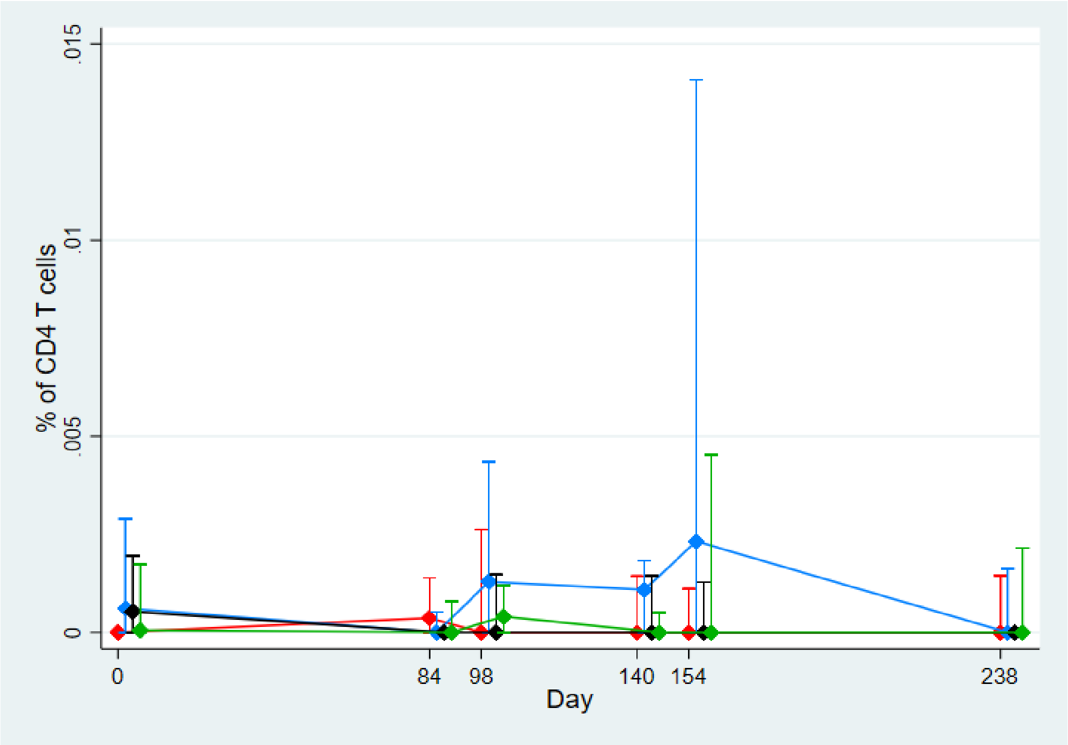

IFNγ+IL2+TNFα+ CD4 T cell responses to Ag85B

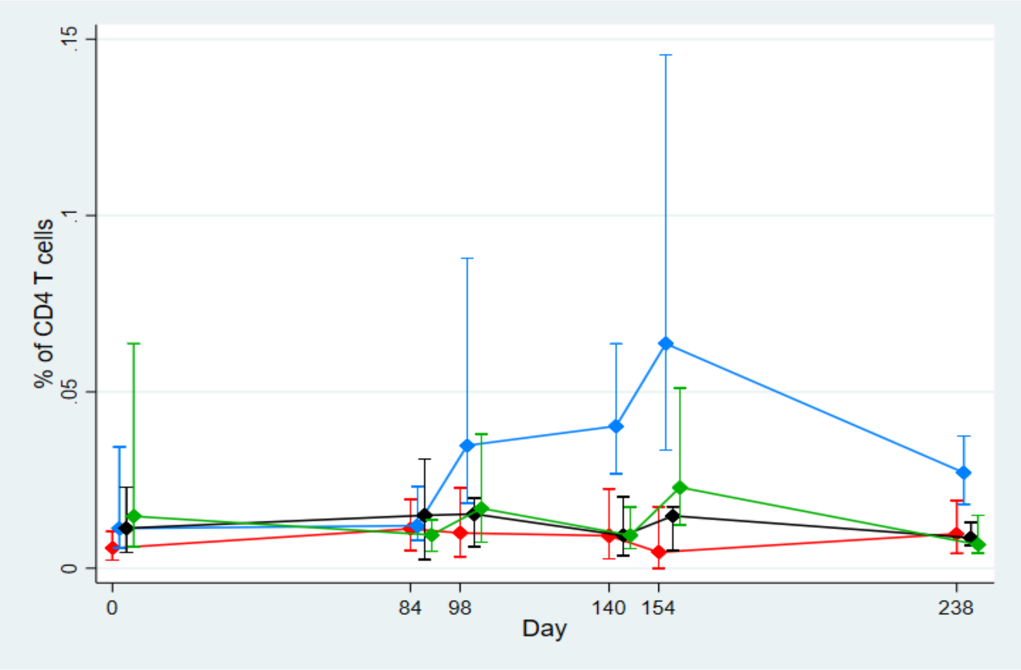

IFNγ+IL2+TNFα- CD4 T cell responses to Ag85B, ESAT-6 and Rv2660c

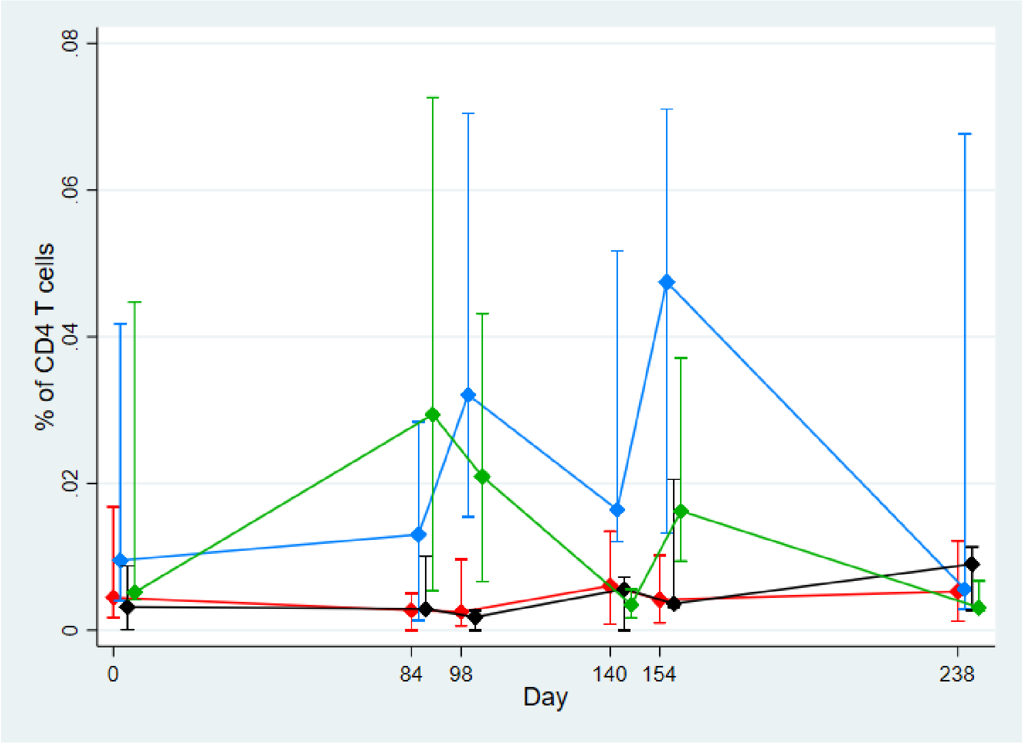

IFNγ+IL2+TNFα- CD4 T cell responses to ESAT-6

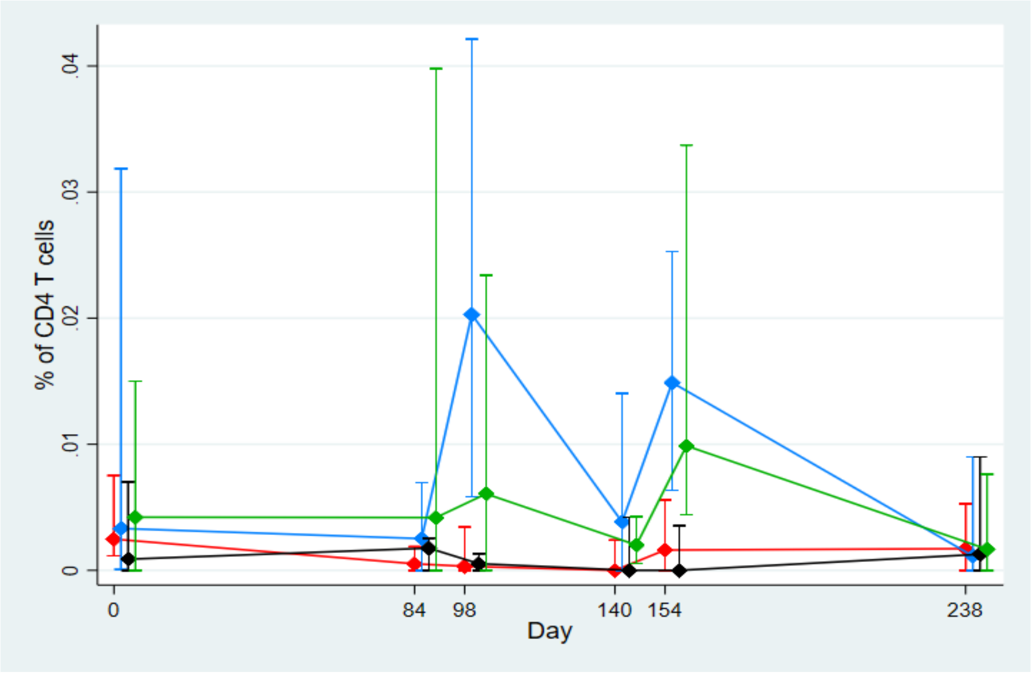

IFNγ+IL2+TNFα- CD4 T cell responses to Ag85B

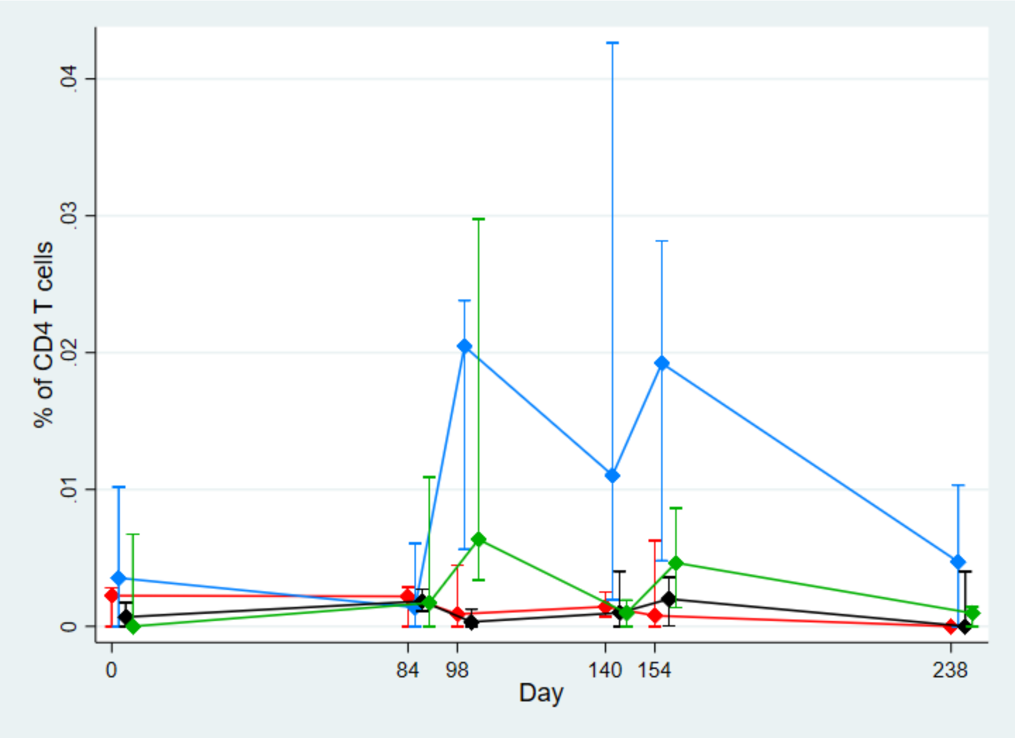

IFNγ+IL2+TNFα- CD4 T cell responses to Rv2660c

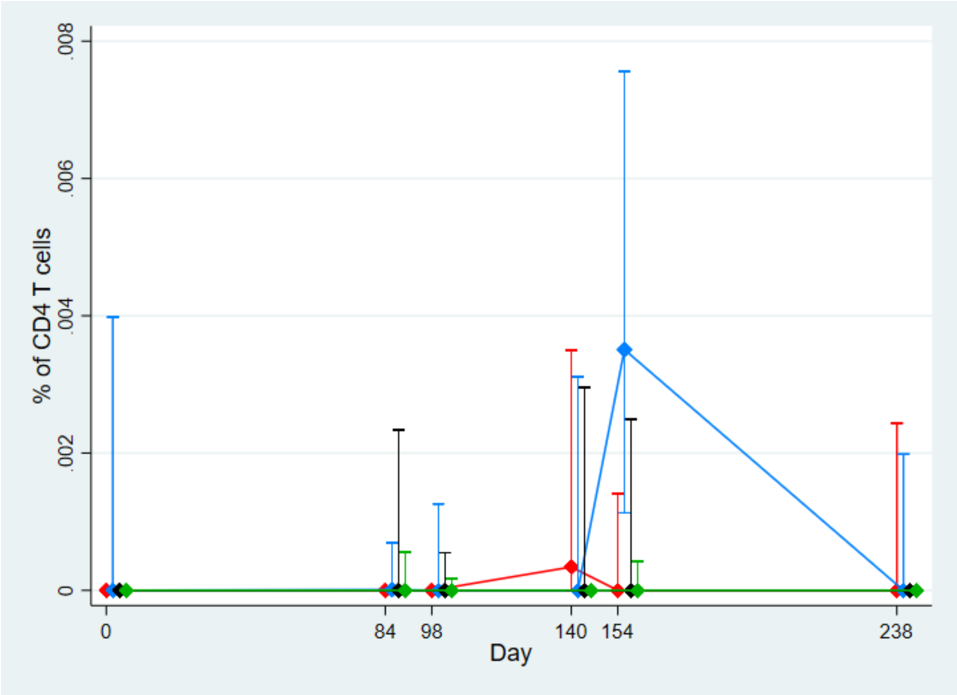

IFNγ-IL2+TNFα+ CD4 T cell responses to ESAT-6

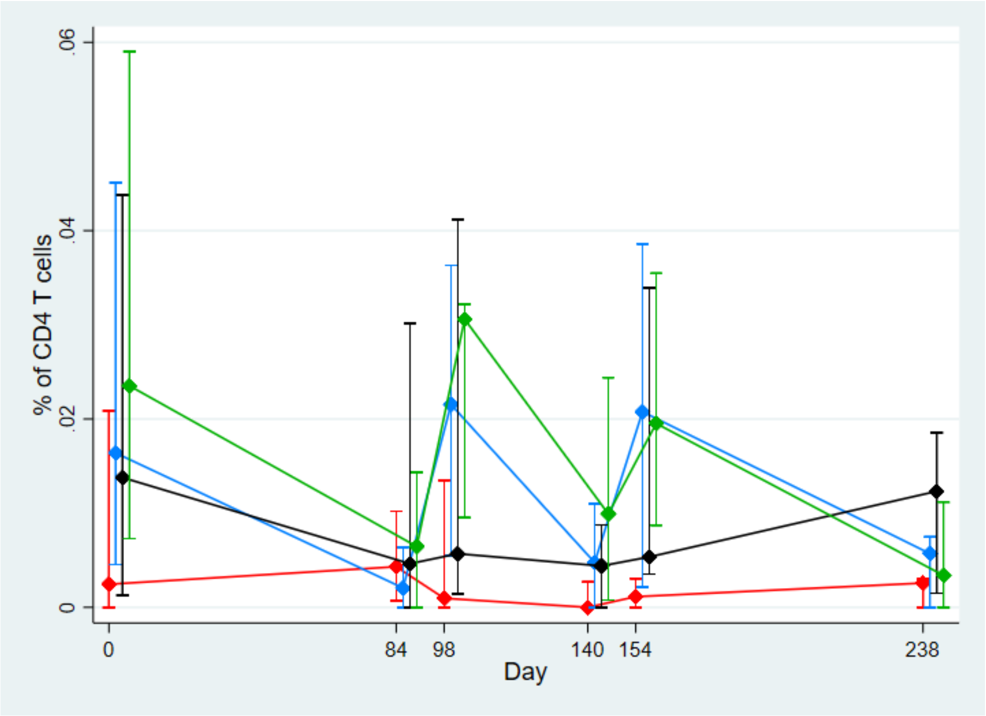

IFNγ-IL2+TNFα+ CD4 T cell responses to Ag85B

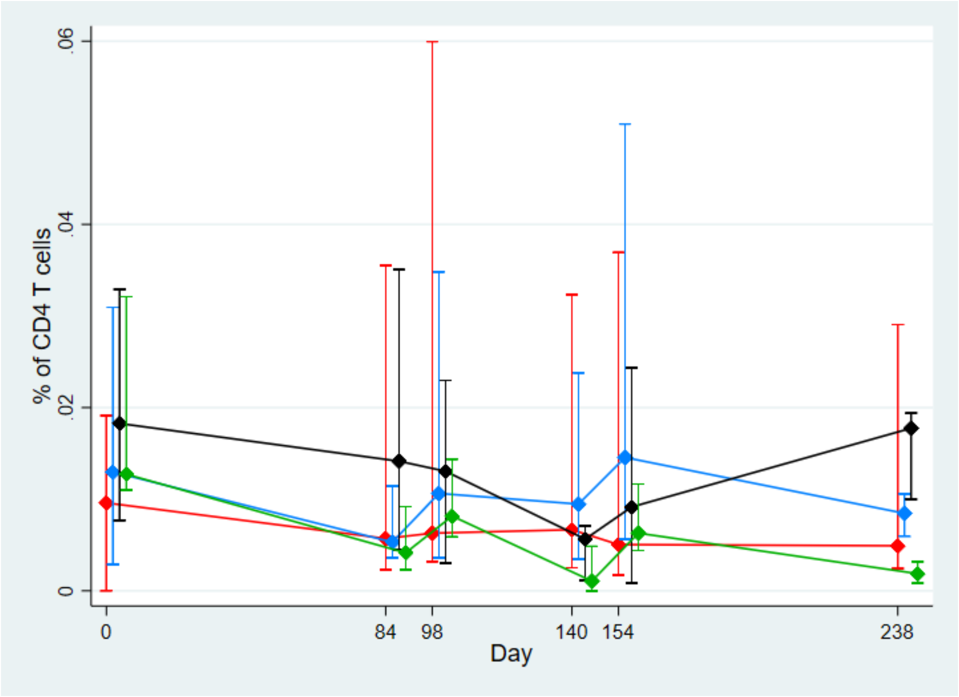

IFNγ-IL2+TNFα+ CD4 T cell responses to Rv2660c

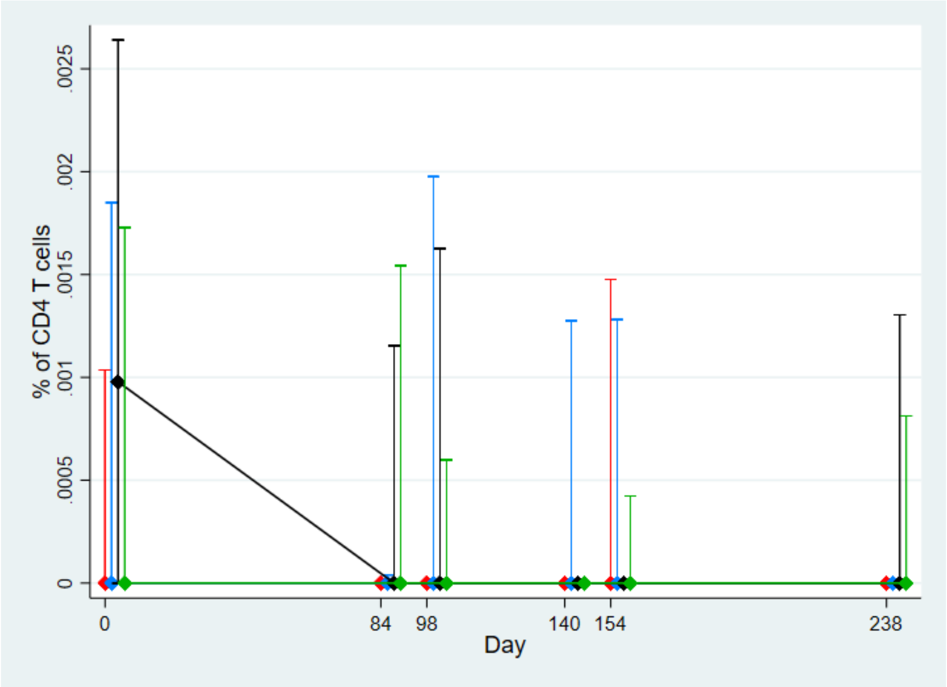

IFNγ-IL2+TNFα+ CD4 T cell responses to Ag85B, ESAT-6 and Rv2660c

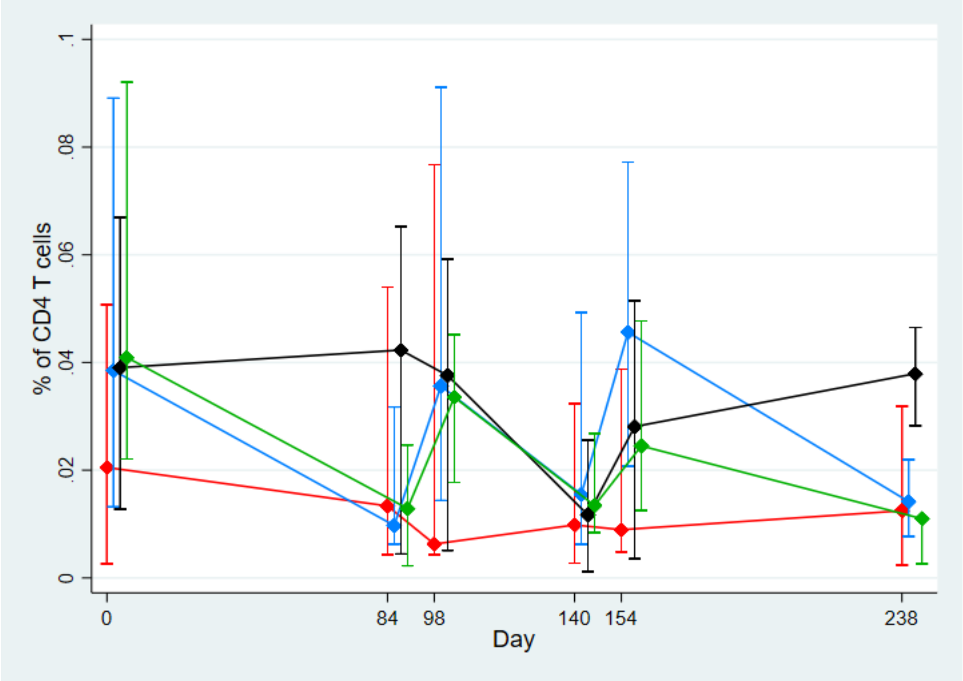

IFNγ+IL2-TNFα+ CD4 T cell responses to Ag85B, ESAT-6 and Rv2660c

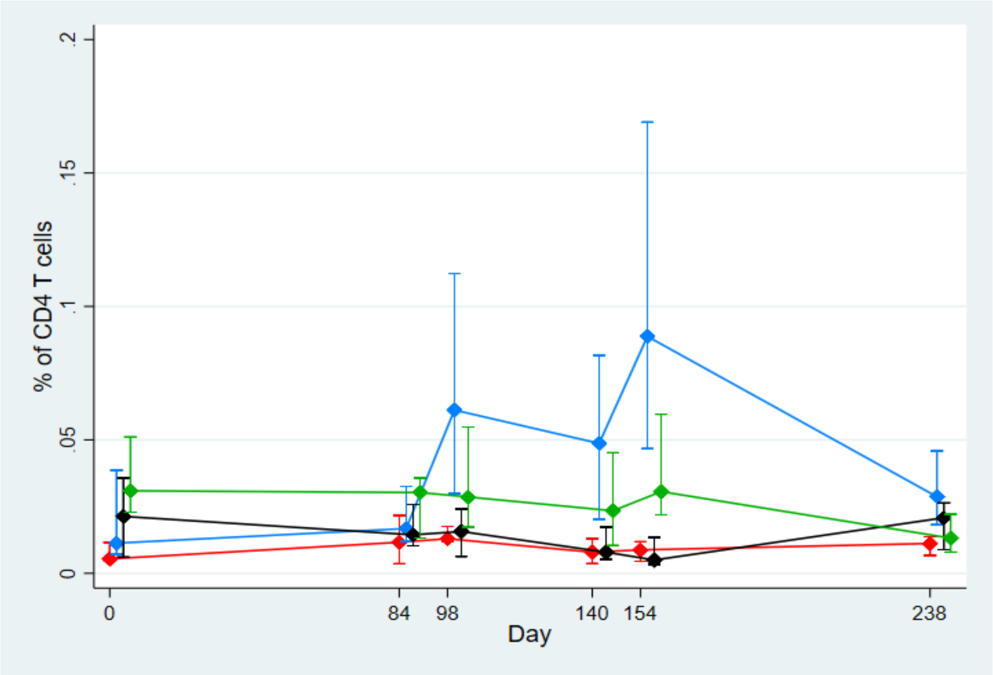

IFNγ+IL2-TNFα+ CD4 T cell responses to Rv2660c

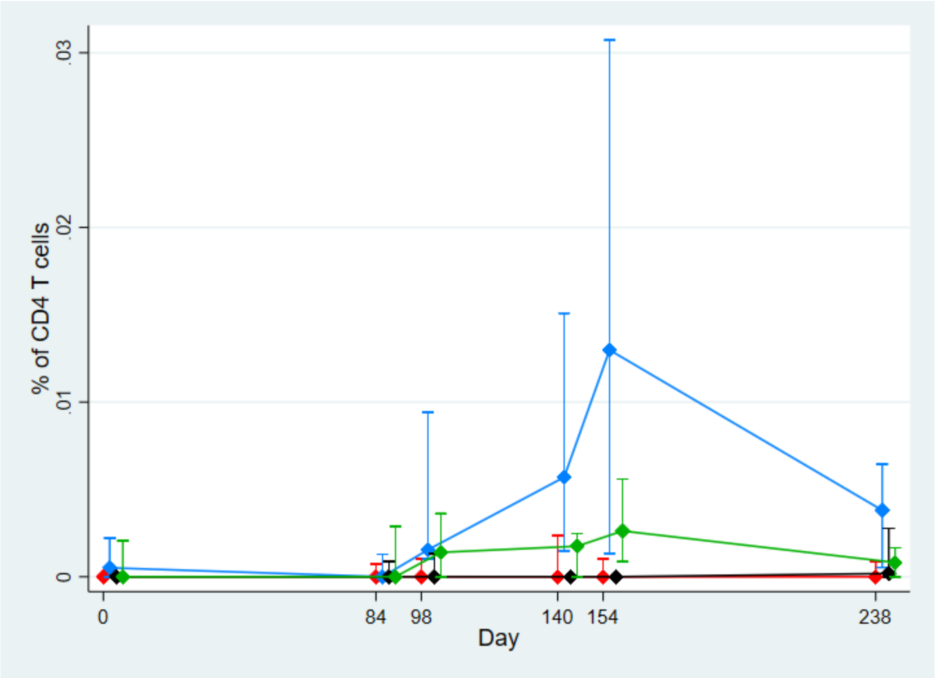

IFNγ+IL2-TNFα+ CD4 T cell responses to Ag85B

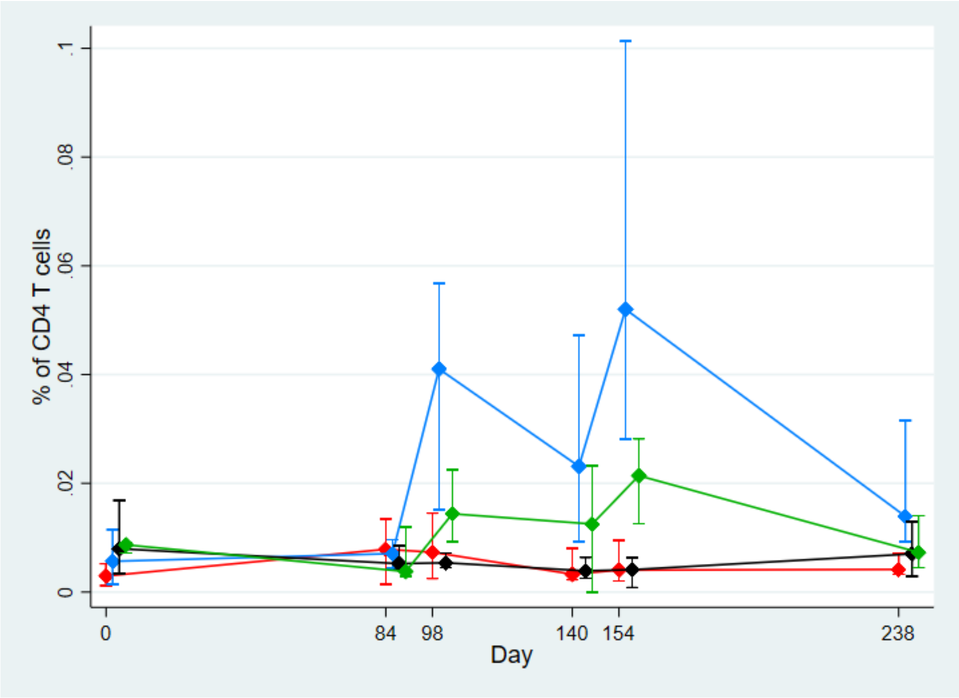

IFNγ+IL2-TNFα+ CD4 T cell responses to ESAT-6

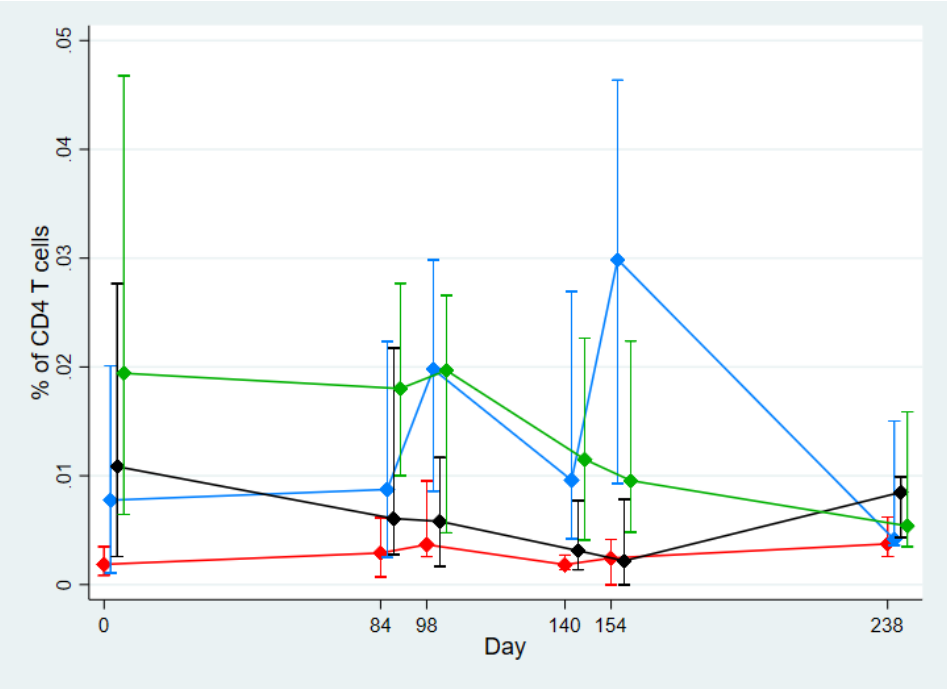

Supplementary Figure 8. Cytokine responses for all study groups at all time points. Descriptive line plots of cytokine responses measured by flow cytometry (WB-ICS assay) from day 0 to day 238 with median and IQR for all study groups etoricoxib-group (red, n=10), H56:IC31-group (blue, n=12), controls (black, n=10) and etoricoxib+H56:IC31 group (green, n=8) are depicted for all possible combinations of Cytokine+ T-cells to the individual *Mtb* peptides ESAT-6, Ag85B and Rv2660c and all peptides combined.

Supplementary Methods

Clinical Study Protocol

Therapeutic vaccination and immune modulation -

new treatment strategies for the multidrug-resistant tuberculosis pandemic

An open label phase I clinical trial of the therapeutic TB H56:IC31 vaccine and cyclooxygenase-inhibitors

Protocol Identification Number: TBCOX2

EudraCT Number: 2014-004986-26

ClinicalTrials.No: NCT02503839

**SPONSOR:**	**Oslo University Hospital**
**PRINCIPAL INVESTIGATOR (PI):**	Professor Anne Ma Dyrhol-Riise<br>Oslo University Hospital<br>Department of Infectious Diseases, Oslo, Norway<br>Tel: +47 92857261<br>E-mail: a.m.d.riise@medisin.uio.no

PROTOCOL VERSION 6.0 - 12-10-2016

Confidentiality Statement
The information contained in this document is confidential and cannot be disclosed unless required by governmental regulation. Persons to whom any portion of the contents of this document is disclosed must be informed that the information is confidential and may not be further disclosed by them.

CONTACT DETAILS

Sponsor

Professor Dag Kvale, MD, PhD

Oslo University Hospital

Department of Infectious Diseases, Oslo, Norway

Tel: +47 95200709

E-mail: dag.kvale@medisin.uio.no

Principal investigator:

Professor Anne Ma Dyrhol-Riise, MD, PhD

Oslo University Hospital

Department of Infectious Diseases, Oslo, Norway

Tel: +47 92857261

E-mail: a.m.d.riise@medisin.uio.no

Co-investigator:

Professor Kjetil Taskén, MD, PhD

The Biotechnology Centre

University of Oslo, Oslo, Norway

Tel: +47 90860759

E-mail: kjetil.tasken@biotek.uio.no

Co-investigator:

Morten Ruhwald, MD, PhD

Head of section, Human Immunology

Infectious Disease Immunology

Division of vaccine, Statens Serum Institut

Tel: +45 3268 3940

E-mail: moru@ssi.dk

Co-investigator:

Professor Tehmina Mustafa, MD, PhD

Dep. of Thoracic Medicine

Haukeland University Hospital, Bergen, Norway

Tel: +47 97633677

E-mail: tehmina.mustafa@uib.no

APPROVAL PAGE

I hereby declare that I will conduct the study in compliance with the Protocol, ICH GCP and the applicable regulatory requirements:

Principal Investigator:

____

Prof. Anne Ma Dyrhol-Riise, MD, PhD Date

Sponsor:

____

Prof. Dag Kvale, MD, PhD Date

Co-investigator:

____

Prof. Kjetil Taskén, MD, PhD Date

Co-investigator:

____

Morten Ruhwald, MD, PhD Date

Co-investigator:

____

Tehmina Mustafa, MD, PhD Date

PROTOCOL SYNOPSIS

Name of Sponsor/Company: Oslo University Hospital

Name of Products: Arcoxia (MSD). Tuberculosis vaccine: H56:IC31

Name of Active Ingredient: etoricoxib. H56 antigen and IC31 adjuvant.

Title of Study: *Therapeutic vaccination and immune modulation - new treatment strategies for the multidrug-resistant tuberculosis pandemic. A open label phase I clinical trial of the therapeutic TB H56:IC31 vaccine and cyclooxygenase-inhibitors (COX-2i)*

EudraCT nr: 2014-004986-26**.**

Protocol Identification Number: TBCOX2

Investigators and Study Centre(s):

Professor Anne Ma Dyrhol-Riise, MD, PhD, Oslo University Hospital, Oslo, Norway (PI)

Professor Dag Kvale, MD, PhD, Oslo University Hospital, Oslo, Norway.

Professor Kjetil Taskén, MD, PhD, University of Oslo, Oslo, Norway.

Morten Ruhwald, MD, PhD, Statens Serum Institut, København, Denmark.

Professor Tehmina Mustafa, MD, PhD, Haukeland University Hospital, Bergen, Norway.

Study centre: Oslo University Hospital. Haukeland University Hospital.

Phase of Development: Phase I

Objectives:

1. Study the **safety** and **tolerability** of etoricoxib given for 20 weeks (day 0-140) alone and in combination with the therapeutic TB vaccine H56:IC31 given as two immunizations at day 84 and day 140 in patients with active TB disease treated with conventional 26-week anti-TB chemotherapy.
2. Study the **immune effects** of etoricoxib given for 20 weeks (day 0-140) on immune regulation and TB vaccine (H56:IC31) **immunogenicity** in patients with active TB disease treated with conventional 26-week anti-TB chemotherapy.

Methodology: Open-label controlled, randomized study

Number of Subjects Planned: A total of 40 patients with active pulmonary and/or extra-pulmonary TB disease infected with drug-sensitive *Mtb* strains will be included sequentially in two groups A and B. Group A comprises the following study arms: Arm #1, etoricoxib (n=10), Arm #2 H56:IC31 TB vaccine (n=10) and Arm #3 as controls (n=5. Following safety review at day 98 we will include for part B comprising Arm #4 H56:IC31 TB vaccine and etoricoxib (n=10) and the remainder of controls for Arm #3 (n=5).

Diagnosis and Main Criteria for Inclusion:

1. Age between 18 and 70 years at the time of randomization
2. Microbiologically confirmed pulmonary TB (culture and/or PCR + susceptibility testing) and/or microbiologically confirmed extra-pulmonary TB (culture and/or PCR + susceptibility testing)
3. Drug sensitive *Mtb* strains (except single resistance where fully adequate anti-TB chemotherapy regimen could be provided).
4. Is willing and likely to comply with the trial procedures and is prepared to grant authorized persons access to their medical record.
5. Has completed the written informed consent process prior to the start of screening evaluations.
6. Females: Ability to avoid pregnancy during the trial.

<u>Subjects may receive H56:IC31 vaccination (study arm 2 and 4) if they meet the following criteria:</u>

1. Sputum obtained prior to 1th immunization (day 84) must be *Mtb* negative evaluated by at least two consecutive AFS or PCR/GeneXpert at least 7 days apart.

2. Documented reduction in the extent of TB disease at the infectious site(s) within day 84 evaluated by physical and/or radiological examination (table A2).
3. Clinical improvement with normal vital signs (blood pressure, temperature and pulse), improvement of any TB related symptoms to Grade 1-3 (table A2), stable or increased body-weight and reduced inflammatory blood parameters (CRP, ESR and WBC counts) compared to baseline.

Main exclusion criteria:

(i) Study-specific: Disseminated TB. Evidence of a new acute illness that may compromise the safety of the subject in the trial on study day 0. History of autoimmune disease or immunosuppression. History or laboratory evidence of any possible immunodeficiency state. Anemia (<9 g/100 ml). HIV sero-positivity. Chronic hepatitis B (HBs antigen positive) with increased liver transaminases (ASAT, ALAT) or chronic hepatitis C (HCV RNA positive). Concomitant or sporadic use of NSAID or corticosteroids (>2 times per week). Other immune modulating therapies including DMARDS. Total cholesterol > 7 mmol/L. Hypertension >140/90 mm Hg (treated or untreated) or treated with >1 antihypertensive drug at any blood pressure. Cardiovascular events or stroke in parents, siblings or off-springs occurring < 55 years of age. Serum creatinine above reference levels (females > 90 µmol/L; males > 105 µmol/L). Known diabetes mellitus type I or diabetes mellitus type II with HbA1c >7%. Pregnancy (S-hCG >5 IU/l for females at childbearing age). Breastfeeding.

(ii) Exclusion criteria for etoricoxib according to SPC: Known hypersensitivity for etoricoxib or etoricoxib tablet substances. Known hypersensitivity for sulphonamides. Active peptic ulcer or gastrointestinal haemorrhage. History of asthma, acute rhinitis, nasal polyps, angioneurotic oedema, urticaria or other allergic reactions after taking acetyl salicylic acid or NSAID including COX-2 inhibitors. Moderate/severe deranged liver function (Child-Pugh >7). Serum-creatinine clearance <30 ml/min. Inflammatory bowel disease. Heart failure (NYHA II-IV). Established ischaemic heart disease, peripheral arteriosclerosis and/or cerebrovascular disease, including previous myocardial infarction, angina pectoris, unstable angina, PCI or coronary bypass, previous transitory ischemic attack or apoplexia/stroke.

iii) **Exclusion criteria for H56:IC31:** Known hypersensitivity for vaccines or vaccine adjuvants.

Investigational Product, Dose and Mode of Administration:

Arcoxia (etoricoxib**)** tablets 120 mg, administered p.o. at 120 mg x 1 starting at the same time as initiation of anti-TB chemotherapy (study day 0) and for 20 weeks (day 0-140). If intolerable adverse reactions or clinical laboratory evaluation category 2 occur, dose will be reduced to 90 mg x1 or drug will be stopped.

H56:IC31 vaccine; administrated i.m at 5 µg 1st dose at study day 84 and 2nd dose at study day 140.

A phase I trial (C-032-456; open-label, dose-escalation of latent TB) and a phase I/IIa (C-035-456; randomized, placebo-controlled) with H56:IC31 show that the vaccine is well tolerated and no safety issues have been identified. A phase I/II clinical study of H56:IC31 (C-037-456) in patients treated for active pulmonary TB is ongoing in South-Africa. The first participant was dosed in January 2015 and after 12 first doses administered (19th February 2015) no safety concerns have been reported. Inclusion in study arm 3 and 4 will not start before all preliminary safety data from this study is available and the vaccine is proven safe also in this population.

Study Duration*:* Informed consent, inclusion, baseline assessments and start of standard anti-TB chemotherapy at day 0 followed by 20 weeks of study drug administration (day 0-140), 6 weeks (or according to clinical evaluation) of standard anti-TB chemotherapy alone (day 0-182) and follow-up control at day 210 and end of study visit day 238.

Criteria for Evaluation:

Safety: Tolerability and safety of etoricoxib and the therapeutic TB vaccine H56:IC31. Reductions of etoricoxib dose or stop of drug, AE/SAE/SUSAR including cardiovascular events, blood pressure, clinical chemistry and vaccine AE/SAE/SUSAR.

Pharmacokinetics: AUC, C_{max} of etoricoxib at steady state after 7 days (Arm #1).

Primary Immunogenicity readouts:

1. Etoricoxib effect of TB specific immune responses: T cell proliferation and cytokine responses (change between day 0 and 140).
2. H56:IC31 vaccine immunogenicity: T cell proliferation and cellular cytokine responses 14 days post-immunization (day 98 and day 154).

3. Etoricoxib effect of H56:IC31 vaccine immunogenicity: T cell proliferation and cellular cytokine responses 14 days post-immunization (day 98 and day 154).<br>**Readouts:** 1. WB/PBMC ICS (cytokines responses). 2. T cell proliferation (cfse), 3. Tregs/maturity (CD25, FoxP3, COX-2). 4. Soluble biomarkers. 5. Monocyte responses (COX-2). 6. RNA analysis.
**Statistical Methods:** Non-parametrical methods to describe patient groups including matched pair statistics to determine primary endpoints within the four study arms.

TABLE OF CONTENTS

Page No.

CONTACT DETAILS **2**
APPROVAL PAGE **3**
PROTOCOL SYNOPSIS **4**
TABLE OF CONTENTS **7**
LIST OF ABBREVIATIONS **10**
1 INTRODUCTION **11**
1.1 Background 11
1.1.1 The TB epidemic 11
1.1.2 Immune pathogenesis and regulation in TB 11
1.1.3 The cyclooxygenase-prostaglandin E2 pathway in TB 11
1.2 Pre-Clinical & Clinical Experience with COX-2i and H56:IC31 12
1.2.1 Pre-clinical studies of COX-2i in experimental animal TB models 12
1.2.2 Clinical studies of COX-2i 12
1.2.3 Pre-clinical studies of the H56:IC31 TB vaccine in animal TB models 12
1.2.4 Clinical studies of the H56:IC31 TB vaccine 13
1.3 Rationale for the Study and Purpose 13
1.3.1 TB therapeutic vaccines as rescue therapy in MDR-TB 13
1.3.2 COX-2i improves cellular vaccine responses *in vivo* 14
1.3.3 COX-2i improves TB specific immune responses and regulation *in vitro* 14
1.3.4 Rationale for COX-2i regimen and dose selected 14
2 STUDY OBJECTIVES AND RELATED ENDPOINTS **15**
3 OVERALL STUDY DESIGN **15**
4 STUDY POPULATION **17**
4.1 Selection of Study Population 1
4.2 Number of Patients 1
4.3 Inclusion Criteria 1
4.4 Exclusion Criteria 2
4.4.1 Study-specific exclusion criteria 2
4.4.2 Exclusion criteria according to the SPC on Arcoxia from Norwegian Medicines Agency 2
4.5 Criteria for receiving H56:IC31 vaccine 2
4.6 Withdrawal and Termination Criteria 19
5 TREATMENT **19**
5.1 Drug Identity, Supply and Storage. 20
5.1.1 Description of Arcoxia® 20
5.1.2 Description of H56:IC31 20
5.1.3 Description of placebo 20
5.2 Dosage and Drug Administration 20
5.3 Duration of Therapy 21
5.4 Clinical Monitoring 21
5.5 Drug dose Modifications 21
5.6 Concomitant Medication 21
5.7 Drug Accountability 22
5.8 Treatment Compliance 22
5.9 Drug Labeling 22
5.10 Subject Numbering and Randomization 22
6 STUDY PROCEDURES **23**
6.1 Table 2. Trial flow chart
Table A2. Evaluation of treatment response in extrapulmonal TB 24
6.2 By Visit 26
6.2.1 Before Treatment Starts 26
6.2.2 During Treatment 26
6.2.3 End of Treatment Visit 27
6.2.4 Withdrawal Visit 27
6.2.5 After End of Treatment (Follow-up) 27
6.3 Criteria for Patient Discontinuation 27
6.3.1 Rules for Discontinuing the whole Study in an Individual Subject 27

6.3.2 Specific Rules for Discontinuing etoricoxib in an Individual Subject 27
6.3.3 Rules for Discontinuing Vaccine Injections in an Individual Subject 28
6.4 Procedures for Discontinuation 28
6.4.1 Patient Discontinuation 28
6.4.2 Trial Discontinuation 28
6.5 Laboratory Tests 29
7 ASSESSMENTS....... 29
7.1 Immunogenicity and Immunology Analyses.......29
7.1.1 Immunogenicity and Immune variables 29
7.1.1.1 Flow cytometry 29
7.1.1.2 ELISPOT 29
7.1.1.3 Transcriptional (RNA) biomarker analysis.......29
7.1.1.4 Mycobacterial growth inhibition assays (MGIA): 30
7.1.1.5 IgG and humoral immunity. 30
7.1.2 Summary of Immunology Specimens and Assays (table 3) 30
7.1.3 Primary immunogenicity Endpoints 30
7.1.4 Exploratory immune studies 31
7.2 Safety and Tolerability Assessments 31
7.2.1 Medical History 31
7.2.2 Physical Examination.......31
7.2.3 Vital Signs 31
7.2.4 Clinical Laboratory Evaluation 31
7.2.5 Pharmacokinetics.......32
7.2.6 Radiology 32
7.2.7 Adverse Events (AEs).......32
8 SAFETY MONITORING AND REPORTING 32
8.1 Definitions.......32
8.1.1 Adverse Event (AE) 32
8.1.2 Serious Adverse Event (SAE).......33
8.1.3 Suspected Unexpected Serious Adverse Reaction (SUSAR) 34
8.2 Expected Adverse Events (EAE) 34
8.3 Disease Progression/Recurrence 34
8.4 Time Period for Reporting AE and SAE.......34
8.5 Recording of Adverse Events.......35
8.6 Solicited Adverse Events and Injection Site Reactions.......36
8.7 Reporting Procedure 36
8.7.1 AEs and SAEs 36
8.7.2 SUSARs.......36
8.7.3 Annual Safety Report.......37
8.7.4 Clinical Study Report 37
8.8 Procedures in Case of Emergency 37
9 DATA MANAGEMENT AND MONITORING 37
9.1 Case Report Forms (CRFs) 37
9.2 Source Data 37
9.3 Study Monitoring 38
9.4 Confidentiality and database management.......39
10 STATISTICAL METHODS AND DATA ANALYSIS....... 39
10.1 Determination of Sample Size.......39
10.2 Randomization 39
10.3 Population for Analysis.......39
10.4 Statistical Analysis.......40
10.4.1 Safety analysis.......40
10.4.2 Vital Signs 40
10.4.3 Physical Examinations.......40
10.4.4 Clinical Laboratory Measurements 40
10.4.5 Pharmacokinetics.......40
10.4.6 Immunogenicity and Other Immunology Analyses 41
10.4.6.1 Primary Immunogenicity endpoints 41
10.4.6.2 Exploratory Assays.......42
11 STUDY MANAGEMENT....... 42
11.1 Investigator Delegation Procedure.......42
11.2 Investigator’s Responsibilities 42

11.3 Protocol Adherence 43
11.4 Study Amendments 43
11.5 Audit and Inspections 43
12 ETHICAL AND REGULATORY REQUIREMENTS 43
12.1 Competent Authority and Ethics Committee Approval 44
12.2 Handling and storage of biological material 44
12.3 Informed Consent Procedure 44
12.4 Subject Identification 44
13 TRIAL SPONSORSHIP AND FINANCING 45
14 TRIAL INSURANCE 45
15 PUBLICATION POLICY 45
16 REFERENCES 46
17 APPENDIX 47
17.1 Appendix 1: Label Arcoxia 90 mg 47
17.2 Appendix 2: Label Arcoxia 120 mg 48
17.3 Appendix 3: Primary label H56:IC31 5/500 49
17.4 Appendix 4: Secondary label H56:IC31 5/500 50
17.5 Appendix 5 Toxicity Table 51
17.6 Appendix 6 DESCRIPTION OF STUDY VISITS 54

LIST OF ABBREVIATIONS

AE	-	*adverse event*
AFS	-	*acid fast staining*
ALAT	-	*alanine aminotransferase*
ASA	-	*acetyl salicylic acid*
ASAT	-	*aspartate aminotransferase*
ART	-	*antiretroviral therapy*
cAMP	-	*cyclic adenosine mono phosphate*
CFR	-	*code of federal regulations (USA)*
CHD	-	*coronary heart disease*
COX	-	*cyclooxygenase*
COX-2i	-	*cyclooxygenase-2 inhibitors*
CK	-	*creatine kinase*
CRF	-	*case record form*
CRO	-	*contract research organization*
CRP	-	*C-reactive protein*
CSA	-	*Clinical Study Agreement*
CTC	-	*Common Toxicity Criteria*
CTCAE	-	*Common Terminology Criteria for Adverse Event*
DAE	-	*Discontinuation due to Adverse Event*
DMARDS	-	*disease-modifying anti-rheumatic drugs*
DOT	-	*directly observed therapy*
EC	-	*ethics committee*
ESR	-	*Erytrocyte Sedimentation Rate*
GCP	-	*good clinical practice*
HIV	-	*human immunodeficiency virus*
hGC		*human chorionic gonadotropin i serum*
IB	-	*Investigator's Brochure*
ICF	-	*Informed Consent Form*
ICH	-	*international conference on harmonization*
ICS	-	*intracellular cytokine staining*
IFN	-	*interferon-gamma*
IMP	-	*Investigational Medicinal Product*
LTBI	-	*latent TB infection*
LDH	-	*lactate dehydrogenase*
MDR-TB	-	*multi-drug resistant TB*
Mtb	-	*mycobacterium tuberculosis*
NSAID	-	*non-steroid anti-inflammatory drug*
NYHA	-	*New York Heart Association*
PBMC	-	*peripheral blood mononuclear cells*
PCI	-	*percutan coronar intervention*
PGE	-	*prostaglandin E*
PKA	-	*protein kinase A*
SAE	-	*serious adverse event*
SOP	-	*standard operating procedure*
SPC	-	*summary of product characteristics*
SUSAR	-	*Suspected Unexpected Serious Adverse Reaction*
TNF	-	*tumor necrosis factor*
TB	-	*tuberculosis*
XDR-TB	-	*extensively drug-resistant TB*

1 INTRODUCTION

1.1 Background

1.1.1 The TB epidemic

The World Health Organization estimates that there were 9.0 million incident active tuberculosis (TB) cases and 1.5 million deaths caused by TB in 2013 (1). A fifth of previously treated TB cases with recurrent TB disease have multi-drug resistant (MDR)-TB and extensively drug-resistant TB (XDR-TB) has been reported by 84 countries. MDR-TB may take years to treat with expensive and toxic second-line drugs with uncertain efficacy. Further, the shifting of the TB epidemic towards XDR-TB with very restricted treatment options could reverse many of the major gains made in TB control and increase spread, morbidity and mortality. This calls for new treatment modalities that could be affordable and simple alternative for a substantial number of patients with MDR-TB and XDR-TB in resource-poor countries.

1.1.2 Immune pathogenesis and regulation in TB

M. tuberculosis (*Mtb*) normally enters the host through mucosal infection and targeting of the bacteria to professional phagocytic cells induces rapid innate inflammatory responses with subsequent production of cytokines and chemokines and formation of the primary granuloma surrounded by activated monocytes, macrophages and lymphocytes (2). In the majority of cases, TB infection is successfully contained, but persists as latent infection, whereas some succumb to reactivation and develop active TB, typically seen in immune suppressed patients. CD4 T cells are induced early in infection, whereas CD8 T cells, also recruited to the lung and granulomas during TB infection, expand in the late stages (3). Host control of TB is dependent on the T helper (Th) 1 cytokines interferon-gamma (IFN-γ) and tumour necrosis factor-alfa (TNF-α) as well as proper activation of macrophages, since hyper-activated macrophages are typically found in progressing TB lesions (4). The role of TNF-α is illustrated by the rapid reactivation of latent TB in humans treated with TNF-α receptor antagonists (5) and the association of TNF-α production with protection in animal models (6). Polyfunctional T cell responses (IFN-γ+IL-2+TNF-α+) have potential to serve as correlate for protective immunity since they seem to dominate in contained TB (7,8), but the T cell characteristics required for protection and optimal correlates for TB vaccine immunogenicity are yet to be established (9). T regulatory cells (Treg), a subset of CD4 T cells with immune regulatory properties that suppress Th1 cells and IFN-γ production are expanded in active TB (10,11) and may suppress proper vaccine responses. Thus, the overall contribution to TB immunity by all these immune cell subsets has implications for both the design and strategy of post exposure vaccines and proper correlates of protection and needs to be further studied.

1.1.3 The cyclooxygenase-prostaglandin E2 pathway in TB

Prostaglandin E2 (PGE2) is produced by cyclooxygenase-2 (COX-2) in inflamed tissue and deregulates the protective immune response against TB and suppresses T cell mediated immunity (12). Co-investigator Taskén and colleagues have established the role of cyclic adenosine monophosphate (cAMP) and protein kinase A (PKA) – Csk – Lck inhibitory pathway as a general mechanism in chronic inflammation that inhibits T cell signaling and activation through antigen receptors on lymphoid cells (13,14). Peripherally induced Tregs express COX-2, secrete PGE2 and thus enhance cAMP levels within effector cells so this mechanism may also be involved in the inhibitory function of Tregs (15). Similarly, monocytes and macrophages stimulated with a number of bacterial inflammatory mediators potently induce T cell cAMP levels and T cell dysfunction (16). Reduction of cAMP levels in T cells by reducing production of PGE2 via COX-2 inhibitors (COX-2i) and thereby EP receptor stimuli to T cells can therefore reverse this process and restore T cell responsiveness to vaccine antigens as we have shown for HIV-infected patients (17-19). Our hypothesis is that inefficacy of cellular immunity in TB lesions is closely linked to the chronic inflammation and resulting cAMP-mediated activation defects in the T cells. Since human active TB infection is dominated by macrophage activation in granulomatous inflammation, it is relevant to study whether hyperexpressed PGE2 and COX-2 contribute to T cell dysfunction and the effect of COX-2i in active human TB.

1.2 Pre-Clinical & Clinical Experience with COX-2i and H56:IC31

1.2.1 Pre-clinical studies of COX-2i in experimental animal TB models

Animal models support PGE2 as a significant factor in the pathogenesis of a dysfunctional hyper-activated immune system in TB. Studies of experimental pulmonary TB in BALB/c mice have shown that during early infection bacilli are efficiently phagocytised by lung macrophages that secrete pro-inflammatory cytokines (TNF-α, IL-1) and accordingly present bacillary antigens to IL-12 and IFN-γ producing Th1 cells (12). During progressive disease Th2 cells emerge and an overproduction of anti-inflammatory and immunosuppressive molecules such as cortisol, PGE2, and transforming growth factor-beta (TGF-β) takes place that next downregulate cell-mediated immunity, enabling bacilli growth and progressive tissue damage. When PGE2 production was suppressed by COX-2i in animals with advanced disease, reductions in pulmonary inflammation and bacillary load took place in parallel with reversal to a Th1 cell profile (12,20). Further, the COX-2i celecoxib improved the 60-day survival of *Mtb*-infected mice to 100% with reduced lung levels of PGE2, bacillary load and inhibitory IL-10, whereas proinflammatory IL-12, IFN-γ and alveolar macrophage nitric oxide synthesis increased (21). Taken together inhibiting the COX-2 activity apparently improves outcome in active TB. A highly significant publication concludes that a human intervention study with anti-inflammatory drugs to TB patients receiving standard chemotherapy should be designed based on their murine data demonstrating reduced Mtb load and lung lesions as well as better survival of animals treated with ibuprofen (22).

1.2.2 Clinical studies of COX-2i

In three clinical explorative trials, we have demonstrated that COX-2i improves the immune functions of HIV patients on ART (17-19).) We have also showed for the first time that treatment with a COX-2i was able to downregulate chronic immune activation and improve T cell functions in asymptomatic HIV-infected patients who did not use antiretroviral therapy (19). In these patients, chronic immune activation was dampened as demonstrated; CD38 density on CD8+ T cells (primary endpoint) decreased by 24% by study week 12 and improvement of CD4+ T cell loss with 30 CD4 cells $\mu l^{-1} year^{-1.}$ Celecoxib further reduced the inhibitory surface receptor programmed death 1 (PD-1) on CD8+ T cells, improved humoral memory recall responses to a T cell-dependent vaccine and IL-6 tended to decrease in the treatment arm. In conclusion, celecoxib downmodulated the immune activation related to clinical progression of chronic HIV infection and improved T cell-dependent functions *in vivo*. There was an acceptable safety profile with celecoxib doses as high as 400 mg bid and no SAEs reported in any of the studies. Rash was observed in HIV patients without ART after receiving high-dose celecoxib in contrast to what we found with identical doses in patients on ART. Thus, it was concluded that this was associated by high HIV-induced immune activation levels itself and may have represented other bioprocesses than conventional drug allergies (19).

1.2.3 Pre-clinical studies of the H56:IC31 TB vaccine in animal TB models

H56 is a fusion protein of three mycobacterial antigens (Ag85B, ESAT-6, Rv2660c). Both Ag85B and ESAT-6 induce TB specific T cell responses that protect animals from *Mtb* challenge (23), whereas Rv2660c is preferentially recognized by latently infected individuals (24). The therapeutic post-exposure vaccine H56, developed at SSI, promotes a T cell response in mouse models with a high proportion of polyfunctional CD4 T cells that confers protective immunity and more efficient containment of late-stage infection than its predecessor the H1 (Ag85B-ESAT6) and BCG vaccines (23). This is also the first vaccine that demonstrates efficacy when administered post-exposure. H56 has been tested pre-clinically formulated with a range of adjuvants, most notably IC31® comprised of an oligodeoxynucleotide ODN1a complexed to a leucine and lysine containing polypeptide (25). The vaccine candidate combining H56 and IC31®, termed H56:IC (AERAS-456), seems to be immunogenic and protective also in non-human primates (26). However, some vaccinated non-human primates still demonstrate lack of H56:IC31 vaccine recall responses and experienced disease progression implying that strategies to boost vaccine responses should be tested.

1.2.4 Clinical studies of the H56:IC31 TB vaccine

Three phase I clinical trials and two phase II clinical trials of SSI's preceding and structurally related vaccine candidate H1 (Ag85B/ESAT-6):IC31® have been conducted in humans demonstrating good safety and tolerability.

The first phase I trial with H56:IC31, an open-label, dose-escalation clinical study (C-032-456) in latent TB has just been finalized and show that the vaccine is immunogenic and well tolerated. Further, in C-035-456, a phase I/IIa randomized, placebo-controlled study of HIV-negative adults, no safety issues have been identified. In these trials a total of 123 subjects have been vaccinated, 25 of whom have received up to three doses of H56:IC31. The dose of H56:IC31 in these trials ranges from 5 to 50 µg H56 with 500 nmol IC31. Concerning immunogenic efficacy for Ag85B, the most prominent CD4+ responses at Study Day 70 were polyfunctional (IFN-γ, IL-2, TNFα), bifunctional (IL-2 and TNFα), and monofunctional (IL-2). For ESAT-6, the most prominent CD4+ responses at Study Day 14 were polyfunctional (IFN-γ, IL-2, TNF), bifunctional (IFN-γ and TNFα), and monofunctional (IFN-γ or TNFα). CD4+ cells expressing IL-17 were rarely present after stimulation with either antigen Ag85B or ESAT-6.

Thus, H56:IC31 was associated with an acceptable safety profile in subjects with and without LTBI, and no vaccine-related serious adverse events (SAEs) were reported. Vaccination with H56:IC31 was associated most commonly (≥10% of subjects with a related adverse event) with mild to moderate injection site reactions of pain, warmth, and swelling; mild to moderate systemic adverse events of fatigue, bradycardia, myalgia, nausea, headache, arthralgia, and white blood cell count increased; and mild to severe hypertension. Only two SAE have been reported to date in clinical trials of H56:IC31, both considered not related to study vaccine. One serious AE, unrelated to study vaccine, was reported in the registry study. There are no reports of deaths due to vaccines containing similar fusion proteins (H1 and H4) to H56 or containing IC31 adjuvant.

A phase I/II clinical trial of H56:IC31 (C-037-456) in patients treated for active pulmonary TB has started in South-Africa in 2015, and no adverse events have been reported after the first 12 subjects have been vaccinated (by February 2015). The relationship between possible immune-related toxicities to the TB antigens and time since start of anti-TB chemotherapy will be addressed in C-037-456. The current study will await the inclusion of patients to the H56:IC31 arms until preliminary safety data from this trial is available.

In conclusion, all non-clinical and clinical information supports the suitability of H56:IC31 as a safe and immunogenic vaccine. There is no reason to believe that the combination of COX2i and vaccination is unfavorable.

1.3 Rationale for the Study and Purpose

1.3.1 TB therapeutic vaccines as rescue therapy in MDR-TB

Vaccination with Bacillus Calmette-Guérin (BCG) can limit disseminated disease in children, but not the establishment of TB infection. Several prophylactic TB vaccines have demonstrated superiority to BCG in mouse models, although none of them result in sterilizing immunity (27,28). Recently, the first novel preventive TB vaccine to enter efficacy testing (MVA85A) failed with no evidence of protection against TB disease or infection (29). These disappointing data has intensified the search for correlates of protection and to identify effective vaccine measures early during development. It has become clear that the traditional clinical development with consecutive clinical trials with a final efficacy trial needs rethinking. Thus, therapeutic vaccines have been listed as an alternative approach as the study design allows to measure the biological effect of the vaccine directly e.g. by measuring bacterial loads in sputum and also open up the possibility of identifying correlates of protection. Therapeutic vaccination alone or in combination with other types of immune modulation as COX-2i may not only boost and strengthen TB immunity with possibly better outcome especially for MDR/XDR-TB, but will also significantly improve on our knowledge relating to clinical study design and correlates of protection.

1.3.2 COX-2i improves cellular vaccine responses *in vivo*

Supported by the RCN/Global Health program we have shown for the first time in consecutive randomized clinical trials that COX-2i reduce T cell distress in HIV-1 and improve efficacy of T cell-dependent vaccines with good safety (17-19). In the study of celecoxib, antibody levels to the T cell-dependent tetanus vaccine were nearly three times higher in COX-2i-treated patients after vaccination than in controls (19). This supports our basic hypothesis that COX-2i treatment indeed can reverse T cell dysfunction in infectious diseases with a chronic inflammatory component. Furthermore, the data intriguingly suggest that COX-2i treatment could improve vaccine responses as well as improve T cell functions in general and particularly in patients with hyper-inflammatory lesions as in active TB.

1.3.3 COX-2i improves TB specific immune responses and regulation *in vitro*

Functional studies on the effect of COX-2i upon immune cells, longitudinally collected from clinically characterized TB patients during therapy at our hospital and from our national partners, are currently in progress in our lab. We have previously described elevated levels of Treg subsets in active and latent TB (10). In studies of active TB patients during anti-TB chemotherapy we demonstrate a decrease in the fraction of *Mtb*-specific IFN-γ-producing T cells stimulated by TB-specific antigens (ESAT-6, CFP-10) *in vitro* already after 2 weeks of efficient treatment, whereas the pool of IL-2+ cells increased over time (30). The Treg was still able to expand after *Mtb*-specific in vitro stimulation, but a decline in activated Treg was seen when COX-i was added to the cells. These pilot findings indicate that COX-2i could possibly block regulatory T cells and improve TB specific immunity. In the current study these data will form the basis for additional in-depth exploratory analysis of Tregs in TB infection.

1.3.4 Rationale for COX-2i regimen and dose selected

The immune modulatory effects of COX-2i are expected to be dose-dependent; hence in our previous exploratory trial of untreated HIV patients (UUSCOX2, EudraCT No 2006-001882-41, approved by the Norwegian Medicines Agency), the highest tolerated dose of celecoxib, 400 mg bid for 12 weeks (dose approved by FDA in familial adenomatous polyposis), were investigated and demonstrated improved efficacy of T cell-dependent vaccines with good safety (19). Further, we have recently ended another study of the COX-2i etoricoxib 90 mg OD given to a HIV cohort for 24 weeks (OUSCOX2. EudraCT No. 2010-020382-25) without any drug-related SAE observed.

Etoricoxib is generally well tolerated, even at doses above the clinical dose range and could be dosed once daily which is very convenient in order to secure compliance. Multiple dosing of etoricoxib up to 150 mg OD has showed no negative COX-1-mediated effects such as thromboxane B2, bleeding time or platelet aggregation (31). Steady-state pharmacokinetics of 120 mg etoricoxib, were consistent with linearity and achieved after 7 days of once-daily dosing in a study of 24 healthy subjects (32).

The COX-2i drug class has long been approved and is widely in use for inflammatory conditions. They are already in use in clinical practice for milder manifestations of immune reconstitution syndrome (IRIS), predominantly TB, in HIV-patients where they are preferred before steroids (31). The risk for cardiovascular events is low and the mechanism is still unclear as COX-2i lowers pro-inflammatory and prognostic markers of cardiovascular events in patients with CHD (32). In our last study, a number of protrombotic factors and markers for endothelial damage were measured, finding only a slight increase of D-dimer in the control group and possibly a reduction of the pro-athrogenic cytokine IL-6 in the intervention group (19). However, also in this trial we will implement a number of exclusion criteria and patients with typical high cardiovascular risk will be excluded and parameters reflecting activated coagulation and endothelial damage will again be monitored.

The anti-TB drug rifampicin that is included in the standard regimen for TB patients recruited to this study is a CYP3A inducer in the liver and will thus reduce the serum concentration of COX-2i. Co-administration of 120 mg etoricoxib with daily doses of 600 mg rifampin resulted in a mean 65% decrease in etoricoxib AUC and 40% decrease in C_{max} (33). The geometric mean ratios (etoricoxib

with rifampin/etoricoxib alone) for AUC (0-inf) and Cmax in these patients were: AUC: 0.35 (90% CI: (0.31, 0.40)), with a range of 0.25 - 0.50. Cmax: 0.60 (90% CI: (0.43, 0.84) with a range of 0.29 - 0.87). In the same individuals, the ranges of AUC's and Cmax's during rifampin treatment were as follows: AUC (0-inf) (ug*hr/mL): 2.41-6.42 with a mean of 3.80 and SD of 1.36. Cmax (ng/mL): 362-1616 with a mean of 733 and SD of 394. Tmax for etoricoxib was not significantly altered during rifampin treatment, but the apparent terminal t½ (hour) was significantly different (6.8 hours with rifampin versus 14.4 hours without).

Thus, we will use Arcoxia 120 mg, the maximal dose approved (and recommended for acute artritis urica) to overcome too low serum concentrations when co-administrated with rifampicin in TB patients. However, the serum concentration of rifampicin will not be affected and there are no interactions between COX-2i and any of the three other anti-TB drugs included in the standard regimen (isoniazide, ethambutol, pyrazinamide).

Analysis of serum-concentration of COX-2i is not routinely available and it is thus not possible to use for drug monitoring during the study. However, pharmacokinetic parameters in serum after 7 days of etoricoxib when steady state is expected (33) will be analyzed in study arm #1 in retrospect for research purposes and correlated to tolerance and immunogenicity and before inclusion of study arm #4 to achieve optimal dosing. If intolerable adverse reactions occur to etoricoxib the dose will be reduced to 90 mg or stopped.

2 STUDY OBJECTIVES AND RELATED ENDPOINTS

Primary:

To evaluate the ***safety*** and ***tolerability*** of etoricoxib alone and in combination with the therapeutic TB vaccine H56:IC31 in patients with active TB disease treated with conventional 26-week anti-TB chemotherapy.

Secondary:

To evaluate the effect of etoricoxib on i) immune regulation in TB and ii) immunogenicity to the therapeutic TB vaccine (H56:IC31) in patients with active TB disease treated with conventional 26-weeks anti-TB chemotherapy.

To investigate the pharmacokinetics of etoricoxib in TB patients treated with rifampicin.

3 OVERALL STUDY DESIGN

Patients admitted to the Dep. of Infectious Diseases or Dep. of Pulmonary Medicine, OUS (eventually patients admitted to other hospitals in the Oslo region and transferred to OUS or patients admitted to Haukeland University Hospital, Bergen) and with confirmed active pulmonary and/or extrapulmonary TB disease infected with drug-sensitive *Mtb* strains will be included sequentially in an open label, explorative, randomized, clinical phase I trial.

After Informed consent, inclusion, baseline assessments and before start of standard anti-TB chemotherapy (day 0-182), each patient will be randomized to the following study arms:

Supplementary Table 12

Group	Numbers	Treatment	Endpoint
Arm 1	10	Chemotherapy+ COX-2i	Safety + immunogenicity
Arm 2	10	Chemotherapy + H56:IC31	Safety + immunogenicity
Arm 3	10	Chemotherapy	Safety + immunogenicity (control)

Arm 4	10	Chemotherapy + H56:IC31 + COX-2i	Safety + immunogenicity

The study design randomizing patients will test:

1. Safety of etoricoxib and H56:IC31 alone and in combination in active TB.
2. Effect of etoricoxib on TB immunogenicity, activation and regulation (arm #1).
3. Pharmacokinetics of etoricoxib in TB patients treated with rifampicin (arm #1).
4. H56:IC31 immunogenicity after two immunizations in active TB (arm #2).
5. H56:IC31 immunogenicity after pre-treatment with etoricoxib in active TB (arm #4).

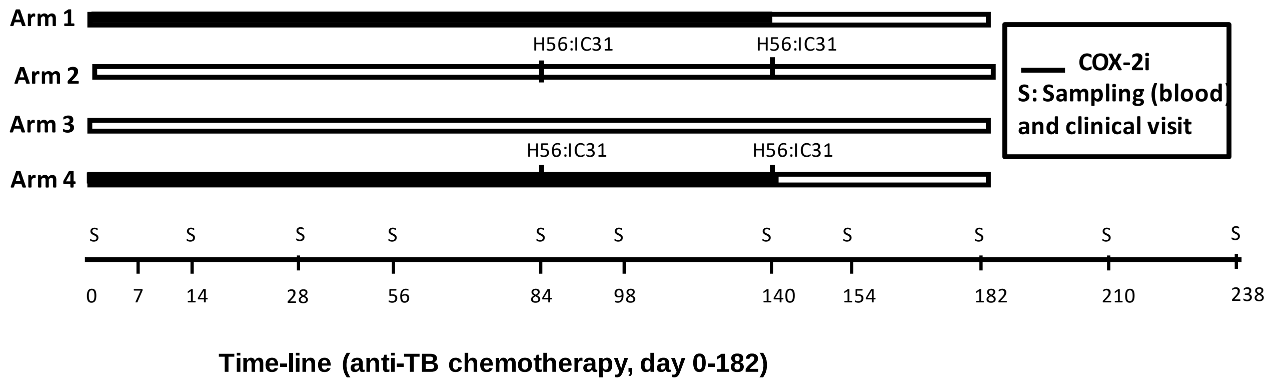

Supplementary Figure 9

The safety profile of COX2i or H56:IC31 has not yet been determined in a population undergoing treatment for pulmonary TB. Therefore, we will enrol participants in two groups separated by a safety review. Group A includes volunteers for arm #1, arm #2 and arm #3 (2:2:1 randomization). First at the completion of Study Day 98 for all group A participants, the “clinical investigational group” (sponsor, PI, collaborators including representative from the H56:IC31 vaccine manufacturer) will meet and evaluate the cumulative safety data to determine if the observed safety profile meets the criteria to proceed to inclusion of volunteers for group B; arm #4 and remaining Arm #3 controls (2:1 randomization).

Arm #1 (n=10): etoricoxib treatment started at day 0 and given for 20 weeks (day 0-140) followed by 6 weeks (or according to clinical evaluation) of standard anti-TB chemotherapy alone (day 0-182) and follow-up control at day 210 and day 238.

Arm #2 (n=10): given anti-TB chemotherapy alone (day 0-182) (or according to clinical evaluation) and follow-up control at day 210 and day 238. TB vaccine H56:IC31 will be injected at day 84 and day 140.

Arm #3 (controls, n=10): given anti-TB chemotherapy alone (day 0-182) (or according to clinical evaluation) and follow-up control at day 210 and day 238.

Arm #4 (n=10): etoricoxib treatment started at day 0 and given for 20 weeks (day 0-140) followed by 6 weeks (or according to clinical evaluation) of standard anti-TB chemotherapy alone and follow-up control at day 210 and day 238. TB vaccine H56:IC31 will be injected at day 84 and day 140.

4 STUDY POPULATION

4.1 Selection of Study Population

Patients admitted to the Dep. of Infectious Diseases and Dep. of Pulmonary Medicine, OUS and with confirmed active pulmonary and/or extrapulmonary TB disease infected with drug-sensitive *Mtb* strains will be included. If there are difficulties in the recruitment of patients at OUS, TB patients will be recruited in collaboration with other hospitals in the Oslo region or our partners from Haukeland University Hospital.

4.2 Number of Patients

A total of 40 patients will be included, 10 in each of the study arms.

4.3 Inclusion Criteria

Subjects may be included in the study if they meet all of the following criteria:

- Age between 18 - 70 years at the time of randomization
- Microbiologically confirmed pulmonary and/or extrapulmonary TB as defined in Table A1 (culture and/or PCR + susceptibility testing).
- Drug sensitive *Mtb* strains (except single resistance where fully adequate anti-TB chemotherapy regimen could be provided).
- Is willing and likely to comply with the trial procedures and is prepared to grant authorized persons access to their medical record.
- Has completed the written informed consent process prior to the start of screening evaluations.
- Females at childbearing age must agree to avoid pregnancy from screening and during the trial.

Female study participants at fertile age (not sterilized and still menstruating or within 1 year of the last menses if menopausal) will be informed about the risk of using the IMPs if pregnant or breastfeeding. The study investigator will perform an individual evaluation of each participant to secure acceptable methods of avoiding pregnancy during the study.

Acceptable methods of avoiding pregnancy include a sterile sexual partner, sexual abstinence (not engaging in sexual intercourse), progestogen-only hormonal contraception associated with inhibition of ovulation (oral, injection, transdermal patch, or implant), intrauterine device (IUD), or a combination of male condom with either cap, diaphragm or sponge with spermicide (“double barrier methods”).

Supplementary Table 13. Forms of extrapulmonal TB that can be included:

Bone/joint (including soft tissue affection/abscess)
Lymphadenitis
Pleuritis
Abdominal (peritoneal and intestinal)
Urogenital

4.4 Exclusion Criteria

Subjects must be excluded from participating in this study if they meet any of the following criteria:

4.4.1 Study-specific exclusion criteria

- Disseminated (miliary) TB.
- Evidence of a new acute illness that may compromise the safety of the subject in the trial on study day 0.
- History of autoimmune disease or immunosuppression.
- History or laboratory evidence of any possible immunodeficiency state.
- Anemia (< 9 g/100 mL)
- HIV sero-positivity.
- Chronic hepatitis B (HBs antigen positive) with increased liver transaminases (ASAT, ALAT).
- Chronic hepatitis C (HCV RNA positive).
- Concomitant or sporadic use of NSAID or corticosteroids (>2 times per week)
- Other immune modulating therapies including DMARDs.
- Total cholesterol >7 mmol/L.
- Hypertension >140/90 mm Hg (treated or untreated) or treated with >1 antihypertensive drug at any blood pressure.
- Cardiovascular events or stroke in parents, siblings or off-springs occurring <55 years of age
- Serum creatinine above reference levels (females >90 µmol/L; males >105 µmol/L), see reduced creatinine clearance in Section 4.4.2).
- Known diabetes mellitus type I or diabetes mellitus type II with HbA1c >7%.
- Pregnancy (documented S-hCG >5 IU/l for females at childbearing age).
- Breastfeeding.
- Known hypersensitivity for vaccines or vaccine adjuvants.

4.4.2 Exclusion criteria according to the SPC on Arcoxia from Norwegian Medicines Agency

- Known hypersensitivity for etoricoxib or etoricoxib tablet substances
- Known hypersensitivity for sulphonamides
- Active peptic ulcer or gastrointestinal haemorrhage
- Patients with history of asthma, acute rhinitis, nasal polyps, angioneurotic oedema, urticaria or other allergic reactions after taking acetyl salicylic acid or NSAID including COX-2i.
- Moderate/severe deranged liver function (Child-Pugh >7).

- Creatinine clearance <30 ml/min
- Inflammatory bowel disease
- Heart failure (NYHA II-IV)
- Established ischaemic heart disease, peripheral arteriosclerosis and/or cerebrovascular disease, including previous myocardial infarction, angina pectoris, unstable angina, PCI or coronary bypass, previous transitory ischemic attack or apoplexia/stroke.

4.5 Criteria for receiving H56:IC31 vaccine

Subjects may receive H56:IC31 vaccination (arm #2 and #4) if they meet the following criteria:

Pulmonary TB:

3. Sputum obtained prior to 1th immunization at day 84 must be *Mtb* negative evaluated by at least two consecutive AFS or PCR/GeneXpert at least 7 days apart.
4. Clinical improvement with normal vital signs (blood pressure, temperature and pulse) and reduced inflammatory blood parameters (CRP and ESR) compared to baseline.

Extrapulmonary TB:

- Documented reduction in the extent of TB disease at the infectious site(s) within day 84 evaluated by physical and/or radiological examination (table A2).
- Clinical improvement with normal vital signs (blood pressure, temperature and pulse), improvement of any TB related symptoms to Grade 1-3 (table A2), stable or increased body-weight and reduced inflammatory blood parameters (CRP, ESR and WBC counts) compared to baseline.

4.6 Withdrawal and Termination Criteria

In accordance with the Declaration of Helsinki, each subject is free to withdraw from the study at any time without further explanations. An investigator also has the right to withdraw subjects from the study in the event of inter-current illness, adverse events or other reasons concerning the health or well being of the subject, or in the case of lack of cooperation.

Should a subject decide to withdraw, or should the investigator decide to withdraw the subject, efforts will be made to complete and report the observations up to the time of withdrawal as thoroughly as possible. A complete final evaluation at the time of the subject's withdrawal should be made and an explanation given of why the subject is withdrawing (if approved by the subject) or being withdrawn from the study.

The reason, date and time for withdrawal will be noted in the Case Record Form (CRF). If the reason for withdrawal is a clinical adverse event or an abnormal laboratory test result, monitoring will continue until the outcome is evident. If possible, measurements and recordings should continue according to the protocol.

The specific event or test result(s) being the reason for withdrawal will be recorded in the CRF.

5 TREATMENT

For this study the following are defined as Investigational Medicinal Product(s) (IMP).

- **Arcoxia**® blister tablets 120 mg.
- **H56:IC31** TB vaccine 5 μg

5.1 Drug Identity, Supply and Storage.

5.1.1 Description of Arcoxia®

Contains the COX-2i etoricoxib. Manufacturer MSD.

Formulation of Arcoxia®: TABLETTER 120 mg: Each tablet contains: Etorikoksib 120 mg, laktosemonohydrat 5 mg, supportive compounds. Color compounds: Indigotin (E 132), jernoksid (E 172), titandioksid (E 171).

Arcoxia® Packaging and storage: The Investigational product will be bought by the Investigator as ordinary marketed product sealed in individual tablets and kept in stock.

5.1.2 Description of H56:IC31

H56:IC31 is a vaccine composed of the H56 antigen and the IC31 adjuvant.

Manufacturer of the H56 Antigen, IC31 Adjuvant and H56:IC31 vaccine
Name: Statens Serum Institut
Address: 5 Artillerivej
DK-2300 Copenhagen S, Denmark
Telephone: +45 3268 3268
FAX: +45 3268 3868

H56:IC31 Formulations
H56:IC31 5/500: 0.8 mL containing 5 μg/0.5 mL H56 antigen and 500 nmol/0.5 mL IC31 adjuvant. H56:IC31 is formulated in a buffer consisting of 4 mM Glycine, 8 mM Tris and 135 mM NaCl (the vaccine is formulated using 1 part H56 concentrated bulk formulated in 20 mM Glycine and 4 parts IC31 1.25 x concentration formulated in 10 mM Tris and 168.75 mM NaCl). H56:IC31 5/500 vaccine will be supplied as a final formulated vaccine.

H56:IC31 Packaging
The vaccine is provided in clear type I glass vials (Schott Glaskontor) with a 20 mm Flurotec Plus injection, chlorobutyl rubber stopper (West Pharmaceutical Services) with a 20 mm center tear-off aluminum seal (West Pharmaceutical Services). The H56:IC31 vial contains 0.8 mL of greyish/colorless final formulated vaccine with an extractable volume > 0.5 mL. The plastic tops are color coded.

H56:IC31 (Storage and Handling)
H56:IC31 vials will be stored at the hospital pharmacy at a temperature between +2°C and +8°C and particulate matter. Vials with visible particulates will be quarantined and the sponsor notified. Vaccine doses for administration will be drawn by the study nurse directly into a 1 to 3 mL polypropylene Luer Lok™ syringe (similar to Becton Dickinson #300-962 or #309-585) via a 21 to 25-gauge needle using aseptic technique. It is important to use a Luer-Lok™ needle connection or an integral needle to avoid vaccine spray exposure from accidental dislodgement of the needle. H56:IC31 will be administered by the study nurse immediately after being drawn into the syringe.

5.1.3 Description of placebo

No placebo for etoricoxib or H56:IC31 will be used.

5.2 Dosage and Drug Administration

The following treatment will be administered:

1. Arcoxia® 120 mg tablets to be administered once daily (120 mg x 1).
2. H56:IC31 vaccine adm. intramuscularly at 5 µg (1st dose day 84 and 2nd dose day 140).

5.3 Duration of Therapy

Arcoxia 120 mg OD will be given for 20 weeks (day 0-140) from initiation of standard anti-TB chemotherapy until the second dose of H56:IC31 vaccine has been given unless disease progression or unacceptable toxicity are encountered. Subjects may also discontinue protocol therapy in the following instances; intercurrent illness which would in the judgment of the investigator effect patient safety, the ability to deliver treatment or the primary study endpoints or request by the patient.

5.4 Clinical Monitoring

Regular visits performed by the clinical investigators and study nurse will include registration of compliance, adverse events, routine clinical follow-up and examinations (*Mtb* cultures/ microscopy/PCR/Gene Xpert, routine blood samples included biochemical parameters associated with organ toxicity, X-ray). S-hCG will be analysed in females to exclude pregnancy (section 6.2.2). Sampling of biobank for primary immunogenicity and exploratory studies according to time-points as stated in section 6.1 (table 1).

In addition, the study nurse will after individual evaluation keep in contact with the participants to secure compliance and safety issues. If there is any question of concern the participants will be seen at an extra visit within few days depending on the issue.

The study arms receiving H56:IC31 vaccine will be monitored for 60 minutes after administration of study vaccine to insure that no acute allergic events are in process and to see if a febrile response is developing. The participants will be examined frequently at time-points as stated in section 6.1 (table 2) to evaluate the physical manifestations of the immunization and with blood samples to assess the biochemical parameters associated with organ toxicity.

5.5 Drug dose Modifications

If intolerable AEs occur AND is judged to be possibly, probably, or definitely related to etoricoxib the dose will be reduced from Arcoxia® 120 mg OD to Arcoxia® 90 mg tablets OD or the drug stopped according to evaluation of the PI (see section 6.3.2).

5.6 Concomitant Medication

Concomitant medication includes prescribed and non-prescribed drugs or other treatments and any vaccines other than the study vaccine given to a subject within 28 days before and up to the end of the observation period. The name of the medication, start and stop dates (or ‘ongoing’), route of administration, and indication must be recorded on the Concomitant Medications eCRF. The indication recorded must correspond to a medical term/diagnosis recorded on the AE eCRF or to a pre-existing condition noted in the subject’s medical history, or be noted as prophylaxis, e.g., dietary supplement.

The patients will be treated with the following standard anti-TB drug combination therapy for drug-sensitive *Mtb* throughout the study (1-4 in the first phase for 2 months and 1-2 for a total of minimum 6 months) dosed according to weight:

1. Rifampicin 450-600 mg
2. Isoniazid 300 mg
3. Ethambutol 800-1600 mg
4. Pyrazinamide 1000-2000 mg

<u>The following medications are not permitted during the study in either arm</u>:

Other NSAIDs, ASA, prednisolone, DMARDS or other immune modulating therapies.

If the patient’s clinical or bacteriological situation is changed during the study, other therapy will be instituted according to the investigator’s evaluation and good clinical practice.

All concomitant medication (incl. “over-the-counter” drugs) used by the patient will be recorded in the patient’s file and CRF.

5.7 Drug Accountability

The investigator is responsible for ensuring that investigational product is correctly received and recorded, handled and stored and used in accordance with this protocol. All investigational product containers (opened, unopened, or empty) must be returned to the investigator after the study. A drug dispensing log must be prepared for each subject. At time-points as stated in section 6.1 (table 1) or at any other relevant time-point the following information must be entered into the drug dispensing log:

-date of visit

-number of tablets and containers given to the patient, including container number(s)

-number of containers returned from the patient, including individual container number(s) and whether they are unopened, opened or empty

-the number of tablets left in the containers which are opened, but not empty

-explanation of any discrepancies

-signature of the person distributing/collecting the container(s)

After completion of the study, the completed drug dispensing logs must be signed by the investigator.

5.8 Treatment Compliance

Compliance of both study drug and anti-TB chemotherapy will be calculated based on observations from the TB coordinators (responsible for standard DOT treatment given to the individual patient) and on the number of returned tablets in the container and pill organizer observed at every study visit.

5.9 Drug Labeling

Arcoxia® TABLETTER 120 mg and 90 mg will be labeled by the Oslo University Hospital Pharmacy Ullevål according to “Forskrift om klinisk utprøving av legemidler til mennesker av 30.10.2009 (FOR-2009-10-30-1321)”. The label will be affixed to the outside of the container in a language that is understood by the patient (Appendix 1 and 2)

The H56:IC31 vaccine vials will be labeled with primary labels (appendix 3) and the containers with secondary labels (appendix 4).

The IMPs labels will state the patient’s initials, study code, batch-number, storage conditions, expiry date (for Arcoxia already at the original label), name and contact information of the Principal Investigator and that the material is for clinical trial / investigational use only.

5.10 Subject Numbering and Randomization

The subjects will be included by consecutive subject numbers from 1 and upwards. Step-wise inclusion starting with Group A and proceeding with Group B if safety data are satisfactory and meet the criteria at Study Day 98 for all Group A participants.

- At inclusion day 0, the patients will be consecutively randomized (2:2:1) in groups according to study group descriptions; arm #1 on etoricoxib (n=10), arm #2 receiving H56:IC31 vaccine (n=10) and arm #3 controls (n=5).

- At inclusion day 0, the patients will be consecutively randomized (2:1) in groups according to study group descriptions; arm #4 on etoricoxib and receiving H56:IC31 vaccine (n=10 and remaining arm #3 controls (n=5).

Each subject is identified in the study by a unique subject number that is assigned when subject signs the Informed Consent Form. Once assigned the subject number cannot be reused for any other subject.

6 STUDY PROCEDURES

6.1 Supplementary Table 14. Trial flow chart

	Treatment period (etoricoxib)									Follow-up period			End of study
**Study day**	**Screen**	**0**	**7**	**14**	**28**	**56**	**84**	**98**	**140**	**154**	**182**	**210**	**238**
**Window (days)**		**NA**	**±2**	**±2**	**±7**	**±7**	**±7**	**±3**	**±7**	**±3**	**±7**	**±14**	**±14**
Informed consent	X												
Inclusion/exclusion evaluation	X												
Demographics/ medical history	X												
AFS/Culture/Xpert[1]	X	X		X*		X*	X		X		X		X
Radiology[2]	X					X					X		
TB chemotherapy[3]		X	X	X	X	X	X	X	X	X	X		
Physical Examination[4]	X	X	X	X	X	X	X	X	X	X	X	X	X
Vital signs[5]	X	X	X	X	X	X	X	X	X	X	X	X	X
Symptoms[10]	X	X	X	X	X	X	X	X	X	X	X	X	X
Treatment (etoricoxib)		X	X	X	X	X	X	X	X				
Haematology/clinical chemistry[6]	X	X	X	X	X	X	X	X	X	X	X	X	X
Pharmacokinetics[7]			X										
Adverse events			X	X	X	X	X	X	X	X	X	X	X
Con. Medication	X	X	X	X	X	X	X	X	X	X	X	X	X
**Vaccine adm. (T)[8]**							T		T				
Blood sampling for exploratory ssays[9]		X		X	X	X	X	X	X	X	X	X	X
Vaccine IgG[9]		X					X	X	X	X			X
RNA analysis[9]		X					X	X	X	X			X
PBMC ICS[9]		X					X	X	X	X			X

1). AFS, culture, microscopy, PCR/GeneXpert as part of routine clinical evaluation of TB. 2) Appropriate radiology (chest X-ray/CT/MRI/UL/PET) taken as part of clinical evaluation <1 month before entering the study. 3) Anti-TB chemotherapy: standard combination therapy, induction phase day 0-56, continuation phase day 56-182. 4) Physical examination by clinical investigator at every visit, the extent depending on the clinical situation. In the case of glandular TB, the gland(s) are measured. 5) Blood pressure, pulse, temperature. 6) Hb, Lpk, monocytes, lymphocytes,Tpk, ESR, CRP, SR, Na, K, Ca, creatinine, ASAT, ALAT, LDH, bilirubin, amylase, CK, cholesterol, glucose, gGT, urine acid, S-hCG) at every visit. QFT-TB at day 0 and 182. 7). Study

arm #1:AUC and Cmax after 7 days of etoricoxib when still in-patient at OUS. 8) H56:IC31 vaccine only to study arms #2 and #4. 9) see immunogenicity parameters in section 7.1.2. *). If Mtb detected in sputum either by AFS, culture or PCR, 2 additional sputum samples most be performed and proven negative before H56 immunisattion. 10) coughs, arthralgia, sweating, nausea/vomiting, diarrhea, headache, fatigue, myalgia, chestpain (and eventual additional symptoms) are recorded in 5 grades (1.not present; 2. no interference with activity; 3. some interference with activity; 4. significant: prevents daily activity, 5. ER visit or hospitalization)

Supplementary Table 15. Evaluation of treatment response in extrapulmonal TB

Criteria for response	Timepoint for evaluation
Subjective improvement of symptoms (grade 1-3)[1]	all visits
Reduction or within normal range of ESR, CRP, WBC counts	all visits
Stable or increased weight (compared to weight at diagnosis)	all visits
Focal improvement[2]	all visits
Radiological improvement[3]	day 1, 56, 182*
Clinicians evaluation of improvement[4]	all visits

[1]Symptoms recorded in 5 grades (1. not present; 2. no interference with activity; 3. some interference with activity; 4. significant: prevents daily activity, 5. ER visit or hospitalization),

[2]Local examination if appropriate, i.e. measuring (X x Y cm) enlarged lymph glands.

[3]Ultrasound, X-ray, computer tomography or magnetic resonance imaging depending on the manifestation.

[4]Overall conclusive clinical evaluation of all examinations performed (see 4.5).

*Additional time-points if progression of disease is suspected.

6.2 By Visit

Informed consent must be given voluntarily by each subject before any trial specific procedures are initiated. All eligibility criteria should be assessed together with relevant baseline parameters prior to study inclusion (inclusion/exclusion criteria).

The following examinations/evaluations will be done at screening:

Clinical status

Medical history (including disease history and corresponding treatment details), physical examination e.g. (general apperance, BCG scar, eyes, fauces, thyroid, heart, lung, abdomen, peripheral lymph node, basic CNS examination, peripheral circulation, skin, odema, ecco-cardiography) and vital signs (blood pressure, temperature, pulse, weight, hight, BMI).

Concomitant medication

All concomitant medication (incl. “over-the-counter” drugs) used by the subject within 28 days of treatment start must be recorded in the CRF.

Radiology

Chest X-ray or CT scan/PET scan of thorax or other relevant organs to determine extent and stage of TB disease performed no more than 4 weeks prior to inclusion in the study. Chest X-ray as a minimum will also be performed day 56 and 182 (after 2 months and at the end of anti-TB chemotherapy) according to clinical guidelines.

Laboratory analysis

Sputum for cultures, microscopy and PCR (i.e GeneXpert). Blood samples will be taken to determine parameters of inflammation, haematology, liver and kidney function (see 7.2.4). S-hCG will be analysed in females to check for pregnancy. QuantiFERON-TB will be obtained before inclusion and at day 182 at the end of anti-TB chemotherapy.

6.2.1 Before Treatment Starts

The resistance analyses of *Mtb* should be completed and only patients with sensitive strains should be included. The exception is single resistance where fully adequate anti-TB chemotherapy regimen could be provided. Haematology and biochemistry analysis should be available and evaluated according to exclusion criteria. Baseline blood samples for biobanking to assess immunogenicity and exploratory studies should be drawn.

6.2.2 During Treatment

Regular visits will include registration of AE in the CRF and secure vaccinations and sampling of plasma and PBMC according to protocol for exploratory immune analysis at time-points as stated in section 6.1 (Table 2) and 7.1.2 (Table 3). Standard clinical follow-up (sputum cultures/AFS, clinical examination, routine blood samples, X-ray) will be performed at regular time-points. S-hCG will be analysed in females in the etoricoxib treatment study arms at every visit to check for pregnancy and in the H56:IC31 vaccine arms day dag 56 and 98 in addition to urine-HCG day 84 and day 140 immidiately before immunisation.

Pharmacokinetic analysis (AUC, Cmax) with repetitive serum sampling will be performed during one day in the period between day 7-14 after initiation of etoricoxib when the patients still are in hospital (arm #1).

6.2.3 End of Treatment Visit

All study arms will have an "End of Treatment visit" taking place at day 140 when etoricoxib treatment will be stopped and/or last (2nd) H56:IC31 immunisation is given.

All study arms will also have a control at day 154 and day 182 (or at later time-point according to clinical evaluation when anti-TB chemotherapy will be stopped).

6.2.4 Withdrawal Visit

If the patient is withdrawn from the study before completing the study the patient will still be followed and receive standard anti-TB chemotherapy for 26 weeks or as long as needed as determined by the responsible clinician according to clinical guidelines.

6.2.5 After End of Treatment (Follow-up)

All study arms will also have final follow-up controls and blood sampling for immune analysis and chemistry according to protocol (Table 2) at day 210 and 238 (10 and 14 weeks after etoricoxib treatment is stopped and last H56:IC31 immunisation is performed (resp. 4 and 8 weeks after anti-TB chemotherapy is ended).

6.3 Criteria for Patient Discontinuation

6.3.1 Rules for Discontinuing the whole Study in an Individual Subject

Patients may be discontinued from study treatment and assessments at any time. Patient is also at any time free to discontinue his/her participation in the study, without any further explanation or prejudice to further treatment.

Specific reasons for discontinuing a patient for this study are:

- Safety reason as judged by the Principal Investigator
- Major protocol deviation
- Incorrect enrolment ie, the patient does not meet the required inclusion/exclusion criteria for the study
- Patient's non-compliance to study treatment and/or procedures
- Patient lost to follow-up
- A female patient becoming pregnant
- TB disease progression
- Development of autoimmune disease or immunosuppression
- Death
- Other

6.3.2 Specific Rules for Discontinuing etoricoxib in an Individual Subject

- An objective clinical or laboratory parameter change which meets Grade 3 or Grade 4 severity, as defined in the protocol toxicity table, AND is judged to be possibly, probably, or definitely related to etoricoxib.

- Deterioration in the patients condition which in the opinion of the Principal Investigator warrants study medication discontinuation (to be records as an AE or under Investigator Discretion).

6.3.3 Rules for Discontinuing Vaccine Injections in an Individual Subject

Administration of additional study injections will be discontinued for an individual subject if he/she has any of the following:

- An objective clinical or laboratory parameter change which meets Grade 3 or Grade 4 severity, as defined in the protocol toxicity table, AND is judged to be possibly, probably, or definitely related to study vaccine.

- Fever (oral temperature ≥39˚C) within 1 week following study injection which is associated with constitutional symptoms (myalgia, arthralgia, fatigue, headache, anorexia, hives, chills) AND is judged to be possibly, probably, or definitely related to study injection.

- Injection site reaction that involves severe induration (>10 cm, prevents daily activity or necrosis/ulceration), erythema (>10 cm or necrosis/ulceration/ exfoliative dermatitis), tenderness (significant discomfort at rest or study site visit or hospitalization) or pain (any use of narcotic pain reliever or prevents daily activity or study site visit or hospitalization).

- Any event that in the opinion of the principal investigator precludes administration of any further study injections.

6.4 Procedures for Discontinuation

6.4.1 Patient Discontinuation

Patients who withdraw or are withdrawn from the study, will stop further etoricoxib treatment and/or and not receive vaccine immunization. The patient will still be followed and receive standard anti-TB chemotherapy for 26 weeks or as long as needed as determined by the responsible clinician according to clinical guidelines.

If the 1st H56:IC31 injection is missed (day 84) due to other reasons than in 6.3.3, the 2nd injection (day 140) can still be given and the patient continue to be included in the study.

The reason for discontinuation will be recorded. Any significant AE will be followed-up until the outcome either is recovered or resolved.

Patients who withdraw or are withdrawn from the study within the first 98 days of the study will be replaced.

6.4.2 Trial Discontinuation

The whole trial may be discontinued at the discretion of the PI or the sponsor in the event of any of the following:

- Occurrence of AEs unknown to date in respect of their nature, severity and duration
- Medical or ethical reasons affecting the continued performance of the trial
- Difficulties in the recruitment of patients
- Cancellation of drug development

The sponsor and principal investigator will inform all investigators, the relevant Competent Authorities and Ethics Committees of the termination of the trial along with the reasons for such action. If the study is terminated early on grounds of safety, the Competent Authorities and Ethics Committees will be informed within 15 days.

6.5 Laboratory Tests

Blood samples will be drawn by venepuncture. Plasma, serum and PBMCs will be isolated, frozen and stored in the Department of Infectious Diseases, Oslo University Hospitals approved “Research Biobank Infectious Diseases.” Principles governing biomedical research in humans as stated in the Declaration of Helsinki will be followed. See Section 7 regarding the analysis to be performed

Routine sputum, tissue and blood samples as part of the clinical diagnosis and follow-up of TB will be collected, handled and analysed in accordance with hospital/laboratory standard procedures.

7 ASSESSMENTS

7.1 Immunogenicity and Immunology Analyses

A summary of immunologic assays to be performed on blood specimens is shown in Table 3. Listed blood volumes are estimates. Blood will be collected for all assays. Required assays will be performed on available specimens from all listed subjects and time points. Exploratory assays may be performed on specimens from certain subjects and time points.

7.1.1 Immunogenicity and Immune variables

7.1.1.1 Flow cytometry

Immune responses to *in vitro* stimulation of whole blood or PBMC with H56 antigenic peptide pools (Ag85B, ESAT-6, and Rv2660c) and with peptides representing non-vaccine Mtb-derived antigens (CFP10, TB10.4, PPD) will be analysed in all study arms and assessed by:

- Cytokine producing CD4+ and CD8+ T cells (intracellular cytokine staining; ICS).
- T cell proliferation (cfse)
- Tregulatory cells (CD45RA, CD25, FoxP3, PD1)
- Monocyte, T cell and Treg COX-2i expression
- Soluble cytokines and PGE2 levels in plasma and cell supernatants analyzed by multiplex and ELISA

7.1.1.2 ELISPOT

Immune responses to *in vitro* stimulation of whole blood or PBMC with H56 antigenic peptide pools (Ag85B, ESAT-6, and Rv2660c) and CFP10: IFN-γ/IL-2 spot-forming units (SFU) /10^6 PBMC pre- and post-vaccination and the effect of COX-2i will be determined.

7.1.1.3 Transcriptional (RNA) biomarker analysis

The relative upregulation of an expanded panel of genes of interest will be determined using dual-color Reverse-Transcriptase Multiple Ligation-dependent Probe-Amplification (dcRT-MLPA). In brief, total RNA is extracted from PAXgene blood collection tubes using the PAXgene Blood RNA kit (PreAnalytiX, DE) and relative expression is determined with dcRT-MLPA assay and analyzed on a 3730 capillary sequencer (Life Technologies, US).

7.1.1.4 Mycobacterial growth inhibition assays (MGIA):

Vaccine immunogenicity will be assessed using MGIA, an *in vitro* challenge model assesses the ability of immune cells to control growth of *Mtb* in a controlled system. PBMCs will be infected with log phase *Mtb* and subsequently, the degree of mycobacterial growth (BACTEC MGIT 960), pre- and post-vaccination and the effect of COX-2i will be determined. This assay will be performed in BSL3 facilities at OUS or with collaborating partners.

7.1.1.5 IgG and humoral immunity.

Antigen-specific IgG response to H56:IC31 will be determined in plasma samples by ELISA pre- and post-vaccination. This assay will be done at SSI in Denmark.

7.1.2 Supplementary Table 16: Summary of Immunology Specimens and Assays

Assay	Prority	Purpose of assay	Location	Study Days	Volume per time
ICS; whole blood/PBMC, (flow cytometry)	Required	Determine cellular immune response to study vaccine and TB	OUS, Norway	0, 84 98,140, 154, 238	5 mL
T cell proliferation PBMC, (flow cytometry)	Required	Determine cellular immunogenicity to study vaccine and TB	OUS, Norway	0, 84, 98,140, 154, 238	5 mL
IFN-γ/IL-2 PBMC (ELISPOT)	Required	Determine cellular immunogenicity to study vaccine and TB	OUS, Norway	0, 84, 98,140, 154, 238	5 mL
Vaccine IgG (ELISA)	Exploratory	Determine humoral immunogenicity to study vaccine	SSI, Denmark	0, 84, 98, 140, 154, 238	4 ml (plasma)
MGIA	Exploratory	Determine in a *in vitro* challenge model the ability of immune cells to control growth of *Mtb*	To be determined	0, 84, 98, 140, 154, 238	10ml
PaxGene for RNA analysis (transcriptional profiling)	Exploratory	Identify early gene signatures that can predict immune responses	To be determined	0, 84, 98, 140, 154	3 mL
PBMC (flow cytometry)	Exploratory	T cell and monocyte regulation, activation cytokine production and cox-2 expression	OUS, Norway	0, 14, 28, 56, 84, 98, 140, 154, 182, 210, 238	10 mL

7.1.3 Primary immunogenicity Endpoints

1. Etoricoxib effect of TB specific immunogenicity: T cell proliferation and cytokine responses (change between day 0 and 140)

2. H56:IC31 vaccine immunogenicity: T cell proliferation and cytokine responses 14 days post-immunization (day 98 and day 154 compared to day 84)

3. Etoricoxib effect of H56:IC31 vaccine immunogenicity: T cell proliferation and cytokine responses 14 days post-immunization compared to H56:IC31 alone.

7.1.4 Exploratory immune studies

Several functional immune assays will test time- and drug-dependent aspects of cellular immune responses to non-specific and specific stimuli. These tests will be carried out at the different partner sites with corresponding technologies (see 7.1.2, table 3).

7.2 Safety and Tolerability Assessments

Safety will be monitored by the assessments described below as well as the collection of AEs at every visit. Significant findings that are present prior to the signing of informed consent will be included in the relevant medical history/ current medical condition page of the CRF. For details on AE collection and reporting see Section 8.

See Flow chart in Section 6 (table 2) for assessment schedule.

The “clinical investigational group”; the sponsor (Dag Kvale), the PI (Anne Ma Dyrhol-Riise) and the clinical study investigators will review the safety data throughout the course of the study and report as stated in section 8.7.

7.2.1 Medical History

A summary of the patient’s relevant medical history will be recorded on the appropriate CRF page(s). The following information will be collected:

- Diagnosis of co-morbidities (i.e diabetes, kidney failure), co-infections (HIV, Hepatitis B and C), incident events (i.e gastric ulcer, allergy, asthma) and previous TB.

- Time, duration, treatment and outcome of the respective diagnosis.

7.2.2 Physical Examination

A qualified physician will conduct a physical examination including an examination of general appearance, skin, neck (including thyroid), fauces, lymphoid glandels, eyes, lungs, heart, abdomen, basic nervous system evaluation, peripheral circulation.

Any pathological physical examination finding after start of treatment that is classified by the investigator as a clinically significant worsening compared to previous exam will be considered an AE, documented on the subject’s CRF, and followed until the outcome is determined.

7.2.3 Vital Signs

To be measured: Body temperature, systolic/diastolic blood pressure, heart rate, weight.

Any vital sign value after start of treatment that is judged by the investigator as a clinically significant worsening compared to previous exam will be considered an AE, documented on the subject’s CRF, and followed until the outcome is determined.

7.2.4 Clinical Laboratory Evaluation

The routine laboratory analyses will be performed at the Central Laboratory at Oslo University Hospital.

Haematology assessments will include the following parameters: haemoglobin, leucocytes, monocytes, lymphocytes, platelets, ESR.

Blood chemistry assessments will include the following parameter: CRP, Na, K, Ca, creatinine, creatinine-clearance, ASAT, ALAT, LDH, bilirubin, amylase, CK, gammaGT, cholesterol, glucose, HbA1C, urine acid. Serum-hCG and urine-hCG for females.

Results from clinical laboratory tests obtained as part of the study will be reviewed by a medically qualified study team member and treated in accordance with site policies.

Any laboratory value that is judged by the investigator as a clinically significant worsening compared to previous exam or has increased in toxicity grade after start of treatment with IMPs (see Appendix for toxicity grading scales) must be reported as an AE, documented on the subject's CRF, and followed until the outcome is determined.

The signed and interpreted laboratory results will be kept together with the subject's CRF as supplemental pages.

7.2.5 Pharmacokinetics

The AUC and Cmax of etoricoxib at steady state after 7 days of treatment will be analyzed in Arm #1 in order to study the degree of interaction between etoricoxib and rifampicin. Blood samples will be collected in heparinized tubes at regular intervals during 96 hours as previously described (32). The blood samples will be protected from light and stored on ice until the plasma could be separated. The separated plasma was stored at –20°C until analysis for etoricoxib.

7.2.6 Radiology

Routine chest X-ray or CT/PET scan will be performed at Dep. of Radiology, Oslo University Hospital before initiation of the study and at least at day 56 and day 182 and in addition according to clinical evaluation.

7.2.7 Adverse Events (AEs)

The adverse drug reaction (ADR) profiles for etoricoxib give reason to expect gastrointestinal AEs, especially nausea, as the most common (see section 8.2). Headache may also occur. In addition, study personnel must remain vigilant for the occurrence of any event related to the patients' TB diagnosis and anti-TB chemotherapy where gastrointestinal ADRs are the most common as well as to all other types of AEs.

8 SAFETY MONITORING AND REPORTING

The investigator is responsible for the detection and documentation of events meeting the criteria and definition of an adverse event (AE) or serious adverse event (SAE). Each patient will be instructed to contact the investigator immediately should they manifest any signs or symptoms they perceive as serious.

The methods for collection of safety data are described below.

8.1 Definitions

8.1.1 Adverse Event (AE)

An AE is any untoward medical occurrence in a patient administered a pharmaceutical product and which does not necessarily have a causal relationship with this treatment.

An adverse event (AE) can therefore be any unfavorable and unintended sign (including an abnormal laboratory finding), symptom, or disease temporally associated with the use of a medicinal (investigational) product, whether or not related to the medicinal (investigational) product.

The term AE is used to include both serious and non-serious AEs.

If an abnormal laboratory value/vital sign are associated with clinical signs and symptoms, the sign/symptom should be reported as an AE and the associated laboratory result/vital sign should be considered additional information that must be collected on the relevant CRF.

All conditions that exist prior to administration of the study vaccine (pre-existing conditions) will be recorded in the subject's medical history to establish baseline. Day-to-day fluctuations in pre-existing conditions that do not represent a clinically significant change in the subject's status will not necessarily be reported as AE.

Any adverse change from the subject's baseline condition that occurs following the administration of the IMPs will be considered an AE. This includes the occurrence of a new AE or the worsening of a baseline condition, whether or not considered related to the IMPs. Intermittent conditions such as headaches may be present on Study Day 0 but may represent an AE if the intensity or duration of the event is worse than usual following receipt of IMPs. AE include, but are not limited to: adverse changes from baseline that represent increases in toxicity grade according to the Toxicity Table adverse changes in the general condition of the subject, signs and symptoms noted by the subject, concomitant disease with onset or increased severity and changes in laboratory safety parameters occurring after administration of IMPs.

8.1.2 Serious Adverse Event (SAE)

Any untoward medical occurrence that at any dose:

- Results in death
- Is immediately life-threatening
- Requires in-patient hospitalization or prolongation of existing hospitalization
- Results in persistent or significant disability or incapacity
- Is a congenital abnormality or birth defect
- Is an important medical event that may jeopardize the subject or may require medical intervention to prevent one of the outcomes listed above.

Medical and scientific judgment is to be exercised in deciding on the seriousness of a case. Important medical events may not be immediately life-threatening or result in death or hospitalization, but may jeopardize the subject or may require intervention to prevent one of the listed outcomes in the definitions above. In such situations, or in doubtful cases, the case should be considered as serious. Hospitalization for administrative reason (for observation or social reasons) is allowed at the investigator's discretion and will not qualify as serious unless there is an associated adverse event warranting hospitalization.

8.1.3 Suspected Unexpected Serious Adverse Reaction (SUSAR)

Adverse Reaction: all untoward and unintended responses to an investigational medicinal product related to any dose administered;

Unexpected Adverse Reaction: an adverse reaction, the nature or severity of which is not consistent with the applicable product information.

Suspected Unexpected Serious Adverse Reaction: SAE (see section 8.1.2) that is unexpected as defined in section 8.2 and possibly related to the investigational medicinal product(s).

8.2 Expected Adverse Events (EAE)

Etoricoxib: Gastrointestinal EAE and headache are the most commonly occurring. ≥1/100 til <1/10: abdominal pain, constipation, gastritis, reflux, gas, diarréa, dyspeptic discomfort, nausea, vomiting, eosophagitis, palpitations, arythmia, hypertention, alveolar osteitis, increase in liver enzymes (ALAT/ASAT), bronchospasm, dizziness, headache, oedema, fatigue (text from Arcoxia, MSD, ATC-nr.: M01A H05 in Felleskatalogen). The EAEs will be recorded in the CRF.

H56:IC31: Vaccination with H56:IC31 has been associated most commonly (≥10% of subjects with one or several of the AE) with mild to moderate injection site reactions of pain, warmth, and swelling, mild to moderate systemic adverse events of fatigue, bradycardia, myalgia, nausea, headache, arthralgia, and white blood cell count increased; and mild to severe hypertension (Table 5-8, page 72, Statens Serum Institut Investigator's Brochure).

There have been no suspected unexpected serious adverse reactions (SUSARs) reported to date in clinical trials of H56:IC31.

8.3 Disease Progression/Recurrence

Progression of TB disease during the study is not expected since only patients infected with drug sensitive *Mtb* isolates given appropriate anti-TB chemotherapy are included.

It is expected that some few patients could experience initial worsening of symptoms due to the nature of the TB disease. Events definitely explained as caused by TB, will not be reported as an AE/SAE. However, if the investigator considers that there is a causal relationship between the IMPs and the disease progression or increase in symptoms then this must be reported as an SAE.

Death due to progressive disease is to be recorded on a specific from in the CRF, but not as an SAE.

8.4 Time Period for Reporting AE and SAE

For each patient the standard time period for collecting and recording AE and SAEs will begin at baseline (day 0) when anti-TB chemotherapy and study drug etoricoxib are started and continue until day 238 (14 weeks after the last dose of etoricoxib and last H56:IC31 immunization is given).

For H56:IC31 specific AEs will be reported through 14 days after each vaccination, injection site assessments performed through 14 days after each vaccination and SAE and SUSARs reported until end of study. The monitor will review unblinded safety data and make a formal recommendation on the continued conduct of the trial after each safety review.

During the course of the study all AEs and SAEs will be proactively followed up for each patient; events should be followed up to resolution, unless the event is considered by the investigator to be unlikely to resolve due to the underlying disease. Every effort should be made to obtain a resolution for all events, even if the events continue after discontinuation/study completion.

8.5 Recording of Adverse Events

If the patient has experienced or hospital personnel have observed AEs the investigator will record the following information in the CRF:

Date and time of onset and resolution (duration), severity (defined below), the nature of the event described by the investigator in precise standard medical terminology (MedDRA Version 18.0), whether it required treatment or action taken, outcome, relationship to the patient's TB diagnosis or treatment and whether the AE caused withdrawal from the study.

The ***severity*** of all AE, including clinical findings and abnormal laboratory values will be classified as one of the following grades using the following definitions:

1) Mild: Tolerable
2) Moderate: Interferes with normal activity
3) Severe: Incapacitating (causes inability to perform usual activity or work).

In addition, a Toxicity Table (*US FDA guidance: Toxicity Grading Scale for Healthy Adult and Adolescent Subjects Enrolled in Preventive Vaccine Clinical Trials, September 2007)* is provided in the protocol appendix for the grading of severity of specified AE. For AE not listed in the Toxicity Table determination of severity requires some level of interpretation as outlined above. The degree of incapacity caused by the AE and the level of medical intervention required for treatment may be helpful in assessing the overall severity of the AE.

The ***causal relationship*** of the event to the study medication will be assessed as one of the following:

Unrelated:

There is not a temporal relationship to investigational product administration (too early, or late, or investigational product not taken), or there is a reasonable causal relationship between non-investigational product, concurrent disease, or circumstance and the AE.

Unlikely:

There is a temporal relationship to investigational product administration, but there is not a reasonable causal relationship between the investigational product and the AE.

Possible:

There is reasonable causal relationship between the investigational product and the AE. Dechallenge information is lacking or unclear.

Probable:

There is a reasonable causal relationship between the investigational product and the AE. The event responds to dechallenge. Rechallenge is not required.

Definite:

There is a reasonable causal relationship between the investigational product and the AE.

The ***outcome*** of all adverse events will be classified as one of the following:

1. On-going
2. Resolved
3. Resolved with sequelae
4. Death
5. Unknown

8.6 Solicited Adverse Events and Injection Site Reactions

Solicited AE are events the subject is specifically asked about. These AE are commonly observed soon after receipt of vaccines. For this study, solicited AE to be collected include:

Local: injection site pain, injection site erythema, injection site swelling

Systemic: fatigue, myalgia, arthralgia, pyrexia, chills, nausea.

8.7 Reporting Procedure

8.7.1 AEs and SAEs

All adverse events and serious adverse events that should be reported as defined in section 8.1.1 will be recorded in the patient's CRF.

SAEs must be reported by the investigator to the sponsor, the medical expert Dag Kvale, either directly or by phone, within 24 hours after the site has gained knowledge of the SAE. Every SAE must be documented by the investigator on the SAE pages of the CRF. The Serious Adverse Event Report Form must be completed and signed in the eCRF. The medical expert will review the report in the eCRF. The initial report shall promptly be followed by detailed, written reports if necessary. The initial and follow-up reports shall identify the trial subjects by unique code numbers assigned to the latter.

All SAE reports will be collected and communicated annually to the National Regulatory Authority (Norwegian Medicines Agency). SAEs will be recorded if they occurred as follows:

- between the first administration of the study drug and the completion of the last follow-up evaluation, whether or not considered related to the investigational product.
- at any time after completion of the last follow-up evaluation, and came to the investigator's attention and were judged to be related to the subject's participation in the study.

8.7.2 SUSARs

SUSARs will be reported to the Norwegian Medicines Agency according to national regulation ("Forskrift om klinisk utprøving av legemidler til mennesker av 30.10.2009 (FOR-2009-10-30-1321)"). The following timelines should be followed:

The sponsor (Dag Kvale) will ensure that all relevant information about suspected serious unexpected adverse reactions that are fatal or life-threatening is recorded and reported as soon as possible to the Norwegian Medicines Agency in any case no later than seven (7) days after knowledge by the sponsor of such a case, and that relevant follow-up information is subsequently communicated within an additional eight (8) days.

All other suspected serious unexpected adverse reactions will be reported to the Competent Authority concerned as soon as possible but within a maximum of fifteen (15) days of first knowledge by the sponsor. SUSARs will be reported using in “paper form” or by the the CIOMS form since OUS is not connected to EudraVigilance.

Both SAEs and SUSARs related to the H56:IC31 will be reported to SSI within the same deadlines by cryptic e-mail clin.pv@ssi.dk or fax.nr. +45 32 68 82 47.

8.7.3 Annual Safety Report

Once a year throughout the clinical trial, the sponsor will provide the Competent Authority with an annual safety report. The format will comply with national requirements.

8.7.4 Clinical Study Report

The adverse events and serious adverse events occurring during the study will be discussed in the safety evaluation part of the Clinical Study Report.

8.8 Procedures in Case of Emergency

The investigator is responsible for assuring that there are procedures and expertise available to cope with emergencies during the study. The study will take place at the outpatient clinic at Dep. of Infectious Diseases, Oslo University Hospital where emergencies can be taken care of during H56:IC31 immunization. When the patient is home he will be given information about telephone numbers to phone in case of emergencies.

9 DATA MANAGEMENT AND MONITORING

9.1 Case Report Forms (CRFs)

The investigator will keep a screening log, recording all subjects who were screened and whether they were enrolled or not. A separate Subject Identification List will be kept, showing code numbers, names, and dates of birth to allow unambiguous identification of each subject included in the study.

The designated investigator staff will enter the data required by the protocol into the electronic Case report forms (CRF). The Principal Investigator is responsible for assuring that data entered into the CRF is complete, accurate, and that entry is performed in a timely manner. The signature of the investigator will attest the accuracy of the data on each CRF. If any assessments are omitted, the reason for such omissions will be noted on the CRFs. Corrections, with the reason for the corrections will also be recorded.

The principal investigator will sign the last page of the CRF. Any corrections to the data will be made in a manner that does not obscure the original entry and will be dated and initialed by the investigator or assigned designee

9.2 Source Data

The medical records for each patient should contain information important for the patient’s safety and continued care, and to fulfil the requirement that critical study data should be verifiable.

To achieve this, the medical records of each patient should clearly describe at least:

- That the patient is participating in the study, e.g. by including the enrollment number and the study code or other study identification;

- Date when Informed Consent was obtained from the patient and statement that patient received a copy of the signed and dated Informed Consent;
- Results of all assessments confirming a patient's eligibility for the study;
- Diseases (past and current; both the disease studied and others, as relevant);
- Surgical history, as relevant;
- Treatments withdrawn/withheld due to participation in the study;
- Results of assessments performed during the study;
- Treatments given, changes in treatments during the study and the time points for the changes;
- Visits to the clinic / telephone contacts during the study, including those for study purposes only;
- Non-Serious Adverse Events and Serious Adverse Events (if any) including causality assessments;
- Date of, and reason for, discontinuation from study treatment;
- Date of, and reason for, withdrawal from study;
- Date of death and cause of death, if available;
- Additional information according to local regulations and practice.

9.3 Study Monitoring

The investigator will be visited on a regular basis by the Clinical Study Monitor, who will check the following:

- Informed consent process
- Reporting of adverse events and all other safety data
- Adherence to protocol
- Maintenance of required regulatory documents
- Study Supply accountability
- Facilities and equipment's
- Data completion on the CRFs including source data verification (SDV)

The monitor will review the relevant CRFs for accuracy and completeness and will ask the site staff to adjust any discrepancies as required.

When the responsible study monitor has checked and verified the CRFs, the unidentified data (verified by study code) will be entered into a computer database at the approved scientific server at OUS for further handling and statistical evaluation. The code key will be stored at another approved server.

Sponsor's representatives (e.g. monitors, auditors) and/or competent authorities will be allowed access to source data for source data verification in which case a review of those parts of the hospital records relevant to the study may be required.

9.4 Confidentiality and database management

All information concerning the study will be stored in a safe place inaccessible to unauthorized personnel. The entry of data and coding will be secured by two study nurses. The following information will be retained at the investigator's site for at least 15 years after final patient visit according to "Forskrift om klinisk utprøving av legemidler til mennesker av 30.10.2009 (FOR-2009-10-30-1321)".

- Source documents
- CRFs
- Copies of protocols
- Protocol amendments
- Drug accountability forms
- Correspondence
- Subject identification lists
- Informed consent forms
- Copies of study reports

10 STATISTICAL METHODS AND DATA ANALYSIS

10.1 Determination of Sample Size

This is a first of its kind exploratory open label phase I study. Primary efficacy data of COX-2i as well as the H56:IC31 vaccine in patients with active TB are non-existing. Further, it is not optimal to extrapolate efficacy data from H56:IC31 studies of latent TB patients. Thus, a sample size calculation is therefore not performed. Still, H56:IC31 studies have shown significant differences in vaccine immunogenicity with 10 patients in each arm and we have proven differences in immune activation (CD38) in a HIV cohort after 12 weeks on high dose celecoxib (Pettersen et al., 2010) with 13 and 14 patients in the study and control groups, respectively.

10.2 Randomization

We will include 10 patients in each of the three study arms and 10 in the control arm. By open label randomization TB patients meeting the inclusion criteria will be included in a 2:2:1 fashion in the arms #1, #2 and #3. When the last patient have reached study day 98 (end of etoricoxib treatment), arms #4 and rest of controls arm #3 will be recruited in a 2:1 fashion if safety issue are satisfactory in arms #1, #2 and #3.

10.3 Population for Analysis

A table will be provided with the following information:

- number of subjects, both screened and enrolled, included in the study
- number of subjects included in the immunogenicity analysis

- number of subjects included in the safety analysis
- number of subjects withdrawn from the study and the reason for withdrawal

The following populations will be considered for the analyses:

- Intention to treat (ITT) population: All randomized participants, regardless of protocol adherence.
- Per-protocol population (PP): Includes all subjects who have received at least one dose of etoricoxib and/or H56:IC31.
- Safety population: Includes all subjects who have received at least one dose of etoricoxib and/or H56:IC31. Subjects who withdraw from the study will be included in the safety analysis. A list of withdrawn subjects, preferably with the reasons for withdrawal, will be made.

10.4 Statistical Analysis

Groups will be compared with non-parametrical statistical methods throughout with two-ways tests using p-values equal to or below 0.05 as significant. No adjustments due to multiple comparisons will be performed.

Deviation from the original statistical plan will be described and justified in the Clinical Study Report. The investigators can make the decision regarding individual values belonging to a subject to be excluded from the statistical evaluations, but only when a protocol violation is considered to weaken any of the scientific aspects of the study. Missing data will not be substituted by estimated values, but will be treated as missing data by the method used.

10.4.1 Safety analysis

The number and percentages of patients with at least one AE will be tabulated. Occurrence of particular AEs and their severity and relationship to study drug will be summarized.

10.4.2 Vital Signs

Categorical data will be summarized by treatment and as a total using count and percentages of patients. Continuous data will be summarized by treatment and as a total using medians, quartiles and ranges.

10.4.3 Physical Examinations

Categorical data will be summarized by treatment and as a total using count and percentages of patients. Continuous data will be summarized by treatment and as a total using median, quartiles and ranges.

10.4.4 Clinical Laboratory Measurements

Categorical data will be summarized by treatment and as a total using count and percentages of patients. Continuous data will be summarized by treatment and as a total using median, quartiles and ranges.

10.4.5 Pharmacokinetics

The AUC and Cmax of etoricoxib at steady state after 7 days of treatment will be analyzed and statistics performed as previously described (32).

10.4.6 Immunogenicity and Other Immunology Analyses

All immunogenicity analyses will be based on subjects who has received etoricoxib until the sample time-point in question and/or received at least one dose of study vaccine H56:IC31. Immunogenicity will be summarized for all time points as collected and as available. No imputation for missing data will be performed. Data will be transformed as appropriate prior to analysis.

10.4.6.1 Primary Immunogenicity endpoints

The primary variables of interest for assessment of immune response to vaccine will be the percentage of CD4+ and CD8+ T cells that produce any of selected cytokines. The immunogenicity of H56:IC31 in this study will be evaluated by PBMC ICS, T-cell proliferation and IL-2/IFN-γ ELISPOT. Due to the exploratory nature of immunogenicity endpoints, the primary evaluation will be based on descriptive summaries and no formal hypothesis testing will be performed.

PBMC/whole blood ICS Assay

Assessment of immune response by PBMC or whole blood ICS will be based on the percentage of cytokine producing CD4+ and CD8+ T cells in response to stimulation with H56 protein antigen and antigenic peptide pools (Ag85B, ESAT-6, Rv2660c, CFP-10). ICS parameters will include cytokines such as IFN-γ, IL-2 and TNF-α, and may include other cytokines to identify T cells of specific functionality. Additional cell surface markers, cytokines, or functional markers may also be analysed. Median control-subtracted cytokine responses and associated 95% confidence intervals (CIs) or other descriptive statistics as appropriate will be used to summarize the percentage of antigen-specific CD4 and CD8 T cell responses by all study group and treatment assignment at all available time points. Summaries of T cell responses will be presented by T cell type (CD4 and CD8), by stimulation antigen(s), and by cytokine profile. Summaries will include immune responses at all available pre- and post- etoricoxib and pre- and post-vaccination time points. Exploratory assessment of antigen specific CD4+ and CD8+ T cell responses in relation to the magnitude of QFT positivity pre and post-study may also be performed.

T cell proliferation

Assessment of T cell proliferation will be based on the percentage of CD4+ and CD8+ T cells expressing the proliferation marker cfse in response to stimulation with H56 protein antigen and antigenic peptide pools (Ag85B, ESAT-6, Rv2660c, CFP-10). Fraction of cell subsets and responses and associated 95% confidence intervals (CIs) or other descriptive statistics as appropriate will be used to summarize the percentage of antigen-specific CD4+ and CD8+ T cell responses by all study group and treatment assignment at all available time points. Summaries of T cell responses will be presented by T cell type (CD4 and CD8) and by stimulation antigen(s) at all available pre- and post- etoricoxib and pre- and post-vaccination time-points.

ELISPOT

Assessment of immune response by ELISPOT will be based on the number of IL-2/IFN-γ spot-forming units (SFU) per 10^6 PBMC in response to stimulation with H56 antigen and antigenic peptide pools (Ag85B, ESAT-6, Rv2660c, CFP-10). Geometric mean and associated 95% CIs will be used to summarize the number of IL-2/IFN-γ SFU per 10^6 PBMC by study group and treatment assignment at all available time points. Summaries of the number of IL-2/IFN-γ SFU per 10^6 PBMC will be presented by stimulation antigen(s) and will include immune response at all available pre- and post- etoricoxib and pre- and post-vaccination time-points.

QuantiFERON-TB Gold Test

Results for the QuantiFERON-TB Gold test at baseline day 0 and day 182 will be summarized using subject count (percentage) summaries and descriptive statistics.

10.4.6.2 Exploratory Assays

dcRT-MLPA (RNA Analysis)

As an exploratory endpoint for examining RNA gene expression signatures, RNA expression will be performed. RNA will be isolated from whole blood. Signatures of gene expression changes after vaccination may be determined using computational systems biology tools.

Antibody IgG ELISA

Geometric mean titers for antibody ELISA to Ag85B, ESAT-6, and Rv2660c and associated 95% CIs will be summarized at all pre- and post-vaccination time-points.

PBMC flow cytometry Assay

Assessment of immune phenotyping and responses by PBMC will be based on the percentage of CD4+ and CD8+ T cells and monocytes expressing various markers of activation, regulation, maturity and COX-2 *ex vivo* and in response to stimulation with H56 protein antigen and antigenic peptide pools (Ag85B, ESAT-6, Rv2660c) and eventually with peptides representing non-vaccine *Mtb*-derived antigens (TB10.4, CFP10, PPD). Fraction of cell subsets and responses and associated 95% confidence intervals (CIs) or other descriptive statistics as appropriate will be used to summarize the percentage of antigen-specific CD4+ and CD8+ T cell responses by all study group and treatment assignment at all available time points. Summaries of T cell responses will be presented by T cell type (CD4 and CD8), by stimulation antigen(s), and by marker profile at all available pre- and post-etoricoxib and pre-and post-vaccination time-points.

11 STUDY MANAGEMENT

11.1 Investigator Delegation Procedure

The principal investigator is responsible for making and updating a “delegation of tasks” listing all the involved co-workers and their role in the project. He will ensure that appropriate training relevant to the study is given to all of these staff, and that any new information of relevance to the performance of this study is forwarded to the staff involved.

11.2 Investigator’s Responsibilities

The investigator(s) is responsible for performing the study in accordance with this protocol and the ICH guidelines on GCP, and for collecting, recording, and reporting the data accurately and properly. Agreement of the investigator to conduct and administer this study in accordance with the protocol will be documented in a separate study agreement.

The investigator has the overall responsibility for the conduct and administration of the study at the centre, and for contacts with study centre management, the EC and with local authorities.

The investigator is responsible for giving information about the study to all staff members involved in the study or in any element of subject management, both before starting the practical performance of the study and during the course of the study (e.g., when new staff become involved).

The investigator(s) is responsible for ensuring the privacy, health, and welfare of the subjects during and after the study. The investigator must be familiar with the background and requirements of the study and with the properties of the study drugs as described in the package insert.

Authorised personnel of the Regulatory Authority inspector(s) or their agents will be given direct access to source data and documentation (e.g., medical charts/records, laboratory results, printouts, etc.) for source data verification, provided that subject confidentiality is maintained.

11.3 Protocol Adherence

Investigators ascertain they will apply due diligence to avoid protocol deviations.
All significant protocol deviations will be recorded and reported in the Clinical Study Report (CSR).

11.4 Study Amendments

If it is necessary for the study protocol to be amended, the amendment and/or a new version of the study protocol (Amended Protocol) must be notified to and approved by the Competent Authority and the Ethics Committee according to EU and national regulations.

A Toxicity Table (*US FDA guidance: Toxicity Grading Scale for Healthy Adult and Adolescent Subjects Enrolled in Preventive Vaccine Clinical Trials, September 2007*) will be amended. No other substantial changes from the final approved (signed) protocol will be initiated without the EC's and the Regulatory Authority's prior written approval or favorable opinion of a written amendment, except when necessary to eliminate immediate hazards to the subjects or when the change involves only logistics or administration. The investigator will sign the protocol amendment. Any significant deviation from the protocol when no approved amendment exists will be regarded as a protocol violation, and will be addressed as such during the reporting of the study.

Before any subjects are enrolled in the study, a list of potential protocol violations and deviations will be created, with corresponding actions to be taken.

11.5 Audit and Inspections

Authorized representatives of a Competent Authority and Ethics Committee may visit the centre to perform inspections, including source data verification. Likewise, the representatives from sponsor may visit the centre to perform an audit. The purpose of an audit or inspection is to systematically and independently examine all study-related activities and documents to determine whether these activities were conducted, and data were recorded, analyzed, and accurately reported according to the protocol, Good Clinical Practice (ICH GCP), and any applicable regulatory requirements. The principal investigator will ensure that the inspectors and auditors will be provided with access to source data/documents. The investigators may engage an internal or external monitor.

12 ETHICAL AND REGULATORY REQUIREMENTS

The study will be conducted in accordance with ethical principles that have their origin in the Declaration of Helsinki and are consistent with *International Conference on Harmonisation (ICH)/Good Clinical Practice (GCP)* and applicable regulatory requirements. Registration of patient data will be carried out in accordance with national personal data laws and regulations for clinical research; "*Forskrift om klinisk utprøving av legemidler til mennesker av 30.10.2009 (FOR-2009-10-30-1321)".*

The protocol will be registered in www.clinicaltrials.gov before start of the study.

12.1 Competent Authority and Ethics Committee Approval

Before starting this study the protocol will be submitted to the National Regulatory Authority (*Statens Legemiddelverk / Norwegian Medicines Agency*). The study will not start before the Regulatory Authority give approval or a favorable opinion.

The study protocol, including the patient information and informed consent form to be used, must be approved by the regional ethics committee (*Regional komite for medisinsk forskningsetikk, Helseregion Sør, REK II*) before enrolment of any patients into the study.

The investigator is responsible for informing the National Regulatory Authority of any serious and unexpected adverse events and EC also or major amendments to the protocol as per national requirements.

12.2 Handling and storage of biological material

Blood cells and serum/plasma for immunological analysis will be frozen and stored in an approved research biobank entitled "*Forskningsbiobank Infeksjonssykdommer" (REK 1.2006.1811 - S-0885, SHDNR. 09/513)* at The Department of Infectious Diseases, Oslo University Hospital HF at the Ullevål site. The research biobank will contain only patient codes, i.e. non-identifiable information.

12.3 Informed Consent Procedure

Written informed consent will be obtained from each subject before any procedures or assessments are done and after the aims, methods, anticipated benefits, and potential hazards are explained and in accordance with the national and local regulatory requirements. It will be emphasized that the participation is voluntary and that the patient is allowed to refuse further participation in the protocol whenever she/he wants. This will not prejudice the patient's subsequent care.

The subject's willingness to participate in the study will be documented in writing in a consent form, which will be individually dated and signed by the subject and by the person conducting the informed consent process. The investigator will keep the original consent forms and copies will be offered to the patients.

Written and verbal information about the study in a language understandable by the subject will be given to all subjects. The information provided must include an adequate explanation of the aims, methods, anticipated benefits, potential hazards, and insurance arrangements in force.

They will be informed as to the strict confidentiality of their patient data, but that their medical records may be reviewed for trial purposes by authorized individuals other than their treating physician.

12.4 Subject Identification

The investigator is responsible for keeping a list of all patients (who have received study treatment or undergone any study specific procedure) including patient's date of birth and personal number, full names and last known addresses.

The investigator will assure that the privacy of the subjects, including their personal identity and all personal medical information, will be maintained at all times. In CRFs and other

documents submitted to the sponsor, subjects will not be identified by their names, but by initials and allocation number.

Personal medical information may be scrutinized for the purpose of verifying data recorded in the CRF. This may be done by the regulatory authorities. Personal medical information will always be treated as confidential.

13 TRIAL SPONSORSHIP AND FINANCING

The study is financed by: **The Norwegian Research Council** through the "GlobVac" program assigned 14.01.2015 *(Grant 234493 "Therapeutic vaccination and immune modulation - new treatment strategies for the multidrug-resistant tuberculosis pandemic") and by* **Oslo University Hospital** and the **University of Oslo**.

The principle investigator (or investigating institution) will certify that the investigators and/or the investigating institutions do not have

- compensation for participation in the study from study drug manufacturer
- significant payments of other sorts from study drug manufacturer

The principal investigator has no proprietary interest in the study drug or H56 vaccine.

No initiatives towards agreements or discussions with the pharmaceutical companies that have patent rights and regulatory approvals for production and sale of COX-2i will be taken. COX-2i go off patent in 2014 to 2017 and based on experience with a second medicinal indication patent on use of COX-2i in HIV (Taskén et al, WO 2002/07721), the remaining patent life is considered too short for a potential use patent on TB to have value. Rather generic sale of COX-2i will make this class of drugs cheap and available. However, any new findings and potential inventions arising from the project work will be considered for patent protection and development in line with the IPR-policy and practise of the participating institutions. SSI has the exclusive rights to use IC31® from Intercell within the field of TB. The H56 vaccine is proprietary to SSI.

14 TRIAL INSURANCE

The Principal investigator has insurance coverage for this study through membership of the Drug Liability Association.

The clinical study subjects are insured by membership i *"Legemiddelansvarsforeningen".*

15 PUBLICATION POLICY

Upon study completion and finalization of the study report the results of this study will either be submitted for publication and/or posted in a publicly assessable database of clinical study results.

The results of this study will also be submitted to the Competent Authority and the Ethics Committee according to EU and national regulations.

All personnel who have contributed significantly with the planning and performance of the study (Vancouver convention 1988) may be included in the list of authors.

16 REFERENCES

World health organization. Global Tuberculosis report 2014.
http://www.who.int/tb/publications/global_report/en/.
Gonzalez-Juarrero M et al, Infect Immun 2001; 69:1722-8.
Hoang TT et al. PLoS One. 2009 Jun 16;4(6):e5928.
Ottenhoff TH. Int J Tuberc Lung Dis. 2012 Nov;16(11):1424-32. Review.
Keane J et al. N Engl J Med 2001; 345:1098-104.
Flynn JL et al. Immunity 1995; 2:561-72.2526.
Harari A et al. Nat Med. 2011 Mar;17(3):372-6.
Scanga CA et al. J Exp Med 2000; 192:347-58.
Dintwe OB et al., Eur J Immunol. 2013 Jun 5 doi: 10.1002/eji.201343454.
Wergeland I et al. Scand J Immunol. 2011 Mar;73(3):234-42.
Guyot-Revol V et al. Am J Respir Crit Care Med 2006 Apr 1;173(7):803-10.
Rangel Moreno J et al. Immunology. 2002 Jun;106(2):257-66.
Torgersen KM et al. Handb. Exp. Pharmacol. 2008; 186:327-363.
Brudvik KW, Taskén K. Br J Pharmacol. 2012 May;166(2):411-9. Review.
Mahic M et al. J Immunol. 2006;177:246-54.
Bryn T et al. Int Immunol. 2008;20(2):235-45.
Johansson, CC et al. AIDS 2004; 18: 6-7. 1
Kvale D et al. AIDS 2006; 20:813-820.
Pettersen FO et al. J Virol. 2011 Jul;85(13):6557-66.
Hernández-Pand et al., Clin and Exp Immunology 2006, 144: 264–272.
Peres-Buzalaf et al., Prostaglandins Leukot Essent Fatty Acids. 2011;85(2):75-81.
Vilaplana C et al. J Infect Dis. 2013 Jul 15;208(2):199-202.
Aagaard C et al. Nat Med. 2011 Feb;17(2):189-943.
Govender L et al. Vaccine. 2010 Dec 10;29(1):51-7.
van Dissel JT et al. I. Vaccine. 2011 Mar 3;29(11):2100-9.
Lin PL et al. J Clin Invest. 2012 Jan 3;122(1):303-14.
Andersen, P. Nat. Rev. Microbiol. 5, 484–487 (2007).
Olsen, A.W et al. Infect. Immun. 69, 2773–2778 (2000).
Tameris MD et al. Lancet. 2013 23;381(9871):1021-8.
Feruglio SL, Tonby K, Kvale D, Dyrhol-Riise AM. Clin Exp Immunol. 2014
Dallob A et al, J Clin Pharmacol. 2003 Jun;43(6):573-85.
Agrawal NG et al. J Clin Pharmacol. 2003 Mar;43(3):268-76.
Agrawal NG et al. J Clin Pharmacol. 2004 Oct;44(10):1125-31.
Surendra K et al. Indian J Med Res. 2011 134(6): 866–877.
Bogaty P et al., Circulation 2004; 110:934-939.

17 APPENDIX (TO PROTOCOL)

17.1 Appendix 1: Label Arcoxia 90 mg

**TIL KLINISK UTPRØVING**<br>**Studie kode: TBCOX2**<br>**Pasient initialer og kode no:** ____________<br><br>**1 TABLETT (90 mg) DAGLIG**<br><br>**Lagres ved rom temperatur**<br>Oppbevares utilgjengelig for barn<br><br>**Prosjektansvarlig:** Prof. Anne Ma Dyrhol-Riise<br>Infeksjonsmedisinsk avdeling, Oslo<br>universitetssykehus, Kirkeveien 166, 0407 Oslo<br>Tel: +4722119100 /+47 92857261<br><br>TA MED PAKNINGEN VED NESTE KONTROLL<br>Ikke kast tomme pakninger.

17.2 Appendix 2: Label Arcoxia 120 mg

TIL KLINISK UTPRØVING

Studie kode: TBCOX2
Pasient initialer og kode no: ____________

1 TABLETT (120 mg) DAGLIG

Lagres ved rom temperatur
Oppbevares utilgjengelig for barn

Prosjektansvarlig: Prof. Anne Ma Dyrhol-Riise
Infeksjonsmedisinsk avdeling, Oslo universitetssykehus, Kirkeveien 166, 0407 Oslo
Tel: +4722119100 /+47 92857261

TA MED PAKNINGEN VED NESTE KONTROLL
Ikke kast tomme pakninger.

17.3 Appendix 3: Primary label H56:IC31 5/500

H56:IC31 5/500

Study code: TBCOX2
Subject initials and no: ________________
5 µg antigen H56 + IC31 (500 nmol KLK/20 nmol ODN1a)/0.5 mL/dose.

For Single Use Only. For IM Injection Only.
Prepare according to Protocol.
For clinical trial use only.

Store cold at 2°C - 8°C
Manufactured by Statens Serum Institut
Batch: XXXXXX Use by date: YYYY.MM.DD

17.4 Appendix 4: Secondary label H56:IC31 5/500

H56:IC31 5/500

**Study code: TBCOX2**	10 single-use vials

H56:IC31 5 + 500/20 (0.8 mL sterile solution/vial)
5 µg H56 antigen + IC31 (500 nmol KLK/20 nmol ODN1a)/0.5 mL/dose
For IM injection only For clinical trial use only
Prepare according to Study Protocol
PI: Prof. MD Anne Ma Dyrhol-Riise, Oslo University Hospital, Department of Infectious Diseases, Kirkeveien 166, 0407 Oslo, Norway, Tel: +47 92857261
Manufactured by: Statens Serum Institut, 5 Artillerivej, 2300 Copenhagen S, Denmark.
Store cold at 2°C – 8°C
Batch: XXXXXX **Use by date: YYYY.MM.DD**

17.5 Appendix 5 Supplementary Table 17- Toxicity Table

Note: From final US FDA guidance: "Toxicity Grading Scale for Healthy Adult and Adolescent Subjects Enrolled in Preventive Vaccine Clinical Trials (September 2007) and for some values "Division of AIDS (DAIDS) Table for Grading the Severity of Adult and Pediatric Adverse Events Version 2.0" (November 2014). The laboratory values provided in the tables serve as guidelines and are dependent upon institutional normal parameters laboratory values.

Local Reaction to Injectable Product	Mild (Grade 1)	Moderate (Grade 2)	Severe (Grade 3)	Potentially Life Threatening (Grade 4)
Pain	Does not interfere with activity	Repeated use of non-narcotic pain reliever > 24 hours or interferes with activity	Any use of narcotic pain reliever or prevents daily activity	Emergency room (ER) visit or hospitalization
Tenderness	Mild discomfort to touch	Discomfort with movement	Significant discomfort at rest	ER visit or hospitalization
Erythema/Redness*	2.5 – 5 cm	5.1 – 10 cm	> 10 cm	Necrosis or exfoliative dermatitis
Induration/Swelling**	2.5 – 5 cm and does not interfere with activity	5.1 – 10 cm or interferes with activity	> 10 cm or prevents daily activity	Necrosis

** In addition to grading the measured local reaction at the greatest single diameter, the measurement should be recorded as a continuous variable.*
*** Induration/Swelling should be evaluated and graded using the functional scale as well as the actual measurement.*

Vital Signs *	Mild (Grade 1)	Moderate (Grade 2)	Severe (Grade 3)	Potentially Life Threatening (Grade 4)
Fever (°C) **	38.0 – 38.4	38.5 – 38.9	39.0 – 40	> 40
Tachycardia – beats per minute	101 – 115	116 – 130	> 130	ER visit or hospitalization for arrhythmia
Bradycardia – beats per minute***	50 – 54	45 – 49	< 45	ER visit orhospitalization for arrhythmia
Hypertension (systolic) - mmHg	141 – 150	151 – 155	> 155	ER visit or hospitalization for malignant hypertension
Hypertension (diastolic) - mmHg	91 – 95	96 – 100	> 100	ER visit or hospitalization for malignant hypertension
Hypotension (systolic) – mmHg	85 – 89	80 – 84	< 80	ER visit or hospitalization for hypotensive hypotensive shock
Respiratory Rate – breaths per minute	17 – 20	21 – 25	> 25	Intubation

* Subject should be at rest for all vital sign measurements.
** Body temperature, measured by ear thermometer
*** When resting heart rate is between 60 – 100 beats per minute. Use clinical judgement when characterizing bradycardia among some healthy subject populations, for example, conditioned athletes.

Systemic (General)	Mild (Grade 1)	Moderate (Grade 2)	Severe (Grade 3)	Potentially Life Threatening (Grade 4)
Nausea/vomiting	No interference with activity or 1 – 2 episodes/24 hours	Some interference with activity or > 2 episodes/24 hours	Prevents daily activity, requires outpatient IV hydration	ER visit or hospitalization for hypotensive shock
Diarrhea	2 – 3 loose stools or < 400g/24 hours	4 – 5 stools or 400 – 800g/24 hours	6 or more watery stools or > 800g/24 hours or requires outpatient IV hydration	ER visit or hospitalization
Headache	No interference with activity	Repeated use of non-narcotic pain reliever >24h or some interference with activity	Significant; any use of narcotic pain reliever or prevents daily activity	ER visit or hospitalization
Fatigue	No interference with activity	Some interference with activity	Significant; prevents daily activity	ER visit or hospitalization
Myalgia	No interference with activity	Some interference with activity	Significant; prevents daily activity	ER visit or hospitalization
Illness or clinical adverse event (as defined according to applicable regulations)	No interference with activity	Some interference with activity not requiring medical intervention	Prevents daily activity and requires medical intervention	ER visit or hospitalization

Serum	Mild (Grade 1)	Moderate (Grade 2)	Severe (Grade 3)	Potentially Life Threatening (Grade 4)
Sodium-hyponatremia, mmol/L*	130-134	125-129	121-124	≤ 120
Sodium-hypernatremia, mmol/L*	146-149	150-153	154-159	≥ 160
Potassium-hyperkalemia, mmol/L*	5.6-5.9	6.0-6.4	6.5-6.9	≥7
Potassium-hypokalemia, mmol/L*	3.0-3.4	2.5-2.9	2.0-2.4	<2.0
Glucose-hypoglycemia, mmol/L (random) **	3.6-3.8	3.0-3.5	2.5-2.9	<2.5
Glucose-hyperglycemia, mmol/L (random) **	6.1-6.8	6.9-11.0	>11.0	Insulin requirement or hyperosmolar coma
Creatinine (μmol/L)**	K: 96-145.<br>M: 106-145	146-170	171-208	> 208 or requires dialysis
Calcium-hypocalcemia mmol/L**	2.00-2.10	1.87-1.99	1.75-1.86	<1.75

Calcium -hypercalcemia mmol/L**	2.63-2.76	2.77-2.88	2.89-3.00	>3.00
Creatinine Kinase (CK), U/L***	1.25 – 1.59 x ULN	1.6-3.0 x ULN	3.1-10 x ULN	>10 x ULN
ASAT (U/L), Increased by factor***	1.1-2.5 x ULN	2.6-5.0 x ULN	5.1-10 x ULN	>10 x ULN
ALAT (U/L), Increased by factor ***	1.1-2.5 x ULN	2.6-5.0 x ULN	5.1-10 x ULN	>10 x ULN
Lactate dehydrogenase (LD) U/L**	1.1-2.5 x ULN	2.6-5.0 x ULN	5.1-10 x ULN	>10 x ULN
Bilirubin, µmol/L***	1.1-1.5 x ULN	1.6-2.0 x ULN	2.1-3.0 x ULN	>3.0 x ULN
Cholesterol, mmol/L*	6.0-6.3	6.4-7.8	>7.8	NA
Pancreatic enzyme (amylase), U/L***	1.1-1.5 x ULN	1.6-2.0 x ULN	2.1-5.0 x ULN	>5.0 x ULN
Uric acid (µmol/L)*	500-599	600-719	720-889	>890
**Hematology**	**Mild (grade 1)**	**Moderate (Grade2)**	**Severe (Grade 3)**	**Potentially Life Threatening (Grade 4)**
Hemoglobin (g/100mL) - (Female)*	10.0-10.9	9.0-9.9	7.0-8.9	<7
Hemoglobin (g/100mL) - (Female) *change from baseline value****	Any decrease -1.5	1.6-2.0	2.1-5.0	>5.0
Hemoglobin (g/100mL) -(Male) **	12.0-13.0	10.5-11.9	8.5-10.4	<8.5
Hemoglobin (g/100mL), (Male) *change from baseline value****	Any decrease -1.5	1.6-2.0	2.1-5.0	>5.0
WBC (10^9/L) - increase***	10.8-15.0	15.1-20.0	20.1-25.0	>25.0
WBC (10^9/L) - decrease***	2.5-3.5	1.5-2.4	1.0-1.4	<1.0
Platelets (10^9/L) - decrease***	125-140	100-124	25-99	<25
Lymphocytes (10^9/L) - decrease***	0.8-1.0	0.5-0.7	0.3-0.4	<0.3

ULN= Upper Level Limit of Normal range

* Division of AIDS (DAIDS) Table for Grading the Severity of Adult and Pediatric Adverse Events. Version 2.0 - November 2014. Modified according to local laboratory reference ranges for Cholesterol and Uric acid.

** Modified FDA according to local laboratory reference ranges.

*** FDA; Guidance for Industry. Toxicity Grading Scale for Healthy Adult and Adolescent Volunteers enrolled in preventive vaccine clinical trials. US Department of health and Human Services. Food and Drug Administration. Center for biologics Evaluation and Research. September 2007.

17.6 Appendix 6 DESCRIPTION OF STUDY VISITS

(If baseline day is the same day as screening day combine all examinations and blood samples).

Screening:

1. Inclusion/Exclusion evaluation (confirm that study eligibility criteria are met).
2. Written informed consent
3. Assignment of subject identification number
4. Medical history (confirm methods of contraception for females).
5. Concomitant medication: All concomitant medication (including "over-the-counter" drugs) used by the subject within 28 days of treatment start must be recorded in the CRF
6. Physical examination (focused physical examination by medical practitioner) including general apperance, BCG scar, eyes, fauces, thyroid, heart, lung, abdomen, peripheral lymph node, basic CNS examination, peripheral circulation, skin, oedema.
7. Vital signs (blood pressure, pulse and temperature), height, weight and BMI.
8. Appropriate radiology (chest X-ray/CT/MRI/UL/PET)
9. Sputum samples
10. **Blood collection for**:
    a. Serum chemistry/hematology: includes hgb, leucocytes, monocytes, lymphocytes, platelets, ESR, CRP, NA, K, CA, Kreatinin, GFR, Asat, Alat, LD, Bilirubin, Amylase, CK, gammaGT, Kolesterol, Glukose, HbA1C, Urat, Quantiferon TB. Serum HCG for females at childbearing age.
    b. **1 EDTA tube (4 ml), 1 serum tube (5 ml), 3 (one set) Quantiferon TB tubes.**
11. ECG

Visit 1-Baseline (day 0)

1. Confirm abscence of acute illness and significant syptomatic infection
2. Update medical history including use of concomitant medications
3. Focused physical examination by medical practitioner if indicated based on history
4. Vital signs: Blood pressure, pulse, temperature. Weight.
5. **Blood collection/number of tubes:**
    a. Serum chemistry/hematology: **1 EDTA tube (4 ml), 1 serum tube (5 ml).**
    b. Biobank: **1 Natrium heparin tube*(=WHOLE BLOOD), 4 CPT tubes, 1 Pax gene tube.**
6. Confirm continued eligibility for study entry and vaccination including negative hcg (for all females of child-bearing potential).
7. Randomize
8. Studydrug dispensing: Hand out and record study medication (Etoricoxib) for study arms #1 and #4. Register in drug accountability log.
9. Anti TB chemotherapy: standard combination therapy. Register medication in CRF.
10. Sputum samples.

Visit 2- (day 7)

1. Record any adverse events, including serious adverse events and adverse events of special interest occuring since previous evaluation; record any concomitant medications taken associated with treatment of the adverse event.
2. Focused physical examination by medical practitioner if indicated based on history
3. Vital signs: Blood pressure, pulse, temperature. Weight
4. **Blood collection/number of tubes:**
    a. Serum chemistry/hematology. **1 EDTA tube (4 ml), 1 serum tube (5 ml).**
    b. Blood hcg for all females of childbearing potential.
    c. Study arm#1: *Pharmacokinetics after 7-14 days of etoricoxib (see protocol).*
5. Anti TB chemotherapy: standard combination therapy. Register any changes in medication in CRF
6. Study drug dispensing and collecting: Collect and record any excessive study medication and empty medicine containers and if needed: hand out new study medication (Etoricoxib) for study arms #1 and #4. Register in drug accountability log.

Visit 3- (day 14)

1. Record any adverse events, including serious adverse events and adverse events of special interest occuring since previous evaluation; record any concomitant medications taken associated with treatment of the adverse event.
2. Focused physical examination by medical practitioner if indicated based on history
3. Vital signs: Blood pressure, pulse, temperature. Weight.
4. Sputum samples
5. **Blood collection/number of tubes**:
    a. Serum chemistry/hematology. **1 EDTA tube (4 ml), 1 serum tube (5 ml).**
    b. Blood hcg for all females of childbearing potential.
    c. Biobank **4 CPT tubes**
6. Anti TB chemotherapy: standard combination therapy. Register any changes in medication in CRF
7. Drug dispensing and collecting: Collect and record any excessive study medication and empty medicine containers and if needed: hand out new study medication (Etoricoxib) for study arms #1 and #4. Register in drug accountability log.

Visit 4- (day 28)

1. Record any adverse events, including serious adverse events and adverse events of special interest occuring since previous evaluation; record any concomitant medications taken associated with treatment of the adverse event.
2. Focused physical examination by medical practitioner if indicated based on history
3. Vital signs: Blood pressure, pulse, temperature. Weight
4. **Blood collection/number of tubes:**
    a. Serum chemistry/hematology. **1 EDTA tube (4 ml), 1 serum tube (5 ml).**
    b. Blood hcg for all females of childbearing potential.
    c. Biobank: **4 CPT tubes.**
5. Anti TB chemotherapy: standard combination therapy. Register any changes in medication in CRF
6. Drug dispensing and collecting: Collect and record any excessive study medication and empty medicine containers, and if needed: hand out new study medication (Etoricoxib) for study arms #1 and #4. Register in drug accountability log.

Visit 5- (day 56)

1. Record any adverse events, including serious adverse events and adverse events of special interest occuring since previous evaluation; record any concomitant medications taken associated with treatment of the adverse event.
2. Focused physical examination by medical practitioner if indicated based on history
3. Weight
4. Vital signs: Blood pressure, pulse, temperature
5. Sputum samples
6. **Blood collection/number of tubes:**
   a. Serum chemistry/hematology. **1 EDTA tube (4 ml), 1 serum tube (5 ml).**
   b. Blood hcg for all females of childbearing potential.
   c. Biobank: **4 CPT tubes.**
7. Anti TB chemotherapy: standard combination therapy. Register any changes in medication in CRF
8. Sputum samples
9. Appropriate radiology (chest X-ray/CT/MRI/UL/PET), but limited to chest X-ray if the disease is limited to pulmonary TB.
10. Drug dispensing and collecting: Collect and record any excessive study medication and empty medicine containers and if needed: hand out new study medication (Etoricoxib) for study arms #1 and #4. Register in drug accountability log.

Visit 6- (day 84)

1. Record any adverse events, including serious adverse events and adverse events of special interest occuring since previous evaluation; record any concomitant medications taken associated with treatment of the adverse event.
2. Focused physical examination by medical practitioner if indicated based on history
3. Vital signs: Blood pressure, pulse, temperature. Weight.
4. Sputum samples
5. **Urine collection**: Confirm continued eligibility for vaccination including negative hcg (urine pregnancy test) for all females of child-bearing potential
6. **Blood collection/number of tubes:**
   a. Serum chemistry/hematology: **1 EDTA tube (4 ml), 1 serum tube (5 ml).**
   b. Blood hcg for all females of childbearing potential.
   c. Biobank: **1 Natrium heparine tube*(=WHOLE BLOOD), 4 CPT tubes, 1 Pax gene tube.**
7. Anti TB chemotherapy: standard combination therapy. Register any changes in medication in CRF
8. *Vaccine administration study arms #2 and #4*: Confirm subject identificationnumber, check if patient is elligible to receive the study vaccine according to specific criterias. Check subject initials, date and time of dose preparation. Administer study vaccine to deltoid area by intramuscular injection. Record date and time of vaccination and which arm was vaccinated. Monitor subject for acute adverse events for at least 60 minutes after vaccination.
9. Examine site of vaccination 60 +/- 10 minutes post-immunization.
10. Drug dispensing and collecting: Collect and record any excessive study medication and empty medicine containers and if needed: hand out new study medication (Etoricoxib) for study arms #1 and #4. Register in drug accountability log.

Visit 7- (day 98)

1. Record any adverse events, including serious adverse events and adverse events of special interest occuring since previous evaluation; record any concomitant medications taken associated with treatment of the adverse event.
2. Focused physical examination by medical practitioner if indicated based on history
3. Vital signs: Blood pressure, pulse, temperature. Weight
4. **Blood collection/number of tubes:**
    - **a.** Serum chemistry/hematology: **1 EDTA tube (4 ml), 1 serum tube (5 ml).**
    - **b.** Blood hcg for all females of childbearing potential.
    - **c.** Biobank: **1 Natrium heparine tube*(=WHOLE BLOOD), 4 CPT tubes, 1 Pax gene tube.**
5. Anti TB chemotherapy: standard combination therapy. Register any changes in medication in CRF
6. Drug dispensing and collecting: Collect and record any excessive study medication and empty medicine containers and if needed: hand out new study medication (Etoricoxib) for studyarms #1 and #4. Register in drug accountability log.
7. Examine site of vaccination, study arms #2 and #4

Visit 8- (day 140)

1. Record any adverse events, including serious adverse events and adverse events of special interest occuring since previous evaluation; record any concomitant medications taken associated with treatment of the adverse event.
2. Focused physical examination by medical practitioner if indicated based on history
3. Vital signs: Blood pressure, pulse, temperature. Weight
4. **Urine collection**: Confirm continued eligibility for vaccination including negative hcg (urine pregnancy test) for all females of child-bearing potential
5. **Blood collection/number of tubes:**
    - **a.** Serum chemistry/hematology: **1 EDTA tube (4 ml), 1 serum tube (5 ml).**
    - **b.** Blood hcg for all females of childbearing potential.
    - **c.** Biobank: **1 Natrium heparine tube*(=WHOLE BLOOD), 4 CPT tubes, 1 Pax gene tube.**
6. Anti TB chemotherapy: standard combination therapy. Register any changes in medication in CRF
7. Sputum samples
8. Drug collecting/ END OF TREATMENT: Collect any excessive study medication and empty medicine containers (Etoricoxib) for study arms #1 and #4. Register in drug accountability log
9. *Vaccine administration study arms #2 and #4:* Confirm subject identificationnumber, check if patient is elligible to recieve the studyvaccine according to specific criterias. Check subject initials, date and time of dose preparation. Administer study vaccine to deltoid area by intramuscular injection. Record date and time of vaccination and which arm was vaccinated. Monitor subject for acute adverse events for at least 60 minutes after vaccination.
10. Examine site of vaccination 60 +/- 10 minutes post-immunization.

Visit 9- (day 154)

1. Record any adverse events, including serious adverse events and adverse events of special interest occuring since previous evaluation; record any concomitant medications taken associated with treatment of the adverse event.
2. Focused physical examination by medical practitioner if indicated based on history
3. Vital signs: Blood pressure, pulse, temperature. Weight

4. **Blood collection/number of tubes:**
    a. Serum chemistry/hematology: **1 EDTA tube (4 ml), 1 serum tube (5 ml).**
    b. Blood hcg for all females of childbearing potential.
    c. Biobank: **1 Natrium heparine tube*(=WHOLE BLOOD), 4 CPT tubes, 1 Pax gene tube.**
5. Anti-TB chemotherapy: standard combination therapy. Register any changes in medication in CRF
6. Examine site of vaccination, study arms #2 and #4.

Visit 10- (day 182)

1. Record any adverse events, including serious adverse events and adverse events of special interest occuring since previous evaluation; record any concomitant medications taken associated with treatment of the adverse event.
2. Focused physical examination by medical practitioner if indicated based on history
3. Vital signs: Blood pressure, pulse, temperature. Weight
4. Sputum samples
5. **Blood collection/number of tubes**:
    a. Serum chemistry/hematology. **1 EDTA tube (4 ml), 1 serum tube (5 ml).**
    b. **3 Quantiferon-TB tubes (one set)**
    c. Blood hcg for all females of childbearing potential.
    d. Biobank **4 CPT tubes. 1 Pax gene tube.**
6. Anti TB chemotherapy: standard combination therapy. Register any changes in medication in CRF.
7. Appropriate radiology (chest X-ray/CT/MRI/UL/PET), but limited to chest X-ray if the disease is limited to pulmonary TB.

Visit 11- (day 210)

1. Record any adverse events, including serious adverse events and adverse events of special interest occuring since previous evaluation; record any concomitant medications taken associated with treatment of the adverse event.
2. Focused physical examination by medical practitioner if indicated based on history
3. Vital signs: Blood pressure, pulse, temperature. Weight.
4. **Blood collection/number of tubes**:
    a. Serum chemistry/hematology. **1 EDTA tube (4 ml), 1 serum tube (5 ml).**
    b. Blood hcg for all females of childbearing potential.
    c. Biobank **4 CPT tubes**

Visit 12- End of study (day 238)

1. Record any adverse events, including serious adverse events and adverse events of special interest occuring since previous evaluation; record any concomitant medications taken associated with treatment of the adverse event.
2. Focused physical examination by medical practitioner if indicated based on history.
3. Vital signs: Blood pressure, pulse, temperature. Weight.
4. Sputum samples
5. **Blood collection/number of tubes:**
    a. Serum chemistry/hematology: **1 EDTA tube (4 ml), 1 serum tube (5 ml).**
    b. Blood hcg for all females of childbearing potential.

c. Biobank: **1 Natrium heparine tube*(=WHOLE BLOOD), 4 CPT tubes, 1 Pax gene tube.**

6. Fill out "End of study form" in eCRF.

***** Na heparin tube (green): Write time of sampling on the tube. To be processed in the LAB within 75 min after collected from patient.
