# Supplementary material for: A Phase I/II randomized trial of H56:IC31 vaccination and adjunctive cyclooxygenase-2-inhibitor treatment in tuberculosis patients
Source: Nat Commun. 2021 Nov 22;12:6774. doi: 10.1038/s41467-021-27029-6 (PMC8608791; doi:10.1038/s41467-021-27029-6)
Supplement: Supplementary file 3 — Reporting summary [file 41467_2021_27029_MOESM3_ESM.pdf]

nature research

Corresponding author(s): Dyrhol-Riise, AM, NCOMMS-21-08822A

Last updated by author(s): Sep 16, 2021

Reporting Summary

Nature Research wishes to improve the reproducibility of the work that we publish. This form provides structure for consistency and transparency in reporting. For further information on Nature Research policies, see our Editorial Policies and the Editorial Policy Checklist.

Statistics

For all statistical analyses, confirm that the following items are present in the figure legend, table legend, main text, or Methods section.

n/a	Confirmed	
☐	☒	The exact sample size (*n*) for each experimental group/condition, given as a discrete number and unit of measurement
☐	☒	A statement on whether measurements were taken from distinct samples or whether the same sample was measured repeatedly
☐	☒	The statistical test(s) used AND whether they are one- or two-sided<br>*Only common tests should be described solely by name; describe more complex techniques in the Methods section.*
☒	☐	A description of all covariates tested
☐	☒	A description of any assumptions or corrections, such as tests of normality and adjustment for multiple comparisons
☐	☒	A full description of the statistical parameters including central tendency (e.g. means) or other basic estimates (e.g. regression coefficient) AND variation (e.g. standard deviation) or associated estimates of uncertainty (e.g. confidence intervals)
☐	☒	For null hypothesis testing, the test statistic (e.g. *F*, *t*, *r*) with confidence intervals, effect sizes, degrees of freedom and *P* value noted<br>*Give P values as exact values whenever suitable.*
☒	☐	For Bayesian analysis, information on the choice of priors and Markov chain Monte Carlo settings
☒	☐	For hierarchical and complex designs, identification of the appropriate level for tests and full reporting of outcomes
☒	☐	Estimates of effect sizes (e.g. Cohen's *d*, Pearson's *r*), indicating how they were calculated

Our web collection on statistics for biologists contains articles on many of the points above.

Software and code

Policy information about availability of computer code

Data collection	Viedoc™ version 4.66.7823.20827; Microsoft Excel 2016
Data analysis	STATA version 16.1, StataCorp LLC, 4905 Lakeway Drive, College Station, Texas 77845 USA<br>GraphPad Prism version 7.02. FlowJo Software version 10.4.1 (TreeStarInc, Ashland, OR, USA).

For manuscripts utilizing custom algorithms or software that are central to the research but not yet described in published literature, software must be made available to editors and reviewers. We strongly encourage code deposition in a community repository (e.g. GitHub). See the Nature Research guidelines for submitting code & software for further information.

Data

Policy information about availability of data

All manuscripts must include a data availability statement. This statement should provide the following information, where applicable:

- Accession codes, unique identifiers, or web links for publicly available datasets
- A list of figures that have associated raw data
- A description of any restrictions on data availability

Data Availability statement: The authors declare that the data supporting the findings of this study are available in this article and Supplementary Data Files (the original and revised inclusion and exclusion criteria, priority ranking of immunogenicity readouts according to statistical plan, further details on methods, baseline participant characteristics, adherence and safety data, and results of secondary or tertiary priority immunogenicity outcomes). The full source datasets generated during and/or analyzed during the current study are available in the repository of the open science framework (https://osf.io/khvf4).

Field-specific reporting

Please select the one below that is the best fit for your research. If you are not sure, read the appropriate sections before making your selection.

- [x] Life sciences
- [ ] Behavioural & social sciences
- [ ] Ecological, evolutionary & environmental sciences

For a reference copy of the document with all sections, see nature.com/documents/nr-reporting-summary-flat.pdf

Life sciences study design

All studies must disclose on these points even when the disclosure is negative.

Sample size	No sample size calculation was done as the TBCOX2 study is an exploratory phase I/II safety study and the first of its kind. Although immunogenicity was not the primary objective of this trial, the target for inclusion, 40 patients, 10 in each study group, was based on results in Mtb-uninfected adults where H56:IC31 elicited significant differences in immunogenicity with 10 patients per group (Luabeya AK et al. Vaccine 2015; 33(33): 4130-40).
Data exclusions	All patients of the randomised set with at least one valid measurement of any of the outcome variables after baseline will be included in the full analysis set (FAS) We will perform intention to treat (ITT) analysis in the FAS, meaning that we treat participants as they were randomized and independent of adherence. Hierarchal ranking of primary and secondary priority outcomes are presented in Supplementary (Appendix) Table 1-3 and secondary and tertiary priority outcomes are presented in Supplementary Figures 2-7. Formal testing were performed on the primary priority outcomes only.
Replication	Since this is a phase 1 study with limited clinical material available the analyses were performed once with no replication of the experiments. In some experiments the analyses (Flurospot) were performed in triplicates to ensure validity. However, the laboratory protocols were validated before the experiments and Standard Operating Procedures (SOP) protocols were performed. We have also written a detailed clinical trial protocol, provide detailed protocols of the laboratory experiments and precisely predefined all statistical analyses in a statistical analysis plan, making replication of the study feasible.
Randomization	Participants were enrolled at tuberculosis diagnosis and by a computer-generated sequential allocation built into the eCRF software (ViedocTM, Viedoc Technologies AB), randomized to either; etoricoxib, H56:IC31, standard TB treatment only (controls), or etoricoxib +H56:IC31. The final treatment allocation to the four study groups was a 1:1:1:1 ratio with a randomization allocation ratio of first 2:2:1:0 and a subsequent randomization allocation ratio of 0:0:1:2. Prior to the second allocation that included the etoricoxib+H56:IC31-group, an interim safety analysis was performed when the last patient in the 2:2:1:0 groups had reached study day 98, according to protocol.
Blinding	This is a first-in human open Phase I study of Tuberculosis patients with active disease receiving 2 various intervention (vaccine and a drug). We decided to design this as an open study without blinding both due to the potential safety issue for the patients with ongoing disease and the complexity of the study design.

Reporting for specific materials, systems and methods

We require information from authors about some types of materials, experimental systems and methods used in many studies. Here, indicate whether each material, system or method listed is relevant to your study. If you are not sure if a list item applies to your research, read the appropriate section before selecting a response.

Materials & experimental systems

n/a	Involved in the study	
[ ]	[x]	Antibodies
[x]	[ ]	Eukaryotic cell lines
[x]	[ ]	Palaeontology and archaeology
[x]	[ ]	Animals and other organisms
[ ]	[x]	Human research participants
[ ]	[x]	Clinical data
[x]	[ ]	Dual use research of concern

Methods

n/a	Involved in the study	
[x]	[ ]	ChIP-seq
[ ]	[x]	Flow cytometry
[x]	[ ]	MRI-based neuroimaging

Antibodies

Antibodies used	Whole blood intracellular cytokine staining (WB-ICS) assay: CD3-AmCyan (clone SK7/Leu-4/cat.no 339186, BD), CD4-APCH7 (SK3/Leu-3a/cat.no 641398, BD), CD8-AlexaFluor488 (clone SK1/cat.no 344716, Biolegend), IFNγ-BV421 (clone B27/cat.no 562988, BD Horizon), TNFα-APC (clone Mab11/cat.no 551384, BD Pharmingen), IL2-PE (MQ1-17H12/cat.no 559334, BD Pharmingen).<br>Fluorescence immuno-spot (Fluorospot) assay (IFNγ/IL-2): Human IFNγ/IL-2 pre-coated FluoroSpot plates, 96-wells, Mabtech AB, Sweden, co-stimulant (anti-CD28 0.1mg/ml, Mabtech AB, Sweden). Anti-CD3 (Mabtech AB, Sweden).<br>Quantification of anti-H56 IgG in serum: HRP labelled Rabbit anti-human IgG (DAKO; P0214 / Cat. No. P021402-02)

Validation	The antibodies are commercial clones specific for humans and tested by the manufacturer BD Pharmingen, Biolegend, DAKO and Mabtech, titrated and validated in our lab for the Flurospot and intra cellular cytokine flow cytometry panels to give the optimal and inter-assay stable performence.

Human research participants

Policy information about studies involving human research participants

Population characteristics	Patients of both gender with age between 18-70 years with confirmed active pulmonary and/or extrapulmonary tuberculosis disease infected with drug-sensitive Mtb strains were included.
Recruitment	Patients admitted to the Dep. of Infectious Diseases and Dep. of Pulmonary Medicine, Oslo University Hospital, Norway or from other hospitals in the Oslo region or our partners from Haukeland University Hospital were screened and included according to inclusion and exclution criteria and after informed consent was given.<br>Due to the first-in-human phase I design the inclusion/exclusion criteria were strick so the majority of TB patients were not possible to include and thus included patients had none or few co-morbidities so they are not totally representative of the TB population. This could potentially impact on the relevance of the results and further studies are needed as we are discussing under limitations in our paper. Other potential self-selection bias we believe were not present since the study team had no relationship with any of the participants before inclusion. Standard TB treatment and management were also organized by the hospital and responsible clinicians.
Ethics oversight	The protocol was approved by the Regional Ethics Committee (TBCOX2, REK SØ 2015/692) and The Norwegian Medicines Agency (EudraCT Number 2014-004986-26). The study was done in compliance with the Declaration of Helsinki principles and in accordance with the International Conference on Harmonisation's Good Clinical Practices guidelines.

Note that full information on the approval of the study protocol must also be provided in the manuscript.

Clinical data

Policy information about clinical studies

All manuscripts should comply with the ICMJEguidelines for publication of clinical research and a completedCONSORT checklist must be included with all submissions.

Clinical trial registration	ClinicalTrials.gov, No: NCT02503839
Study protocol	Study protocol submitted together with manuscript and are also available on the repository of the open science framework (osf): https://osf.io/khvf4/
Data collection	The tuberculosis patients were included in the study at the study sites (Oslo University Hospital and Haukeland University Hospital) from November 2015 to December 2018 and followed at regular visits in the hospital wards or at the hospitals out-patients clinics with evaluation of tuberculosis disease and safety of the intervention at days 7, 14, 28, 56, 84, 98, 140, 154, 182, 210, and 238. Blood was drawn during the visits and transported immediately to the hospital research laboratory for further processing. Clinical data was obtained by the study nurse or study doctor from questioning and examining the patients during visits or from the hospital medical records and registrated in the eCRF.
Outcomes	The primary outcome was safety of etoricoxib and H56:IC31 alone or combined in patients that received at least one dose of etoricoxib and/or one dose of H56:IC31. Safety was assessed by the occurrence of AEs, SAEs and SUSARs. These outcomes, including incidence of solicited and unsolicited local (injection site) and systemic AE reported for 14 days after vaccination, were assessed by medical trial investigators on and included questioning of symptoms, clinical examination, radiology, microbiology and routine blood sampling. All AEs/SAEs were coded according to the Medical Dictionary for Regulatory Activities (MedDRA) coding system (https://www.meddra.org/) and evaluated for its relationship to the study interventions and severity. As participants had TB at baseline, a deterioration in the FDA toxicity grading scale (mild, moderate, severe; Supplementary Data pp. 3-5) was registered as AEs/SAEs.<br><br>The secondary outcomes were tuberculosis specific cellular (cytokine producing CD4+ T cellls) and humoral (H56 IgG) immune responses defined and priority ranked a priori depending on the hypothesized impact of the interventions an in line with comparable vaccine studies (described in detail in Supplementary Data pp. 6-7). The secondary outcome measures were assessed by Fluorescence IFNγ/IL-2 immuno-spot (Fluorospot) assay and Whole blood intracellular cytokine staining (WB-ICS) flow cytometry performed on blood cells and by ELISA quantification of anti-H56 IgG in serum.

Flow Cytometry

Plots

Confirm that:

- [x] The axis labels state the marker and fluorochrome used (e.g. CD4-FITC).
- [x] The axis scales are clearly visible. Include numbers along axes only for bottom left plot of group (a 'group' is an analysis of identical markers).
- [x] All plots are contour plots with outliers or pseudocolor plots.
- [x] A numerical value for number of cells or percentage (with statistics) is provided.

Methodology

Sample preparation	Frozen cells: Cells Peripheral blood mononuclear cells (PBMCs) were isolated from 8 mL whole blood drawn in heparinized cell preparation tubes (BD Vacutainer® CPTTM) solution by standard venipuncture. Samples were processed within 2 hours of drawing by centrifugation according to the manufacturer`s recommendations, washed with RPMI (Lonza / BioWhittaker) and cryopreserved in equal amounts of RPMI supplemented with 50% heat-inactivated fetal bovine serum (FBS) (Gibco by Life Technologies) and FBS with 20% dimethyl sulfoxide (DMSO) (Sigma Aldrich). Cells were immediately frozen at -80°C in CoolCellTM containers (Sigma Aldrich) and transferred to -150°C for storage.<br>PBMCs were thawed in 37°C by placing cryovials directly from freezer into a waterbath at 37°C for 1-2 minutes until the cell suspension detached from the wall of the cryovial and transferred to 15mL tubes containing 100µL pre-warmed RPMI 1640 enriched with 10% FBS and 1% PenStepGlutamine (100X). Cryovials were washed out with 100µL medium followed by drop-wise resuspension of cell suspension in 1mL medium and addition of medium to total 10mL for washing. Supernatants were discarded after centrifugation and cell pellet resuspended in total 1mL medium before incubation for 4 hours (37°C, 5% CO2).<br><br>Fresh cells: 1 ml of human whole blood were incubated in the presence of co-stimulants with separate peptide pools (ESAT-6, Ag85b and Rv2660c (2µg/mL, 15mer overlapping peptide, Genscript, HK limited; >85% purity) or Purified Protein Derivate (PPD, 10µg/mL, SSI) for 12h (Brefeldin A added after 7h) prior to fixation and cryopreservation (Kagina et al., 2015).
Instrument	The BD FACS Canto II flow cytometry with BD FACS Diva Software.
Software	Data analysis was performed with the FlowJo Software version 10.4.1 (TreeStarInc, Ashland, OR, USA).
Cell population abundance	PBMC count and viability were assessed on stained cells using MuseTM Count & Viability kit and analyzed by the MuseTM Cell Analyzer (Merck, Germany) and the viability of PBMC was typically > 90%.<br>The T cell subsets were determined by the following antibodies by flow cytometry analyses; CD3-AmCyan (clone SK7/Leu-4/ cat.no 339186, BD), CD4-APCH7 (SK3/Leu-3a/cat.no 641398, BD), CD8-AlexaFluor488 (clone SK1/cat.no 344716, Biolegend)
Gating strategy	The immunological readout Cytokine+ CD4 T cells denoted producers of any of the measured cytokines. Other subpopulations are specified according to their cytokine production. FS/SS gated to singel cell population further to CD3+ cells and to CD4+ and CD8+ and finnally to IL2+, TNF+, IFNg+ CD4+, resp.

☒ Tick this box to confirm that a figure exemplifying the gating strategy is provided in the Supplementary Information.
